# Systematic review of Group B Streptococcal capsular types, sequence types and surface proteins as potential vaccine candidates

**DOI:** 10.1016/j.vaccine.2020.08.052

**Published:** 2020-10-07

**Authors:** Fiorella Bianchi-Jassir, Proma Paul, Ka-Ning To, Clara Carreras-Abad, Anna C. Seale, Elita Jauneikaite, Shabir A. Madhi, Neal J. Russell, Jenny Hall, Lola Madrid, Quique Bassat, Gaurav Kwatra, Kirsty Le Doare, Joy E. Lawn

**Affiliations:** a Maternal, Adolescent, Reproductive & Child Health (MARCH) Centre, London School of Hygiene & Tropical Medicine, London, UK; b Dept of Infectious Disease Epidemiology, London School of Hygiene & Tropical Medicine, London, UK; c Paediatric Infectious Diseases Research Group and Vaccine Institute, St George's, University of London, UK; d Department of Infectious Disease, Imperial College London, UK; e Department of Infectious Disease Epidemiology, School of Public Health, Imperial College London, UK; f Medical Research Council: Respiratory and Meningeal Pathogens Research Unit, University of the Witwatersrand, Faculty of Health Sciences, Johannesburg, South Africa; g Department of Science and Technology/National Research Foundation: Vaccine Preventable Diseases, University of the Witwatersrand, Faculty of Health Sciences, Johannesburg, South Africa; h EGA Institute for Women’s Health, University College London Institute for Women’s Health, London, UK; i ISGlobal, Hospital Clínic - Universitat de Barcelona, Barcelona, Spain; j Centro de Investigação em Saúde de Manhiça (CISM), Maputo, Mozambique; k ICREA, Pg. Lluís Companys 23, 08010 Barcelona, Spain; l Pediatric Infectious Diseases Unit, Pediatrics Department, Hospital Sant Joan de Déu (University of Barcelona), Barcelona, Spain; m Consorcio de Investigación Biomédica en Red de Epidemiología y Salud Pública (CIBERESP), Madrid, Spain; n Department of Clinical Microbiology, Christian Medical College, Vellore, India; o Medical Research Council at the London School of Hygiene & Tropical Medicine, Uganda

**Keywords:** Group B *Streptococcus*, Vaccine, Serotypes, Multi locus sequence typing, Whole genome sequencing, Maternal, Neonatal, Stillbirth, Adult

## Abstract

**Background:**

21 million pregnant women worldwide (18%) are estimated to carry Group B *Streptococcus* (GBS), which is a risk for invasive disease in newborns, pregnant women, and stillbirths. Adults ≥ 60 years or with underlying health conditions are also vulnerable to invasive GBS disease. We undertook systematic reviews on GBS organism characteristics including: capsular polysaccharide (serotype), sequence type (multi-locus sequence types (MLST)), and virulence proteins. We synthesised data by at-risk populations, to inform vaccine development.

**Methods:**

We conducted systematic reviews and *meta*-analyses to estimate proportions of GBS serotypes for at risk populations: maternal colonisation, invasive disease in pregnant women, stillbirths, infants 0–90 days age, and older adults (≥60 years). We considered regional variation and time trends (2001–2018). For these at-risk population groups, we summarised reported MLST and surface proteins.

**Results:**

Based on 198 studies (29247isolates), 93–99% of GBS isolates were serotypes Ia, Ib, II, III, IV and V. Regional variation is likely, but data gaps are apparent, even for maternal colonisation which has most data. Serotype III dominates for infant invasive disease (60%) and GBS-associated stillbirths (41%). ST17 accounted for a high proportion of infant invasive disease (41%; 95%CI: 35–47) and was found almost exclusively in serotype III strains, less present in maternal colonisation (9%; 95%CI:6–13),(4%; 95%CI:0–11) infant colonisation, and adult invasive disease (4%, 95%CI:2–6). Percentages of strains with at least one of alp 1, alp2/3, alpha C or Rib surface protein targets were 87% of maternal colonisation, 97% infant colonisation, 93% infant disease and 99% adult invasive disease. At least one of three pilus islands proteins were reported in all strains.

**Discussion:**

A hexavalent vaccine (serotypes Ia, Ib, II, III, IV and V) might provide comprehensive cover for all at-risk populations. Surveillance of circulating, disease-causing target proteins is useful to inform vaccines not targeting capsular polysaccharide. Addressing data gaps especially by world region and some at-risk populations (notably stillbirths) is fundamental to evidence-based decision-making during vaccine design.

## Highlights

• Most comprehensive review of Group B Streptococcal serotypes through 2018.
• First systematic review of Group B Streptococcal strain type and protein data.
• Theoretically candidate vaccines may protect against 93-99% disease-causing strains.
• More studies on GBS strains in low- and middle-income countries are needed.

## 1 Introduction

Deaths for children before their fifth birthday have reduced from an estimated 12.6 million child deaths in 1990 to 5.3 million deaths in 2017 [1]. This decline is in part attributed to high coverage of childhood immunisations [2], although infectious causes still account for at least a quarter of under-five child deaths. Almost half of child deaths worldwide occur in the neonatal period [2], and approximately two thirds within the first three months of life, so more attention is required to meet Sustainable Development Goals (SDG) by 2030 [3]. Hence there is more urgency in addressing the confluence of neonatal deaths and infections, with maternal immunisation as one strategy [4].

Group B Streptococcus (GBS) or *Streptococcus agalactiae* is a Gram-positive bacterium, commonly colonising the gastrointestinal tract. Worldwide around 18% of pregnant women are colonised by GBS – totalling over 21 million women each year [5]. Colonisation carries risk of invasive GBS disease in babies (before or after birth), and pregnant/postnatal women. Ascending GBS infections in pregnant women can lead to stillbirth or preterm birth [6], [7], [8], [9], [10]. GBS invasive disease burden was estimated to be 319,000 infants in 2015 [uncertainty range (UR), 119000–417000], resulting in 90,000 (UR 36000–169000) deaths, at least 57,000 (UR 12000–104000) fetal infections/stillbirth, and up to 3.5 million preterm births [11]. Maternal invasive GBS disease during pregnancy/postnatally affected at least 33,000 (UR 13000–52000) women [11]. GBS disease in the elderly was not included in previous burden estimates, but is increasingly recognised public health issue, causing morbidity and mortality [12], [13], [14].

Intrapartum antibiotic prophylaxis (IAP) to reduce early-onset GBS disease (EOGBS), and in the United States of America (USA), implementation since 1990 has been associated with > 80% reduction in EOGBS incidence [15]. However, most low and lower-middle-income countries (LMIC) do not have a specific IAP policy [16], and scale-up is likely to be challenging [17]. Additionally, IAP has no effect on reducing late-onset GBS disease (LOGBS) [13], and would not be expected to prevent GBS associated stillbirth or preterm birth, due to IAP administration is given after onset of labor and/or rupture of membranes. In contrast, an effective GBS vaccine could prevent invasive GBS disease across all at-risk population groups, including mother, fetus, infant, and the elderly or immunocompromised [11].

Several GBS vaccine candidates are in development, including multivalent GBS bacterial capsular polysaccharide (CPS) -CRM_197_ conjugate vaccines [18], CPS-protein conjugates vaccines [19], and multivalent adjuvanted protein vaccines (NCT03807245). Placental transfer of anti-CPS specific GBS antibodies from the mother to the fetus reduces the risk of invasive GBS disease with evidence of protection against both EOGBS and LOGBS [20]. Multivalent CPS-protein conjugate vaccines induce an increased CPS-specific IgG response [21]. Since capsular-type switching is possible [22], there is also interest in developing GBS protein-based vaccines, and protein-based vaccines are undergoing preclinical studies [23] or Phase I/II clinical trials (NCT03807245).

GBS isolates are classified by their CPS into ten serotypes: Ia, Ib, II-IX. Strains are assigned to a sequence type (ST), through multi-locus sequence typing (MLST), based on allelic variation of seven housekeeping genes [24] further grouping similar allelic profiles into clonal complexes (CC). Molecular techniques, including MLST and whole-genome sequencing (WGS), have enabled better characterisation of GBS, and highlighted that different capsular serotypes are present within the same ST. The latter is especially important for capsule polysaccharide-based vaccines as certain sequence types have been more associated with GBS human disease, such as ST17. A strong association between ST17 and severe neonatal and young infant disease has been demonstrated [25], [26], [27]. Five major clonal complexes in humans (CC1, CC10, CC17, CC19, and CC23) are associated with colonisation and invasiveness of GBS [28], [29], [30]. GBS strains can also be classified on the basis of surface proteins, such as Alp family proteins, serine-rich repeat proteins, C5a peptidase, and pilus islands [31]. Proteins such as hvgA, Rib and pilus island proteins have also been associated with invasiveness of GBS strains [32], [33], [34], [35].

Development of GBS vaccines should be informed by evidence from all over the world, considering all the relevant at-risk populations, and data not just on serotype distribution, but also strain types and conserved protein targets. This study aims to inform GBS vaccine design, based on systematic reviews and *meta*-analyses regarding GBS serotypes, considering geographical variation and time trends. We update literature searches [36] regarding serotype data for 1). maternal colonisation, 2). maternal invasive disease and 3). infant invasive disease. Additionally, we expand the previously covered at-risk populations to include 4). stillbirths and 5). disease in adults over 60 years old. We also expanded the searches remit from serotypes alone to cover sequence types and specific surface protein genes.

## 2 Methods

### 2.1 Case definitions

Definitions for maternal GBS colonisation, invasive GBS disease, EOGBS, LOGBS, and maternal invasive GBS disease, have been detailed previously [5], [9], [36], [37], [38] (supplementary Table S1). For stillbirth, we used the World Health Organisation definition for international comparison and reporting (birth of a fetus with no signs of life at ≥ 28 weeks’ gestation or weighing 1000 g), and the International Classification of Disease definition (birth of a fetus with no signs of life at 22 weeks or more gestation or weighing > 500 g) [39]. GBS invasive disease in older adults was defined after 60 years of age.

### 2.2 Search strategy and inclusion criteria

We performed systematic literature searches in Medline, Embase, Scopus, the World Health Organisation Library Information System (WHOLIS), and Literature in Health Sciences in Latin America and the Caribbean (LILACS). Searches were limited to humans with no language restrictions. All searches were to March 2019. For maternal colonisation and infant GBS disease, the literature searches were from 2017 to current, updating previously conducted literature reviews [5], [37]. All searches included “Group B Streptococcus” or “*Streptococcus agalactiae*”, combined with “serotype” or with “sequencing” or “MLST”. Specific search terms per database are in supplementary Table S2. Medical subject heading (MeSH) terms were used where possible. Snowballing identified additional studies. We included studies reporting serotypes, sequence types and protein expression either from observational studies or pooled laboratory samples, and presented proportions among cases. Inclusion and exclusion criteria are detailed in supplementary Table S3.

Database searches, screening for duplicates and titles for eligibility, and selection of abstracts were performed by FBJ for serotype data updates and KT, CCA, KLD and EJ for MLST/virulence factors. Assessment of full-length articles, and data extraction, was done by two independent investigators, FBJ and PP for serotype data and KT, KLD and EJ for MLST/virulence factors. If there was discrepancy between two reviewers, a third investigator made the final decision.

### 2.3 Data abstraction

Data were extracted into standardised Excel forms including: year of study, country, study site, study design, definitions used (for invasive GBS disease or EOGBS/LOGBS or stillbirth), age of patients from whom samples were taken, site of isolation (e.g. if vaginal, rectal or both, for invasive disease whether isolate was from blood or CSF cultures), and serotyping methods. United Nations SDG region classification was used for world regions [40]. For infant GBS disease studies, we abstracted serotype data from cases with sepsis or bacteraemia and meningitis, when available. For MLST data we abstracted additionally infant colonisation, and all adult invasive disease data (18yearsandolder). Reported GBS genotypic data were summarised based on most common ST and/or CC, number of isolates that were ST-17, detected virulence genes, if WGS was done, and the related serotypes with CCs, and presence of proteins of interest and pilus islands. Data collected from previous review/*meta*-analyses were input as previously reported.

### 2.4 Analyses

Data were imported to STATA version 14 software (StataCorp 2014, Texas) for *meta*-analyses. We used random-effects *meta*-analyses to estimate the proportion of each serotype with the number of isolates serotyped as the denominator, using the DerSimonian and Laird method for pooled proportion estimates with 95% confidence intervals [41]. Pooled estimates for each serotype were then transformed to percentages (pooled percentages) and adjusted (scaled up or down) to fit 100% for the total of all serotypes (adjusted percentages).

For each at-risk population group, we calculated proportions of six major clonal complexes (CC1, CC10, CC17, CC19, CC23 and CC12), of six surface proteins (alp1, alp2, alp3, alp4, alpha C and rib), and the three pilus islands alleles (PI-1, PI-2a, PI2b). Within reported CCs, surface proteins and pilus islands, we analysed ten serotypes distribution. We undertook a *meta*-analysis for ST17 proportion reported for each at-risk population group.

Regarding time trend analysis, studies were classified according to median year the samples were taken, into 4 time periods: pre-2001, 2001 to 2006, 2007 to 2012, and 2013 to 2018. Sensitivity analyses were done to assess significant changes in serotype distribution when excluding studies that only tested/reported < 5 serotypes.

## 3 Results

### 3.1 Data included

**Fig. 1:**
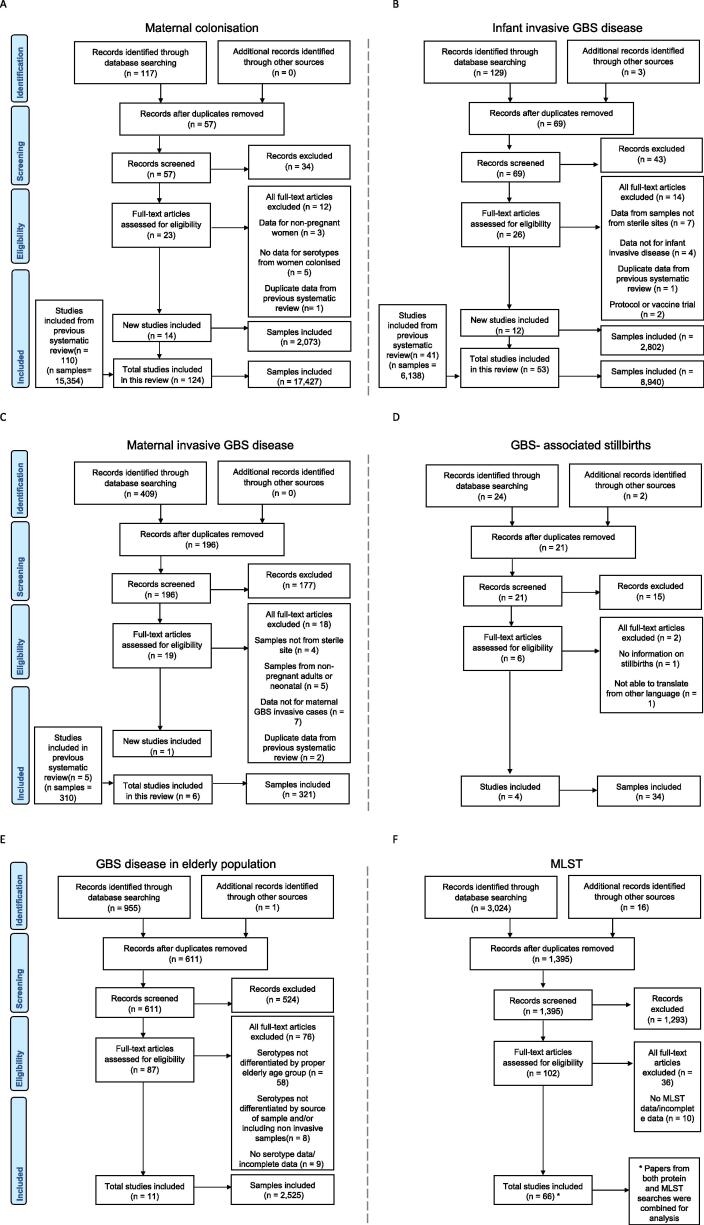

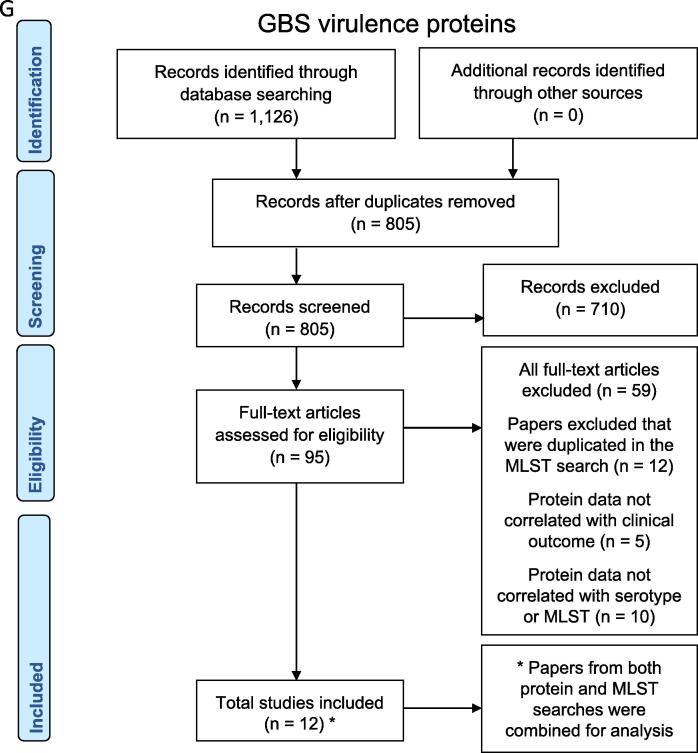
Data search and included studies on group B *Streptococcus*: A. serotypes for maternal colonisation, B. serotypes for infant invasive disease, C. serotypes for maternal invasive GBS disease, D. serotypes for GBS- associated stillbirth, E. serotypes for streptococcal invasive disease in the elderly, F. MLST data, and G. virulence proteins. * Papers from both protein and MLST searches were combined for analysis.

**Fig. 2:**
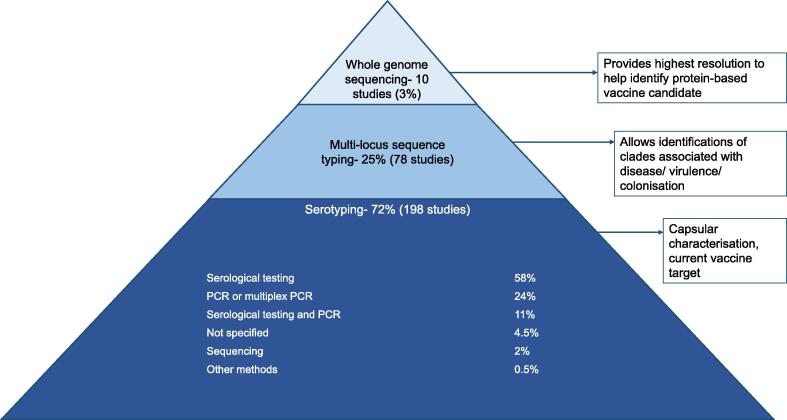
Characterisation methods used for description and investigation of GBS strains.

The total number of studies included in this review (and the corresponding number of isolates reported) were: 124 (n = 17,427 isolates) for maternal colonisation, 6 (n = 321 isolates) for maternal invasive GBS disease, 4 (n = 34 isolates) for stillbirth, including two studies from unpublished dataset obtained from investigators (QB and GK), 53 (n = 8,940 isolates) for infant GBS disease, and 11 (n = 2,525 isolates) for adults age 60 and older (Fig. 1). Six studies reported serotypes for neonatal sepsis/bacteraemia cases (n = 623 isolates) and meningitis cases (n = 180 isolates) [42], [43], [44], [45], [46], [47]. For MLST and surface protein genes, 78 studies (n = 7193 isolates) were included in descriptive analysis. Details from data abstracted are described in supplementary Table S4 and S5. We found 198 studies with data on capsular serotypes of GBS strains and 78 studies that characterised sequence types, clonal complexes or virulence genes by polymerase chain reaction (PCR) (Fig. 2). Serotyping methods used were: serological methods (58%), PCR (24%), and latex agglutination accompanied by PCR (11%).

### 3.2 Data availability of GBS CPS, MLST/CC and virulence proteins

In total, 41% of GBS serotype data came from Europe and northern America (81/198). Data from eastern Asia (36/198), sub-Saharan Africa (27/198), Latin America and the Caribbean (17/198), and south/south-eastern Asia (15/198) each represented<18% of the reported studies. The country with the most studies was China (n = 14), mainly from more recent years (10/14 in last 10 years), followed by USA, then Canada (11and10studies,respectively).

Regional variation was observed in data availability by at-risk population groups. Maternal colonisation data were available in almost all regions (except Oceania and central Asia) (Supplementary Fig. S1). There were no other infant invasive disease studies reporting serotypes from southern Asia which accounts for almost half the world’s births (Supplementary Fig. S2). Studies reporting early and late onset infant sepsis and meningitis serotypes were only from eastern Asia (n = 3) and Europe (n = 3). Isolates causing maternal invasive disease were reported only from northern America and one study from China (Supplementary Fig. S3). The four studies with data for GBS-associated stillbirth were from Kenya, Mozambique, South Africa and Canada (Supplementary Fig. S4). For older adults, all data were from Europe and northern America, except for two studies from south-eastern Asia (both from Malaysia), and one study from Latin America (Argentina) (Supplementary Fig. S5).

Regarding virulence proteins and MLST by PCR and/or limited sequencing, around 45% (35/78) came from Europe, other regions were eastern Asia 29% (23/78), sub-Saharan Africa 9% (7/78), northern America 9% (7/78), and western Asia 3% (2/78). One study each from Latin America and the Caribbean, northern Africa, Australia, and southern Asia. (Supplementary Fig. S6).

### 3.3 *Results of GBS serotypes,* MLST and virulence proteins data by at-risk population group

**Fig. 3:**
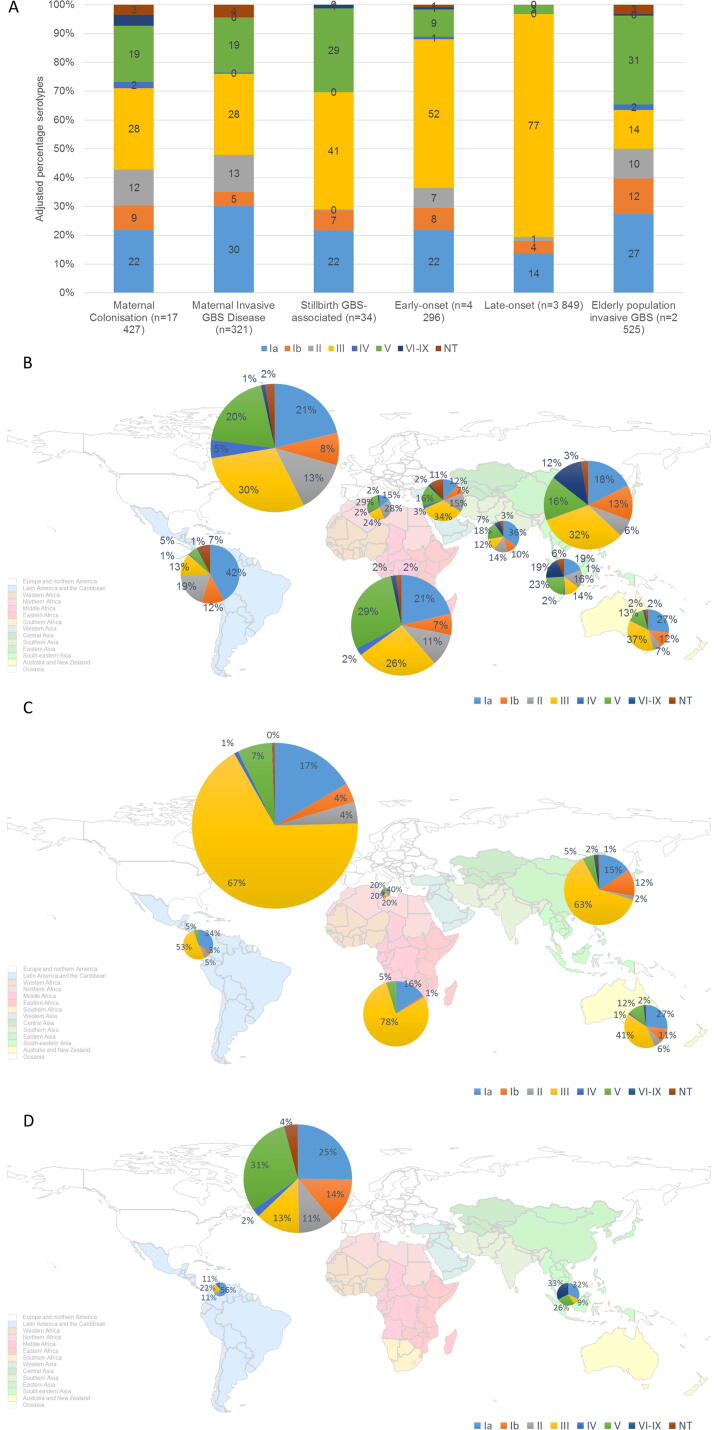
A. Distribution worldwide of GBS serotypes by risk population group, B. distribution of GBS serotypes by regions from maternal colonisation isolates (n = 17427), C. distribution of GBS serotypes by regions from infant invasive disease and stillbirth isolates (n = 8974), D. distribution of GBS serotypes by regions from invasive disease in elderly population isolates (n = 2525). Results are of adjusted percentages. Size of pie charts correspond to number of isolates from each region. Scale of pie charts not the same between figures B, C and D.

**1. *Maternal colonisation (n = 17,427 isolates)***: The most common GBS serotype globally was serotype III with 25% (95%CI: 23–27), then serotype Ia with 19% (95%CI: 17–21) (Fig. 3A). Serotype distribution varied by region (Fig. 3B). Serotypes III, Ia and V were the three most common serotypes in several regions (Europe and northern America, eastern Asia, southern, eastern/central Africa, and Australia/ New Zealand). Serotype III was less common in Latin America and the Caribbean, south-eastern Asia, southern Asia, and western Africa, with 11% (95%CI :6–17), 11% (95%CI: 6–17), 11% (95%CI: 6–15), and 13% (95%CI: 8–18), respectively. Serotype IV was more common in Europe and northern America with 4% (95%CI: 3–6) (n = 325/6,659), and in southern Africa with 3% (95%CI: 2–5) (n = 64/1,897) than other regions (Supplementary Fig. S7&S8). Serotypes VI-IX were rare in Europe and northern America (1% (95%CI: 0–2), but in south-eastern Asia, eastern Asia, southern Asia, and western Africa (with most data from a Ghanaian study n = 83) [48] they were more common at 16% (95%CI: 6–29), 11% (95%CI: 4–19), 6% (95%CI: 1–15), and 15% (95%CI: 0–83), respectively.

Twenty-six studies described GBS maternal colonisation by MLST or virulence proteins (n = 4,019 isolates). CC19 was the most common clonal complex reported for maternal colonisation with 22%, followed by 19% CC23, 17% CC1, and 15% CC17. Within these clonal complexes the most common serotype was III representing 98% of CC17 strains and 70% of CC19, while serotype Ia predominated in CC23 with 65% and serotype V in CC1 with 52% (Supplementary Fig. S9). The pooled proportion of ST17 for maternal colonisation isolates was 9% (95%CI: 6–13).

Forty percent of maternal colonisation strains had Rib protein gene, from which 66% were serotype III (66%). Alpha C was also reported on 28% of strains and alp1/epsilon on 26% of strains, of which 59% were serotype Ia (Supplementary Fig. S10). 87% of strains had at least one of alp 1, alp2/3, alpha C or Rib protein targets. The combination of PI-1 and PI-2a predominated among maternal colonisation strains with 38%, followed by only PI-2a in 32%. The biggest proportions of serotypes with pilus island protein genes were serotype Ia at 69% (PI-2a only) and serotype III at 85% of strains (PI-1 and PI-2b) (Supplementary Fig. S11).

***Newborn/Infant colonisation (n = 159 isolates)***: Similar to maternal colonisation, CC19 was the commonest clonal complex identified with 39%, followed by CC23 with 23%, but less CC17 than maternal colonisation with only 5%. 60% of CC19 strains expressed the serotype III CPS and 52% of CC23 strains expressed Ia CPS (Supplementary Fig. S9). The pooled proportion of ST17 for infant colonisation isolates was 4% (95%CI: 0–11). Different from maternal colonisation, the most common surface proteins genes were alp 1/epsilon and alp2/alp3 with 27% each, followed by Rib with 23%, and alpha C (18%). 90% of strains with the Rib protein gene and 92% of strains with alp2/alp3 belonged to serotype III (Supplementary Fig. S10). 97% of the strains had at least one of alp1, alp2/3, alpha C or rib protein targets. There was only one study (n = 35) of pilus island protein genes from infant colonisation, showing 77% had PI-1 and PI-2a, followed by 14% strains with only PI-2a.

***2. Maternal invasive GBS disease (n = 321 isolates)***: The most common disease-associated serotype was Ia accounting for 27% (95%CI: 22–33) of cases, followed by serotype III with 26% (95%CI: 20–32) and V 17% (95%CI: 10–27) (Fig. 3A). Noting only one study from eastern Asia (n = 11 isolates) [44], the main difference with northern America was more serotypes III and V and less serotype Ia, in eastern Asia (Supplementary Fig. S12).

MLST data for maternal disease were available from only one study (n = 29 isolates) [49], reporting the commonest clonal complex as CC23 (41%) followed by CC17 (24%), where 83% of CC23 were serotype Ia and 86% of CC17 were serotype III (Supplementary Fig. S13). This study did not specify the number of strains that were ST17 or report surface proteins or pilus islands data.

***3. Infant invasive GBS disease (n = 8,940 isolates)***: In EOGBS (n = 4,296) and LOGBS (n = 3,849), serotype III was the most common with 46% (95%CI: 42–51) and 70% (95%CI: 64–75) of disease associated with this serotype, respectively. Serotype Ia was the second most frequently detected serotype in both EOGBS and LOGBS, with 20% (95%CI: 17–22) and 12% (95%CI: 10–15), respectively. For meningitis cases there was a similar distribution of serotypes between EOGBS and LOGBS (Table 1), with serotype III predominating with 78% (95%CI: 58–94) and 82% (95%CI: 74–89), respectively. In sepsis/bacteraemia cases, serotype distribution varied more in EOGBS than in LOGBS, with more EOGBS cases caused by serotypes II, V and VI-IX.

**Table 1:** Distribution of GBS serotypes of infant invasive isolates for early-onset and late-onset sepsis/bacteremia and meningitis cases. (NT: non-typeable; CI: confidence interval).

			n Ia	Pooled percentage (95% CI)	n Ib	Pooled percentage (95% CI)	n II	Pooled percentage (95% CI)	n III	Pooled percentage (95% CI)	n IV	Pooled percentage (95% CI)	n V	Pooled percentage (95% CI)	n VI-IX	Pooled percentage (95% CI)	n NT	Pooled percentage (95% CI)
Early-onset GBS disease	**Sepsis/bacteraemia**		**74**	**12.3(5.0**–**21.5)**	**28**	**6.8(1.9**–**13.5)**	**34**	**5.7(2.5**–**9.9)**	**189**	**44.1 (30.9**–**57.8)**	**2**	**0 (0.0**–**0.0)**	**38**	**7.3 (4.3**–**10.8)**	**8**	**1.7 (0.0**–**10.7)**	**20**	**3.0 (0.5**–**6.8)**
		Europe	56	16.4 (11.9–21.4)	14	1.9 (0.1–5.4)	28	6.5 (3.4–10.3)	157	52.9 (46.4–59.3)	2	0 (0.0–0.1)	26	5.9 (2.9–9.6)	2	0 (0.0–0.3)	13	1.5 (0.0–7.0)
		Eastern Asia	18	6.0 (0.0–33.5)	14	13.6 (6.8–22.0)	6	4.7 (0.0–17.7)	32	34.5 (11.2–62.0)	0	0 (0.0–1.0)	12	14.0 (3.4–28.8)	6	5.6 (1.3–12.0)	7	6.1 (1.5–12.7)
	**Meningitis**		**9**	**8.1 (0.3**–**21.5)**	**4**	**1.1 (0.0**–**21.6)**	**0**	**0 (0.0**–**0.0)**	**40**	**78.0 (57.6**–**94.2)**	**0**	**0 (0.0**–**0.0)**	**2**	**0 (0.0**–**3.6)**	**0**	**0 (0.0**–**0.1)**	**0**	**0 (0.0**–**0.0)**
		Europe	7	9.6 (0.2–25.8)	0	0 (0.0–0.0)	0	0 (0.0–0.0)	32	84.6 (67.0–97.4)	0	0 (0.0–0.0)	2	0 (0.0–6.9)	0	0 (0.0–0.0)	0	0 (0.0–0.0)
		Eastern Asia	2	4.9 (0.0–34.4)	4	23.8 (1.0–56.9)	0	0 (0.0–9.4)	8	65.1 (14.3–100.0)	0	0 (0.0–9.4)	0	0 (0.0–9.4)	0	0 (0.0–14.5)	0	0 (0.0–9.4)
Late-onset GBS disease	**Sepsis/bacteraemia**		**29**	**11.1 (6.9**–**16.0)**	**14**	**4.2 (1.5**–**7.9)**	**6**	**0.9 (0.0**–**3.4)**	**165**	**71.4 (62.5**–**79.6)**	**1**	**0 (0.0**–**0.0)**	**7**	**1.3 (0.0**–**7.3)**	**1**	**0 (0.0**–**1.6)**	**7**	**1.1 (0.0**–**4.4)**
		Europe	16	12.1 (6.3–19.0)	5	2.1 (0.1–7.3)	3	1.3 (0.0–5.2)	90	74.5 (66.0–82.3)	1	0 (0.0–0.4)	4	1.0 (0.0–7.2)	0	0 (0.0–2.1)	3	0.7 (0.0–8.2)
		Eastern Asia	13	10.1 (4.3–17.4)	9	6.3 (1.7–12.8)	3	2.5 (0.0–13.4)	75	60.4 (35.4–83.0)	0	0 (0.0–1.0)	3	4.1 (0.0–25.7)	1	0.4 (0.0–3.5)	4	1.8 (0.0–6.4)
	**Meningitis**		**13**	**6.9 (2.0**–**13.4)**	**3**	**0 (0.0**–**2.5)**	**1**	**0 (0.0**–**0.9)**	**99**	**82.1 (73.7**–**89.4)**	**0**	**0 (0.0**–**0.0)**	**5**	**0.8 (0.0**–**4.8)**	**1**	**0 (0.0**–**3.7)**	**3**	**0 (0.0**–**2.5)**
		Europe	8	5.2 (0.6–12.4)	1	0 (0.0–1.4)	1	0 (0.0–1.4)	77	85.8 (76.6–93.4)	0	0 (0.0–0.0)	4	1.3 (0.0–6.6)	1	0 (0.0–7.5)	1	0 (0.0–1.0)
		Eastern Asia	5	12.7 (1.2–30.3)	2	3.8 (0.0–16.2)	0	0 (0.0–4.7)	22	69.8 (51.1–86.1)	0	0 (0.0–4.7)	1	0.7 (0.0–14.7)	0	0 (0.0–5.5)	2	3.7 (0.0–16.0)

Fig. 3C shows geographical distribution of serotypes for infant GBS disease (0-89daysonset) and GBS-associated stillbirth isolates all together. A similar serotype distribution is seen in most regions (7/8), with serotype III ranging from 41% (95%CI: 36–46) in Australia/New Zealand (n = 365) to 79% (95%CI: 54–97) in eastern Africa (n = 112) (Supplementary Fig. S14&S15).

GBS disease in infants had 49 studies reporting MLST and/or virulence proteins data (n = 3995 isolates). CC17 (42%) was the commonest clonal complex. In contrast to infant colonising strains CC19 and CC23 accounted for only 18% and 15% of infant disease respectively. ST17 associated with infant invasive disease was 41% (95%CI: 35–47). ST17 was more associated with LOGBS (69% (234/337)) than EOGBS (30% (101/337)) (Supplementary Table S6) [22], [25], [27], [44], [50], [51], [52], [53], [54], [55], [56], [57].

Rib was the surface protein gene most likely to be reported in infant invasive disease (54%), particularly strains belonging to serotype III (95%) (Supplementary Fig. S16). Other proteins genes in infant invasive disease strains were alp1/epsilon with 17%, alpha C 16%, and alp2 and alp3 8% (61/754). 93% of the strains had at least one of alp1, alp2/3, alpha C or Rib protein targets. The combination of PI-1 and PI-2b was common in infant invasive strains (46%), from which 97% were serotype III, followed by the combination of PI-1 and PI-2a with 32% (Supplementary Fig. S17). HvgA presence was analysed in six studies [22], [25], [58], [59], [60], all of which were present in ST17 strains, and was associated with infant disease.

***4. GBS-associated stillbirth (n = 34 isolates):*** Serotypes III and V were both the most frequently reported with 34% (95%CI: 17–53) and 24% (95%CI: 9–42), respectively (Fig. 3A). There were no data on GBS strains using MLST or WGS for stillbirth-associated GBS.

***5. Invasive GBS disease in older adults (n = 2525 isolates)***: Serotype V was the most common with 25% (95%CI: 20–31) followed by serotype Ia with 23% (95%CI: 19–27), and serotype III 11% (95%CI:9–14) (Fig. 3A). Fig. 3D shows differences between regions in the distribution of GBS serotypes in the elderly population, though Latin America and the Caribbean results are based on one study (n = 9) and south-eastern Asia on two (n = 16). Nonetheless, in south-eastern Asia, similar to maternal colonisation (the only other risk group that has data from this region), serotypes VI to IX have a higher presence than in other regions, representing 31% (95%CI: 9–57) of the isolates (4 serotype VI and 1 serotype VII). In Europe and northern America and in south-eastern Asia invasive disease in the elderly was caused less by serotype III, compared to invasive disease in the infant, mother or stillborn infant.

MLST and/or virulence protein data were reported in 17 studies (n = 2,108 isolates) for adult invasive disease (≥18 years). Commonest CCs were CC1 (37%), being mostly serotypes V (39%) and Ib isolates (23%); CC23 (18%), with most of the strains belonging to serotype Ia (90%), and CC10 (18%) of which 84% were serotype Ib (Supplementary Fig. S13). The proportion of strains reported to belong to ST17 was 4% (95%CI: 2–6). Adult invasive strains had mostly alp3 protein gene (29%), alpha C (25%), and Rib (23%). The majority of strains with alp3 gene were serotype V (82%), while those with Rib were mostly serotype III (72%) (Supplementary Fig. S16). 99% of strains had at least one of alp1, alp2/3, alpha C or Rib protein targets. High prevalence was found of the combination of pilus islands PI-1 and PI-2a (61%), and strains with only PI-2a (30%). Strains with pilus island proteins genes most commonly belonged to serotypes V (29%) and Ia (72%) (Supplementary Fig. S17).

### 3.4 Time trends for GBS serotypes

There has been an increase in the number of studies published on GBS serotypes in the last twenty years from 17 studies in pre-2001 to 93 studies in 2013–2018, especially for maternal colonisation and infant GBS disease in the last period. Although the majority of studies and isolates were from countries in the UN classified developed region [40], over time, the number of studies from all other regions have been increasing, to the point that in the last time period, there were more published studies from countries in other regions than from the developed region.

**Fig. 4:**
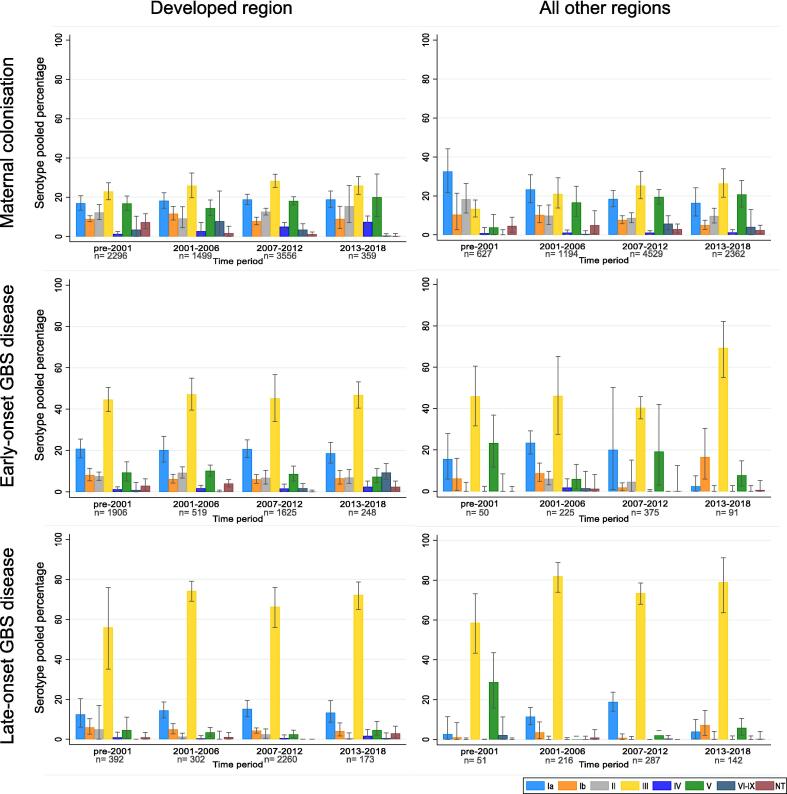
Time variation of GBS serotypes (adjusted percentages with confidence intervals) for maternal colonisation, early-onset GBS disease and late-onset GBS disease isolates for countries in the developed region and countries from all other regions.

Only seven studies, three for maternal colonisation and four for infant invasive disease, presented longitudinal data for serotype distribution changes over time [55], [61], [62], [63], [64], [65], [66]. In general, all studies described serotype variation over time but usually among the most common serotypes, for example in a South African study for infant invasive disease there were changes over a 10-year period with serotype Ia and III interchanging as the dominant serotypes for infant invasive disease [64]. Fig. 4 shows the distribution of GBS serotypes over the past decades according to the study periods of data collection (samples taken), for maternal colonisation, EOGBS and LOGBS.

***Maternal colonisation***: There was limited variation of serotype distribution through time in the developed region for serotypes III, V, Ia, Ib and II. However, there was a trend upwards for serotype IV, which showed a trend of increasing from 1% (95%CI: 0–3) pre-2001 (n = 28/1,440 isolates) to 3% (95%CI: 0–7) in 2001–2006 (n = 77/1,340 isolates), to 5% (95%CI: 3–7) in 2007–2012 (n = 197/3,556 isolates), to 7% (95%CI: 5–10) in 2013–2018 (n = 27/359). Non-typeable serotypes declined from 7% (95%CI: 4–12) in pre-2001, to 2% (95%CI: 0–5) in 2001–2006, to 1% (95%CI: 0–2) in 2007–2012, to 0% (95%CI: 0–2) in 2013–2018. In all other regions, serotypes Ia and Ib have reduced from 33% (95%CI: 22–44) in pre-2001 to 16% (95%CI: 10–24) in 2013–2018, and from 10% (95%CI: 6–15) in 2001–2006 to 5% (95%CI: 3–8) in 2013–2018, respectively. While serotypes III, V and VI-IX have been increasing (Fig. 4).

***Infant invasive disease (EOGBS and LOGBS)***: For EOGBS, in all other regions apart from developed region, there was a substantial increase in disease caused by serotype III in the period 2013–2018, from 46% (95%CI: 32–61) in pre-2001 to 69% (95%CI: 55–82) in 2013–2018. The rest of serotypes maintained a similar proportion through time or were fluctuating between time periods with no specific trend, for both the developed region and all other regions. For LOGBS serotypes fluctuate between time periods with no specific trends, always with serotype III predominating.

### 3.5 Sensitivity analysis

There were no differences between the serotype proportions of the main analysis (n = 198 studies) and the sensitivity analysis that excluded studies sampling or reporting only five or less serotypes (n = 161 studies) (Supplementary Fig. S18).

## 4 Discussion

This paper provides the most comprehensive worldwide review of GBS circulating serotypes, and the first systematic review, to our knowledge, on MLST data and proposed vaccine candidate proteins, which is timely given likely investments in GBS vaccines. A GBS maternal vaccine if effective would reduce invasive disease after birth (the target of IAP) but also reduce a major burden of stillbirths, maternal bacteremias, and LOGBS, where IAP is not expected to be effective. Additionally, most of the current burden is in LMIC (notably Africa and South Asia) where IAP is unlikely to be feasible to scale equitably. Our review considers all the relevant at-risk populations: in addition to the previous review of GBS serotypes for colonised pregnant women, early and late onset infant sepsis and meningitis, and maternal invasive disease, [5], [37], [38] we include stillbirths, which are often omitted, despite 2.6 million annually most of which are preventable [67]. We also included adults > 60 years.

Our top finding is that a hexavalent polysaccharide–protein conjugate vaccine (Ia, Ib, II, III, IV, V) has the potential to prevent up to 93% of worldwide maternal colonising isolates, 95% of maternal invasive GBS disease, 99% of GBS-associated stillbirth, and 99% of infant invasive GBS disease. Although evidence is still limited, a vaccine targeting maternal colonisation could provide additional protection against neonatal disease, and evaluation of this should be included in phase 2 studies. For maternal colonisation and maternal disease, the main serotypes across all regions were similar: Ia, III, and V. In EOGBS and LOGBS, serotype III dominated with 52% and 77%, respectively. An additional two serotypes (Ia, II) accounted for more than 15% of infant disease in the Americas, Europe, and East Asia, while serotypes Ia and V accounted for more than 20% of infant disease in Sub-Saharan Africa and Australia.

Elderly adult population was included for the first time, providing a novel picture of the circulating serotypes of GBS causing infection, and may inform potential benefit of including this at-risk group in use of a GBS vaccine. The commonest serotypes were V and Ia, accounting for nearly half of the disease in this populations, and fewer (14%) serotype III. Hence, a hexavalent vaccine could prevent up to 96% elderly invasive disease. We note that data were mainly from northern America and China.

MLST data were reported in only a quarter of the studies reviewed and even fewer (<5%) included WGS data. ST17 was more common in infant invasive disease, and although most ST17 isolates are serotype III, invasiveness of ST17 is independent of the capsular serotype [56]. It is clear that ST17 strains must be covered by any GBS vaccine candidate. Thus, sequence types, CC, and virulence factors associated with disease, add more targeted information rather than just the capsular polysaccharide. Additionally, analysis of the genotype through WGS may explore genetic recombination events such as capsular switching and mutations allowing GBS to become more virulent, which allows better observation of the potential bacterial population changes during and post-vaccine implementation. Serotype replacement and serotype switching following vaccination is a known limitation of the use of polysaccharide-protein conjugate vaccine. Evidence has suggested that GBS could undergo capsular switching through horizontal transfer of the capsular locus [22], [58], [68], notably within CC17 where serotype III strains switched to express serotype IV capsule. Lopes *et al*., also studied such an event in an ST1 strain switching from serotype V to Ib capsule [69]. Capsular switching in GBS may still be a rare occurrence, yet an introduction of capsular serotype-based vaccines could create a greater selection pressure leading to serotype replacement in disease as seen with childhood pneumococcal conjugate vaccine (PCV) immunisation [70], specifically an increase in non-vaccine serotypes.

Although our results suggest that serotypes (VI to IX) currently not part of a vaccine represent around 4% of maternal colonisation isolates worldwide, in south-eastern Asia, eastern Asia, and southern Asia, they represent 20%, 12% and 7%, respectively. In specific countries such as Japan, a higher prevalence of less common serotypes VI-IX has been reported [62], [71], [72], [73], [74], [75]. We calculated a pooled 39% (95%CI:25–53) of maternal colonisation isolates as serotypes VI-IX (from 5 studies, n = 728 isolates) [62], [71], [72], [73], [74]. However, we found these serotypes to be less frequently reported as causing invasive disease in infants, with 8% (95%CI:2–15) of EOGBS (4studies) [75], [76], [77], [78], and 1% (95%CI:0–3) of LOGBS (3studies) [75], [77], [78], while serotype III still predominates in these cases with 38% (95%CI:26–51) in EOGBS and 36% (95%CI:3–79) in LOGBS. Protein vaccines could theoretically reduce the risk of serotype replacement that theoretically exists for GBS capsular-polysaccharide based vaccines.

Protein antigen-based vaccines against GBS could provide an alternative to the multivalent polysaccharide-protein conjugate vaccines. Based on our findings, a protein-alum adjuvant vaccine, which contains the alp family surface proteins (alp1/epsilon, alp2/3, alpha C and Rib), has the potential to prevent up to 87% of maternal colonisation, 99% of adult invasive disease and 93% infant invasive disease (percentages of isolates with at least one of the four protein targets). In addition to the alp protein-based vaccines, the pilus proteins have been proposed as potential vaccine candidates, albeit with conflicting data on whether it has potential as a vaccine candidate [31], [79]. Our findings require confirmation from ongoing seroepidemiological studies.

A strength of our review is containing serotype data for many countries (n = 62), including published and unpublished data, with notably increasing data from China, South Africa, and Iran. Additionally, the large number of studies (n = 198) and isolates (n = 29,247) spanning 20-years allowed us to compare serotype distribution by developed and other regions, to try to mitigate potential bias due to specific geographical mix of countries in each time period, whilst examining time trends.

Time trends however should be interpreted carefully as it is uncertain if the reported changes in proportions are due to one serotype becoming less/more common or if the changes are relative to other serotype changes or capture, since differences in proportions are due to different denominators. Longitudinal studies in a single population have shown fluctuations but mainly between dominant serotypes [64]. Changes in serotyping methods, such as an increase in the use of molecular methods (PCR and sequencing) with time, could also bias the trend analysis. PCR and sequencing use genetic targets to identify serotypes, hence GBS that either do not have a capsule or have a poorly expressed capsule can now be serotyped. Studies that used molecular methods had lower percentage of non-typeable strains (supplementary Table S7). Methodological changes could explain apparent reduction of non-typeable isolates over time, as PCR and sequencing use increased in the period 2013–2018 compared to previous periods. Additionally, time period classification was by years of data collection, but the literature search for maternal colonisation studies from developed countries and for infant disease studies was limited to publications after the year 2000, for reason previously explained [5], [37], which could introduce some bias for results pre-2001 for developed countries.

Another important limitation are data gaps for some regions. Although we were able to increase the geographical representation compared to our last review [5], [37], [36], there is still a paucity of data especially on invasive disease from southern Asia. However, data from maternal colonisation isolates from Asia can give some insight into circulating GBS serotypes, although these are not necessarily the same dominant serotypes as those in invasive infant disease [80]. There are important data gaps for some population groups, notably stillbirths, maternal invasive disease and adults > 60 yrs. Most data came from developed regions. For example, results for maternal invasive disease were mainly from the USA (4/6 studies), where the most common maternal colonising serotype is Ia. Protein expression was limited to few studies and these may therefore not be representative of the global situation.

## 5 Conclusion

GBS contributes a large burden of neonatal and infant disease, particularly in low- and middle-income countries (LMIC), yet GBS disease also has an under-recognised burden among pregnant/postnatal women and stillbirths, as well as the elderly. Access to IAP is low in LMIC, where maternal vaccination strategies may be a high impact, is a more feasible alternative [11]. More studies on GBS strains to inform vaccine developers are needed to fill in the data gaps, especially LMIC and for neglected, populations such as stillbirths. MLST/WGS data help inform which ST/CC and proteins are causing disease and add value beyond considering the serotype alone. Regular systematic compilation of data on GBS cases, serotypes and sequence types are needed, ideally embedded in routine systems for perinatal outcomes. These data are important to guide vaccine development, but improved routine data monitoring post-vaccine licensure will be key to ensure progress for the poorest, who are currently most likely to be left uncounted.

## Summary

Group B *Streptococcus* (GBS) maternal carriage affects an estimated 21 million women worldwide with several GBS vaccines in development. We summarise data regarding serotypes, sequence types and virulence markers to inform vaccine design and implementation, considering at-risk populations, geographical variations and time trends.

## Funding

This work was funded only by Bill & Melinda Gates Foundation from the United Stated of America and the grant number is OPP1180644, grant given to the London School of Hygiene and Tropical Medicine.

## Declaration of Competing Interest

The authors declare that they have no known competing financial interests or personal relationships that could have appeared to influence the work reported in this paper.
