# Supplementary material for: Systematic review of Group B Streptococcal capsular types, sequence types and surface proteins as potential vaccine candidates
Source: Vaccine. 2020 Oct 7;38(43):6682–94. doi: 10.1016/j.vaccine.2020.08.052 (PMC7526974; doi:10.1016/j.vaccine.2020.08.052)
Supplement: Supplementary data 1 [file mmc1.docx]

# **Systematic review of Group B Streptococcal capsular types, sequence types and surface proteins as potential vaccine candidates**

#

# **Supplementary information**

Contents

[**Systematic review of Group B Streptococcal capsular types, sequence types and surface proteins as potential vaccine candidates** 1](#_Toc48828588)

[**Supplementary information** 1](#_Toc48828589)

[Supplementary Table S1: definitions 4](#_Toc48828590)

[Supplementary Table S2: search terms 5](#_Toc48828591)

[Supplementary Table S3: inclusion and exclusion criteria 7](#_Toc48828592)

[Supplementary Table S4: Study characteristics and data abstracted for serotype papers 8](#_Toc48828593)

[Supplementary Table S5: Study characteristics and data abstracted for MLST and virulence proteins papers 24](#_Toc48828594)

[Supplementary Table S6: Studies with number of strains ST17 differentiated by EOGBS and LOGBS. 32](#_Toc48828595)

[Supplementary Table S7: number of non-typeable samples (NT) and proportion meta-analysis for each serotyping method 33](#_Toc48828596)

[Supplementary Figure S1: Geographic distribution of available data for group B *Streptococcus* (GBS) serotypes for maternal colonisation. 34](#_Toc48828597)

[Supplementary Figure S2: Geographic distribution of available data for group B *Streptococcus* (GBS) serotypes for infant GBS disease. 35](#_Toc48828598)

[Supplementary Figure S3: Geographic distribution of available data for group B *Streptococcus* (GBS) serotypes for maternal invasive GBS disease. 36](#_Toc48828599)

[Supplementary Figure S4: Geographic distribution of available data for group B *Streptococcus* (GBS) serotypes for GBS-associated stillbirths. 37](#_Toc48828600)

[Supplementary Figure S5: Geographic distribution of available data for group B *Streptococcus* (GBS) serotypes for GBS invasive disease in elderly population 38](#_Toc48828601)

[Supplementary Figure S6: Geographic distribution of available data for group B *Streptococcus* (GBS) from MLST and virulence genes search 39](#_Toc48828602)

[Supplementary Figure S7: Distribution of group B Streptococcus (GBS) serotypes for maternal colonisation isolates in African subregions (adjusted proportions). 40](#_Toc48828603)

[Supplementary Figure S8: Distribution of group B Streptococcus (GBS) serotypes for maternal colonisation isolates in North America (United States of America and Canada) and Europe (adjusted proportions). 41](#_Toc48828604)

[Supplementary Figure S9: Distribution of group B Streptococcus (GBS) serotypes within isolates from clonal complexes 1, 23, 19, 10, 17 and 12 for maternal colonisation and infant colonisation. 42](#_Toc48828605)

[Supplementary Figure S10: Distribution of group B Streptococcus (GBS) serotypes within isolates with alp1/epsilon, alp2/3, rib, alpha C or none surface protein genes for maternal colonisation and infant colonisation. 43](#_Toc48828606)

[Supplementary Figure S11: Distribution of group B Streptococcus (GBS) serotypes within isolates with pilus island 2a, PI-2b and combinations with PI-1 for maternal colonisation. 44](#_Toc48828607)

[Supplementary Figure S12: Distribution of group B Streptococcus (GBS) serotypes for maternal invasive GBS disease isolates by regions (adjusted proportions). 45](#_Toc48828608)

[Supplementary Figure S13: Distribution of group B Streptococcus (GBS) serotypes within isolates from clonal complexes 1, 23, 19, 10, 17 and 12 for maternal invasive, infant invasive and adult invasive. 46](#_Toc48828609)

[Supplementary Figure S14: Distribution of group B Streptococcus (GBS) serotypes for infant invasive GBS disease and stillbirth isolates in African subregions (adjusted proportions). 48](#_Toc48828610)

[Supplementary Figure S15: Distribution of group B Streptococcus (GBS) serotypes for infant invasive GBS disease and stillbirths isolates in North America (United States of America and Canada) and Europe (adjusted proportions). 49](#_Toc48828611)

[Supplementary Figure S16: Distribution of group B Streptococcus (GBS) serotypes within isolates with alp1/epsilon, alp2/3, rib, alpha C or none surface protein genes for infant invasive disease and adult invasive disease. 50](#_Toc48828612)

[Supplementary Figure S17: Distribution of group B Streptococcus (GBS) serotypes within isolates with pilus island 2a, PI-2b and combinations with PI-1 for infant invasive disease and adult invasive disease. 51](#_Toc48828613)

[Supplementary Figure S18: Plot comparing meta-analysis results for main analysis with all studies (n1=studies/samples) and sensitivity analysis (n2=studies/samples) for maternal colonisation (A), infant invasive disease (B), and invasive disease in elderly (C) 52](#_Toc48828614)

[References 53](#_Toc48828615)

## Supplementary Table S1: definitions

| **Term** | **Definition** |
| --- | --- |
| Maternal GBS colonisation | GBS isolated from vaginal, cervical, and/or rectal swabs from pregnant women |
| Invasive GBS disease | Isolation of GBS from a normally sterile site, such as blood or cerebrospinal fluid (CSF) |
| Maternal invasive GBS disease | Invasive GBS disease in pregnant or postpartum women (up to 42 days postpartum) with symptoms of sepsis, minimum of a fever |
| Stillbirth | - World Health Organization definition for international comparison and reporting: birth of a fetus with no signs of life at ≥28 weeks’ gestation or weighing 1000 g - International Classification of Disease (ICD) definition: birth of a fetus with no signs of life at 22 weeks or more gestation or weighing >500g |
| GBS-associated stillbirth | Microbiological evidence of invasive GBS disease |
| Early-onset GBS disease (EOGBS) | Invasive GBS disease in infants during 0-6 days after birth |
| Late-onset GBS disease (LOGBS) | Invasive GBS disease with onset during 7 to 89 days after birth |
| GBS invasive disease in the elderly | Invasive GBS disease after 60 years of age |

## Supplementary Table S2: search terms

| **Outcome/target** | **Search terms** | **Search limits** |
| --- | --- | --- |
| Maternal colonisation | Matern* OR pregnan* OR Antenatal OR Antepartum OR Vagin* OR Recto-Vagin* OR vagino-rectal OR rectovaginal OR Obstetric*OR Pregnancy [MeSH terms] OR Vagina [MeSH] AND Streptococcus agalactiae OR group b streptococc* OR Streptococc* group B OR Streptococcus agalactiae [MeSH terms] AND Serotyp* | Current literature search: Limited to humans and publications from 1 January 2017 to March 2019  Previous literature search(1): For developed regions data only included if published after year 2000, unless only data before this period |
| Maternal GBS | Maternal OR Mother OR Puerperal OR Parturient OR Antepartum OR Intrapartum OR Peripartum OR Postpartum OR Pregnan$ OR Pregnancy [MeSH Terms] AND Sepsis OR Septic OR Bacteraemia OR Bacteremia OR Sepsis [MeSH Terms] AND (“Streptococcus” [All Fields] OR “Streptococcal” [All Fields] OR “Streptococci” [All Fields]) AND ((“Group” AND “B”) or “Agalactiae”) OR “Streptococcus Agalactiae” [MeSH Terms] AND serotype* | Limited to humans |
| Infant | Additional search for reports with serotype data using search terms: “Streptococcus agalactiae serotype” [MeSH terms] OR “Streptococcus Group B serotype” OR “Group B streptococcal serotype” AND “infant” AND serotype* | Current literature search: Limited to humans and publications from 1 January 2017 to March 2019  Previous literature search: publications from 1 January 2000 to 31 January 2017 |
| Stillbirth | Stillbirth [MeSH Term] OR Fetal Death OR Perinatal Death OR Perinatal Mortality AND Streptococcus OR Streptococcal OR Streptococci AND (Group AND B) or agalactiae OR Streptococcus agalactiae [MeSH Terms] AND serotype* | Limited to humans |
| Elderly | (elder* or adult* or non-pregnant* or nonpregnant* OR non-pregnant OR 65 year* OR adult [MeSH Terms]) AND (((Streptococcus or Streptococcal or Streptococci) AND ((Group and B) or agalactiae)) OR Streptococcus agalactiae [MeSH Terms]) AND serotyp* | Limited to humans |
| Multi locus sequence typing | Group B Streptococcus [MeSH] OR Streptococcus agalactiae [MeSH] AND Whole Genome OR WGS OR Sequenc* OR Next Generation OR MLST OR epidemiology OR population structure AND (Transmi* OR Spread* OR Maternal Colonisation OR Coloni* OR Vertical Transmission OR Infant OR Neonatal OR Maternal OR sepsis OR adult OR stillbirth | Limited to humans, search limited from 1 January 2000 to February 2020 |
| Virulence proteins | Group B Streptococcus [MeSH] OR Streptococcus agalactiae [MeSH] AND alp1 OR alp2 OR alp2/3 OR alp4 OR rib OR pilus island OR bca OR epsilon OR alpha c | Limited to humans, search limited from 1 January 2000 to February 2020 |

## Supplementary Table S3: inclusion and exclusion criteria

| **Inclusion criteria** | **Exclusion criteria** |
| --- | --- |
| Studies with population and study design described, reporting prevalence of serotypes of colonizing isolates | Excluded studies involving nonpregnant women where results for pregnant women could not be separately extracted |
| Data on serotypes included if clearly identified as colonizing pregnant women vaginally or rectally, and were not from invasive disease. | Excluded if pregnancy serotypes not reported separately |
| Data of cohort of women or pooled laboratory samples. | Studies investigating only specific serotypes or virulence factors |
| Any observational studies reporting serotype data of invasive GBS disease in pregnant women or women up to 42 days postpartum | Excluded studies where cultures were only taken from potentially nonsterile or contaminated sites |
| Studies with GBS invasive disease in infants aged 0–89 days at onset of infection episode, with clinical specimens obtained from a sterile site | Excluded studies if serotypes for the specific age group not clearly defined |
| Studies including all stillbirths in a facility, or occurring in a geographical location in a specified time period with stillbirths defined WHO definition >28 weeks gestation or >22 weeks gestation in developed countries, with a positive sterile site culture (blood, cerebrospinal fluid, joint fluid, pleural or peritoneal fluid)  Studies with GBS invasive disease in nonpregnant adults aged 60 or more years, with clinical specimens obtained from a sterile site |  |

## Supplementary Table S4: Study characteristics and data abstracted for serotype papers

| **Outcome** | **Author** | **Region** | **Country** | **Year Published** | **Group year data collected** | **No specimens taken for serotype** | **Ia** | **Ib** | **II** | **III** | **IV** | **V** | **VI** | **VII** | **VIII** | **IX** | **NT** |
| --- | --- | --- | --- | --- | --- | --- | --- | --- | --- | --- | --- | --- | --- | --- | --- | --- | --- |
| Maternal colonisation | Pinto(2) | Developed | Portugal | 2018 | 2013-2018 | 67 | 13 | 12 | 7 | 15 | 5 | 12 | 1 | 0 | 0 | 0 | 2 |
| Maternal colonisation | Creti(3) | Developed | Italy | 2017 | 2007-2012 | 320 | 57 | 19 | 40 | 122 | 8 | 66 | 1 | 0 | 0 | 5 | 2 |
| Maternal colonisation | Kardos(4) | Developed | Hungary | 2017 | 2013-2018 | 95 | 14 | 5 | 9 | 27 | 6 | 34 | 0 | 0 | 0 | 0 | 0 |
| Maternal colonisation | Teatero(5) | Developed | Canada | 2017 | 2013-2018 | 102 | 24 | 12 | 13 | 26 | 6 | 20 | 1 | 0 | 0 | 0 | 0 |
| Maternal colonisation | Lopez(6) | Developed | Spain | (2017) | 2013-2018 | 95 | 17 | 4 | 30 | 25 | 10 | 9 | 0 | 0 | 0 | 0 | 0 |
| Maternal colonisation | Rojo-Bezares(7) | Developed | Spain | 2016 | 2007-2012 | 65 | 8 | 8 | 9 | 22 | 2 | 16 | 0 | 0 | 0 | 0 | 0 |
| Maternal colonisation | Ko(8) | Developed | Australia | 2015 | 2007-2012 | 408 | 107 | 31 | 40 | 142 | 5 | 68 | 3 | 3 | 0 | 0 | 9 |
| Maternal colonisation | Kunze(9) | Developed | Germany | 2015 | 2007-2012 | 165 | 40 | 12 | 25 | 46 | 8 | 32 | 1 | 1 | 0 | 0 | 0 |
| Maternal colonisation | Morozumi(10) | Developed | Japan | 2015 | 2007-2012 | 154 | 24 | 32 | 7 | 26 | 5 | 27 | 19 | 0 | 13 |  | 1 |
| Maternal colonisation | Brigtsen(11) | Developed | Norway | 2015 | 2007-2012 | 426 | 67 | 42 | 59 | 106 | 60 | 72 | 2 | 0 | 3 | 12 | 3 |
| Maternal colonisation | Liebana-Martos(12) | Developed | Spain | 2015 | 2007-2012 | 188 | 42 | 6 | 31 | 51 | 8 | 35 | 0 | 1 | 0 | 9 | 5 |
| Maternal colonisation | Meehan(13) | Developed | Ireland | 2014 | 2007-2012 | 18 | 4 | 1 | 3 | 7 | 0 | 2 | 0 | 1 | 0 | 0 | 0 |
| Maternal colonisation | Romanik(14) | Developed | Poland | 2014 |  | 23 | 10 | 0 | 1 | 5 | 0 | 7 | 0 | 0 | 0 |  | 0 |
| Maternal colonisation | Frohlicher(15) | Developed | Switzerland | 2014 | 2007-2012 | 364 | 70 | 25 | 38 | 107 | 14 | 93 | 1 | 2 | 1 | 13 | 0 |
| Maternal colonisation | Kimura(16) | Developed | Japan | 2013 | 2007-2012 | 141 | 10 | 26 | 18 | 17 | 2 | 29 | 22 | 1 | 15 | 0 | 1 |
| Maternal colonisation | Barcaite(17) | Developed | Lithuania | 2012 | 2007-2012 | 148 | 44 | 7 | 16 | 51 | 13 | 11 | 3 | 0 | 0 |  | 3 |
| Maternal colonisation | Brzychczy-wloch(18) | Developed | Poland | 2012 | 2007-2012 | 353 | 71 | 28 | 53 | 123 | 17 | 61 | 0 | 0 | 0 |  | 0 |
| Maternal colonisation | Kunze(19) | Developed | Germany | 2011 | 2001-2006 | 155 | 25 | 30 | 19 | 43 | 9 | 23 |  |  |  |  | 6 |
| Maternal colonisation | Wakimoto(20) | Developed | Japan | 2011 | 2001-2006 | 198 | 26 | 33 | 13 | 15 | 1 | 17 | 54 | 0 | 37 |  | 2 |
| Maternal colonisation | Cristea(21) | Developed | Romania | 2011 | 2007-2012 | 257 | 51 | 12 | 29 | 84 | 7 | 60 |  |  |  |  | 14 |
| Maternal colonisation | Brzychczy-wloch(22) | Developed | Poland | 2010 | 2007-2012 | 100 | 25 | 7 | 14 | 29 | 7 | 18 | 0 | 0 | 0 |  | 0 |
| Maternal colonisation | Gonzalez(23) | Developed | Spain | 2010 |  | 150 | 38 | 0 | 15 | 50 | 11 | 21 | 0 | 3 | 0 | 0 | 10 |
| Maternal colonisation | Liebana(24) | Developed | Spain | 2010 | 2007-2012 | 188 | 38 | 8 | 23 | 53 | 11 | 31 | 0 | 0 | 2 | 10 | 12 |
| Maternal colonisation | El Aila(25) | Developed | Belgium | 2009 | 2007-2012 | 122 | 13 | 13 | 13 | 25 | 15 | 25 |  |  |  |  | 18 |
| Maternal colonisation | Van Der Mee-Marquet(26) | Developed | France | 2009 |  | 39 | 10 | 3 | 3 | 16 | 0 | 7 | 0 | 0 | 0 | 0 | 0 |
| Maternal colonisation | Van Elzakker(27) | Developed | Netherlands | 2009 | pre 2000-2000 | 92 | 24 | 6 | 12 | 20 | 7 | 14 | 7 | 0 | 0 |  | 0 |
| Maternal colonisation | Wolski(28) | Developed | Poland | 2009 |  | 100 |  |  | 7 | 32 |  |  |  |  |  |  | 9 |
| Maternal colonisation | Usein(29) | Developed | Romania | 2009 | 2007-2012 | 100 | 19 | 3 | 26 | 26 | 9 | 17 | 0 | 0 | 0 |  | 0 |
| Maternal colonisation | Usein(30) | Developed | Romania | 2009 | 2007-2012 | 39 | 6 | 6 | 7 | 11 | 6 | 3 |  |  |  |  | 0 |
| Maternal colonisation | Savoia(31) | Developed | Italy | 2008 | 2001-2006 | 73 | 16 | 5 | 4 | 23 | 6 | 19 |  |  |  |  | 0 |
| Maternal colonisation | Hakansson(32) | Developed | Sweden | 2008 | 2001-2006 | 356 | 39 | 46 | 57 | 85 | 53 | 68 | 2 | 3 | 0 | 0 | 3 |
| Maternal colonisation | Martins(33) | Developed | Portugal | 2007 | 2001-2006 | 269 | 42 | 14 | 46 | 59 | 6 | 59 | 0 | 5 | 0 |  | 38 |
| Maternal colonisation | Taylor(34) | Developed | Australia | 2006 | 2001-2006 | 19 | 5 | 2 | 0 | 9 | 0 | 0 | 0 | 1 | 0 | 0 | 2 |
| Maternal colonisation | Lamy(35) | Developed | France | 2006 | pre 2000-2000 | 42 | 5 | 7 | 3 | 22 | 0 | 2 | 0 | 0 | 0 | 0 | 3 |
| Maternal colonisation | Marchaim(36) | Developed | Israel | 2006 | pre 2000-2000 | 72 | 10 | 8 | 22 | 15 | 2 | 10 | 1 | 0 | 0 | 0 | 4 |
| Maternal colonisation | Jones(37) | Developed | United Kingdom | 2006 | 2001-2006 | 159 | 41 | 25 | 15 | 42 |  | 30 |  |  |  |  |  |
| Maternal colonisation | Bisharat(38) | Developed | Israel | 2005 | pre 2000-2000 | 104 | 12 | 9 | 23 | 26 | 0 | 18 | 0 | 0 | 0 |  | 16 |
| Maternal colonisation | Motlova(39) | Developed | Czech Republic | 2004 | 2001-2006 | 172 | 38 | 14 | 0 | 58 | 0 | 24 | 5 | 0 | 0 |  | 0 |
| Maternal colonisation | Whitney(40) | Developed | Ireland | 2004 |  | 20 | 7 | 1 | 1 | 6 | 0 | 4 | 0 | 0 | 0 | 0 | 1 |
| Maternal colonisation | Von Both(41) | Developed | Germany | 2003 | pre 2000-2000 | 146 | 26 | 11 | 22 | 43 | 4 | 19 | 0 | 0 | 0 | 0 | 21 |
| Maternal colonisation | Tsolia(42) | Developed | Greece | 2003 | 2001-2006 | 67 | 13 | 8 | 18 | 15 | 2 | 6 | 2 | 2 | 1 |  | 0 |
| Maternal colonisation | Dore(43) | Developed | Ireland | 2003 | pre 2000-2000 | 87 | 15 | 8 | 13 | 30 | 2 | 14 | 0 | 0 | 0 |  | 5 |
| Maternal colonisation | Perez-Ruiz(44) | Developed | Spain | 2003 | 2001-2006 | 31 | 9 | 4 | 4 | 12 | 0 | 2 |  |  | 0 |  | 0 |
| Maternal colonisation | Croak(45) | Developed | United States of America | 2003 | pre 2000-2000 | 145 | 25 | 11 | 17 | 24 | 5 | 46 | 1 |  |  |  | 16 |
| Maternal colonisation | Terakubo(46) | Developed | Japan | 2002 | pre 2000-2000 | 187 | 16 | 12 | 3 | 19 | 0 | 16 | 46 | 0 | 59 |  | 16 |
| Maternal colonisation | Matsubara(47) | Developed | Japan | 2002 | pre 2000-2000 | 48 | 4 | 6 | 4 | 5 | 0 | 3 | 9 | 0 | 13 | 0 | 4 |
| Maternal colonisation | Grimwood(48) | Developed | New Zealand | 2002 | pre 2000-2000 | 52 | 11 | 10 | 3 | 15 | 0 | 10 | 1 | 0 | 1 |  | 1 |
| Maternal colonisation | Davies(49) | Developed | Canada | 2001 | pre 2000-2000 | 118 | 24 | 10 | 10 | 18 | 0 | 33 | 0 | 0 | 0 |  | 23 |
| Maternal colonisation | Davies(49) | Developed | Canada | 2001 | pre 2000-2000 | 233 | 53 | 25 | 30 | 48 | 5 | 45 | 0 | 0 | 1 |  | 26 |
| Maternal colonisation | Berg(50) | Developed | Sweden | 2000 | pre 2000-2000 | 114 | 15 | 15 | 13 | 36 | 3 | 25 | 0 | 0 | 0 |  | 7 |
| Maternal colonisation | Campbell(51) | Developed | United States of America | 2000 | pre 2000-2000 | 856 | 225 | 64 | 155 | 183 |  | 179 | 2 | 0 | 0 |  | 11 |
| Maternal colonisation | Lee(52) | Eastern Asia | South Korea | 2019 | 2013-2018 | 19 | 0 | 4 | 2 | 8 | 1 | 3 | 1 | 0 | 0 | 0 | 0 |
| Maternal colonisation | Wang(53) | Eastern Asia | China | 2018 | 2013-2018 | 104 | 23 | 8 | 0 | 51 | 2 | 19 | 1 | 0 | 0 | 0 | 0 |
| Maternal colonisation | Ji(54) | Eastern Asia | China | 2017 | 2007-2012 | 153 | 27 | 20 | 0 | 84 | 0 | 10 | 2 | 0 | 1 | 0 | 9 |
| Maternal colonisation | Yan(55) | Eastern Asia | China | 2016 | 2013-2018 | 231 | 52 | 24 | 12 | 83 | 0 | 49 | 3 | 0 | 2 | 0 | 6 |
| Maternal colonisation | Wang(56) | Eastern Asia | China | 2015 | 2013-2018 | 56 | 10 | 9 | 3 | 18 | 0 | 8 | 0 | 0 | 0 | 0 | 8 |
| Maternal colonisation | Lu(57) | Eastern Asia | China | 2015 | 2007-2012 | 160 | 33 | 18 | 10 | 72 | 0 | 23 | 1 | 0 | 0 | 0 | 3 |
| Maternal colonisation | Lu(58) | Eastern Asia | China | 2014 | 2007-2012 | 201 | 43 | 24 | 14 | 84 | 1 | 30 | 3 | 0 | 1 |  | 1 |
| Maternal colonisation | Lee(59) | Eastern Asia | South Korea | 2010 | 2007-2012 | 318 | 35 | 37 | 19 | 111 | 0 | 87 | 27 | 2 | 0 |  | 0 |
| Maternal colonisation | Seo(60) | Eastern Asia | South Korea | 2010 | 2007-2012 | 145 | 26 | 16 | 8 | 51 | 0 | 35 | 4 | 1 | 4 | 0 | 0 |
| Maternal colonisation | Lee(61) | Eastern Asia | South Korea | 2010 | 2007-2012 | 315 | 38 | 30 | 14 | 138 | 0 | 64 | 14 | 3 | 9 |  | 5 |
| Maternal colonisation | Hong(62) | Eastern Asia | South Korea | 2010 | 2007-2012 | 177 | 23 | 12 | 10 | 63 | 4 | 43 |  |  |  |  | 22 |
| Maternal colonisation | Oh(63) | Eastern Asia | South Korea | 2009 | 2001-2006 | 42 | 11 | 2 | 2 | 12 | 0 | 11 | 4 | 0 | 0 | 0 | 0 |
| Maternal colonisation | Van Elzakker(27) | Eastern Asia | Taiwan | 2009 | 2001-2006 | 58 | 13 | 5 | 2 | 19 | 0 | 15 | 2 | 0 | 0 |  | 0 |
| Maternal colonisation | Shen(64) | Eastern Asia | China | 2000 | pre 2000-2000 | 155 | 39 | 4 | 55 | 40 | 0 | 6 |  |  |  |  | 11 |
| Maternal colonisation | Shen(65) | Eastern Asia | China | 1998 | pre 2000-2000 | 22 | 5 | 3 | 7 | 4 | 1 | 0 |  |  |  |  | 2 |
| Maternal colonisation | Uh(66) | Eastern Asia | South Korea | 1997 | pre 2000-2000 | 29 | 7 | 14 | 0 | 6 | 0 | 0 |  |  |  |  | 2 |
| Maternal colonisation | Botelho(67) | Latin America and the Caribbean | Brazil | 2018 | 2007-2012 | 689 | 257 | 77 | 137 | 47 | 24 | 63 | 0 | 0 | 0 |  | 84 |
| Maternal colonisation | Oviedo(68) | Latin America and the Caribbean | Argentina | 2013 | 2007-2012 | 112 | 45 | 10 | 11 | 24 | 0 | 13 | 0 | 0 | 0 | 5 | 4 |
| Maternal colonisation | Soares(69) | Latin America and the Caribbean | Brazil | 2013 |  | 39 | 4 | 2 | 3 | 15 | 0 | 10 |  |  |  |  | 5 |
| Maternal colonisation | Palmeiro(70) | Latin America and the Caribbean | Brazil | 2010 | 2007-2012 | 30 | 12 | 5 | 6 | 1 | 1 | 2 | 0 | 0 | 0 |  | 3 |
| Maternal colonisation | Simoes(71) | Latin America and the Caribbean | Brazil | 2007 | 2001-2006 | 47 | 9 | 11 | 9 | 3 | 3 | 4 |  |  |  |  | 8 |
| Maternal colonisation | Gonzalez(72) | Latin America and the Caribbean | Mexico | 2004 | pre 2000-2000 | 31 |  |  | 5 | 2 | 0 | 0 |  |  |  |  | 0 |
| Maternal colonisation | Gonzalez - Pedraza(73) | Latin America and the Caribbean | Mexico | 2002 | pre 2000-2000 | 101 | 62 | 0 | 26 | 13 | 0 | 0 |  |  |  |  | 0 |
| Maternal colonisation | Ocampo-Torres(74) | Latin America and the Caribbean | Mexico | 2000 | pre 2000-2000 | 78 |  |  | 15 | 10 | 0 | 0 |  |  |  |  | 0 |
| Maternal colonisation | Solorzano-Santos(75) | Latin America and the Caribbean | Mexico | 1989 | pre 2000-2000 | 33 | 19 | 3 | 4 | 1 | 0 |  |  |  |  |  | 6 |
| Maternal colonisation | Benchetrit(76) | Latin America and the Caribbean | Brazil | 1982 | pre 2000-2000 | 31 | 10 | 10 | 5 | 3 | 0 | 0 |  |  |  |  | 3 |
| Maternal colonisation | Moraleda(77) | Northern Africa | Morocco | 2018 | 2013-2018 | 67 | 6 | 0 | 17 | 12 | 5 | 24 | 0 | 0 | 0 | 3 | 0 |
| Maternal colonisation | Bergal(78) | Northern Africa | Algeria | 2015 | 2007-2012 | 44 | 2 | 0 | 11 | 10 | 0 | 21 | 0 | 0 | 0 | 0 | 0 |
| Maternal colonisation | Benbachir(79) | Northern Africa | Morocco | 1983 | pre 2000-2000 | 15 | 6 | 1 | 3 | 4 | 0 | 0 |  |  |  |  | 1 |
| Maternal colonisation | Suhaimi(80) | South-eastern Asia | Malaysia | 2017 | 2013-2018 | 12 | 7 | 0 | 0 | 2 | 0 | 2 | 0 | 0 | 1 | 0 | 0 |
| Maternal colonisation | Eskandarian(81) | South-eastern Asia | Malaysia | 2015 | 2007-2012 | 49 | 6 | 0 | 5 | 2 | 0 | 9 | 18 | 9 | 0 |  | 0 |
| Maternal colonisation | Turner(82) | South-eastern Asia | Myanmar | 2012 | 2007-2012 | 66 | 11 | 1 | 16 | 8 | 4 | 8 | 11 | 4 | 0 | 0 | 3 |
| Maternal colonisation | Dhanoa(83) | South-eastern Asia | Malaysia | 2010 | 2007-2012 | 200 | 23 | 3 | 11 | 24 | 20 | 38 | 34 | 10 | 3 |  | 34 |
| Maternal colonisation | Whitney(40) | South-eastern Asia | Myanmar | 2004 |  | 14 | 2 | 0 | 5 | 0 | 0 | 5 | 0 | 0 | 0 | 0 | 2 |
| Maternal colonisation | Whitney(40) | South-eastern Asia | Philippines | 2004 |  | 15 | 1 | 1 | 4 | 5 | 0 | 2 | 0 | 0 | 1 | 0 | 1 |
| Maternal colonisation | Whitney(40) | South-eastern Asia | Thailand | 2004 | pre 2000-2000 | 24 | 5 | 0 | 3 | 3 | 0 | 12 | 0 | 0 | 0 | 0 | 1 |
| Maternal colonisation | Whitney(40) | South-eastern Asia | Thailand | 2004 |  | 28 | 4 | 1 | 4 | 6 | 0 | 2 | 0 | 8 | 0 | 0 | 2 |
| Maternal colonisation | Kumar(84) | Southern Asia | India | unpublished | 2001-2006 | 54 | 29 | 12 | 0 | 3 | 0 | 3 | 0 | 0 | 0 | 0 | 2 |
| Maternal colonisation | Saha(85) | Southern Asia | Bangladesh | 2016 | 2013-2018 | 172 | 69 | 2 | 24 | 20 | 1 | 40 | 12 | 0 | 3 | 1 | 0 |
| Maternal colonisation | Chaudhary(86) | Southern Asia | India | 2017 | 2013-2018 | 45 | 6 | 2 | 9 | 10 | 0 | 9 | 0 | 3 | 0 | 0 | 6 |
| Maternal colonisation | Chan(87) | Southern Asia | Bangladesh | 2013 | 2007-2012 | 94 | 23 | 3 | 7 | 9 | 0 | 28 | 12 | 5 | 0 | 3 | 4 |
| Maternal colonisation | Mani(88) | Southern Asia | India | 1984 | pre 2000-2000 | 19 | 4 | 6 | 8 | 1 | 0 | 0 | 0 | 0 | 0 | 0 | 0 |
| Maternal colonisation | Africa(89) | Sub-Saharan Africa | South Africa | 2018 |  | 57 | 1 | 0 | 1 | 12 | 1 | 38 | 0 | 0 | 0 | 1 | 3 |
| Maternal colonisation | Medugu(90) | Sub-Saharan Africa | Nigeria | 2017 | 2013-2018 | 171 | 22 | 3 | 39 | 18 | 3 | 74 |  |  |  |  | 7 |
| Maternal colonisation | Slotved(91) | Sub-Saharan Africa | Ghana | 2017 | 2013-2018 | 108 | 2 | 0 | 1 | 6 | 9 | 7 | 0 | 44 | 7 | 32 | 0 |
| Maternal colonisation | Le Doare(92) | Sub-Saharan Africa | Gambia | 2016 | 2013-2018 | 237 | 20 | 16 | 39 | 24 |  | 130 |  |  |  |  | 0 |
| Maternal colonisation | Seale(93) | Sub-Saharan Africa | Kenya | 2016 | 2007-2012 | 915 | 194 | 114 | 80 | 350 | 18 | 156 | 0 | 3 | 0 | 0 | 0 |
| Maternal colonisation | Madrid(94) | Sub-Saharan Africa | Mozambique | 2016 | 2013-2018 | 64 | 9 | 6 | 3 | 6 | 3 | 20 | 0 | 0 | 0 | 0 | 17 |
| Maternal colonisation | Dangor(95) | Sub-Saharan Africa | South Africa | 2016 | 2013-2018 | 72 | 39 |  |  | 14 |  | 12 |  |  |  |  |  |
| Maternal colonisation | Belard(96) | Sub-Saharan Africa | Gabon | 2015 | 2007-2012 | 109 | 14 | 25 | 7 | 30 | 0 | 33 | 0 | 0 | 0 | 0 | 0 |
| Maternal colonisation | Vinnemeier(97) | Sub-Saharan Africa | Ghana | 2015 | 2013-2018 | 24 | 2 | 5 | 3 | 7 |  | 5 |  |  |  |  | 2 |
| Maternal colonisation | Vinnemeier(97) | Sub-Saharan Africa | Ghana | 2015 | 2013-2018 | 72 | 25 | 3 | 6 | 14 |  | 21 |  |  |  |  | 3 |
| Maternal colonisation | Chukwu(98) | Sub-Saharan Africa | South Africa | 2015 |  | 128 | 33 | 11 | 20 | 38 | 11 | 14 | 0 | 0 | 0 | 0 | 1 |
| Maternal colonisation | Kwatra(99) | Sub-Saharan Africa | South Africa | 2014 | 2007-2012 | 152 | 55 | 7 | 11 | 53 | 3 | 18 | 0 | 0 | 0 | 5 | 0 |
| Maternal colonisation | Gray(100) | Sub-Saharan Africa | Malawi | 2011 | 2007-2012 | 390 | 71 | 24 | 40 | 152 | 1 | 93 | 1 | 0 | 2 | 0 | 6 |
| Maternal colonisation | Madzivhandila(101) | Sub-Saharan Africa | South Africa | 2011 | 2001-2006 | 541 | 163 | 36 | 61 | 202 | 20 | 55 | 0 | 0 | 0 | 0 | 4 |
| Maternal colonisation | Brochet(102) | Sub-Saharan Africa | Central African Republic | 2009 | 2001-2006 | 88 | 24 | 7 | 20 | 15 | 0 | 22 | 0 | 0 | 0 | 0 | 0 |
| Maternal colonisation | Brochet(102) | Sub-Saharan Africa | Senegal | 2009 | 2001-2006 | 75 | 13 | 4 | 9 | 16 | 0 | 33 | 0 | 0 | 0 | 0 | 0 |
| Maternal colonisation | Moyo(103) | Sub-Saharan Africa | Zimbabwe | 2002 |  | 117 | 17 | 6 | 5 | 53 | 6 | 28 | 0 | 0 | 0 |  | 2 |
| Maternal colonisation | Moyo(104) | Sub-Saharan Africa | Zimbabwe | 2000 |  | 91 | 10 | 3 | 1 | 38 | 3 | 34 |  |  |  |  | 2 |
| Maternal colonisation | Suara(105) | Sub-Saharan Africa | Gambia | 1994 | pre 2000-2000 | 32 |  |  | 9 | 2 | 1 | 12 | 0 |  |  |  | 2 |
| Maternal colonisation | Cutland(106) | Sub-Saharan Africa | South Africa | unpublished | 2013-2018 | 811 | 305 | 60 | 77 | 210 | 20 | 113 | 0 | 0 | 0 | 0 | 26 |
| Maternal colonisation | Khodaei(107) | Western Asia | Iran | 2018 | 2013-2018 | 26 | 0 | 0 | 3 | 20 | 0 | 3 | 0 | 0 | 0 |  | 0 |
| Maternal colonisation | Bornasi(108) | Western Asia | Iran | 2016 |  | 60 | 11 | 4 | 10 | 27 | 0 | 8 | 0 | 0 | 0 | 0 | 0 |
| Maternal colonisation | Sadeh(109) | Western Asia | Iran | 2016 | 2013-2018 | 30 | 5 | 2 | 6 | 15 | 0 | 2 | 0 | 0 | 0 |  | 0 |
| Maternal colonisation | Beigverdi(110) | Western Asia | Iran | 2014 | 2013-2018 | 41 | 0 | 3 | 6 | 27 | 0 | 2 | 0 | 0 | 0 |  | 3 |
| Maternal colonisation | Udo(111) | Western Asia | Kuwait | 2013 | 2007-2012 | 154 | 16 | 5 | 16 | 30 | 5 | 59 | 4 | 1 | 1 |  | 17 |
| Maternal colonisation | Jannati(112) | Western Asia | Iran | 2011 | 2007-2012 | 56 | 4 | 5 | 7 | 6 | 7 | 11 | 6 | 3 | 3 |  | 4 |
| Maternal colonisation | Seoud(113) | Western Asia | Lebanon | 2010 | 2001-2006 | 137 | 20 | 9 | 15 | 22 | 1 | 31 | 0 | 0 | 0 |  | 39 |
| Maternal colonisation | Yenisehirli(114) | Western Asia | Turkey | 2006 | 2001-2006 | 98 | 7 | 24 | 2 | 33 | 3 | 18 |  |  |  |  | 11 |
| Maternal colonisation | Al-Sweih(115) | Western Asia | Kuwait | 2005 |  | 124 | 11 | 3 | 10 | 33 | 1 | 27 | 11 | 6 | 0 |  | 22 |
| Maternal colonisation | Eren(116) | Western Asia | Turkey | 2005 | 2001-2006 | 54 | 14 | 2 | 16 | 10 | 1 | 0 |  |  |  |  | 11 |
| Maternal colonisation | Amin(117) | Western Asia | United Arab Emirates | 2002 | pre 2000-2000 | 57 | 12 | 2 | 2 | 10 | 15 | 7 |  |  |  |  | 9 |
| Maternal GBS disease | Deutscher(118) | Developed | United States of America | 2011 | 2007-2012 | 53 | 11 | 5 | 12 | 9 | 5 | 11 | 0 | 0 | 0 |  | 0 |
| Maternal GBS disease | Phares(119) | Developed | United States of America | 2008 | 2001-2006 | 172 | 52 | 0 | 22 | 48 | 0 | 31 | 0 | 0 | 0 |  | 19 |
| Maternal GBS disease | Zaleznik(120) | Developed | United States of America | 2000 | pre 2000-2000 | 53 | 18 | 1 | 7 | 13 | 0 | 12 | 0 | 0 | 0 |  | 2 |
| Maternal GBS disease | Tyrrell(121) | Developed | Canada | 2000 | pre 2000-2000 | 11 | 3 | 1 | 0 | 2 | 0 | 4 | 0 | 1 | 0 |  | 0 |
| Maternal GBS disease | Pass(122) | Developed | United States of America | 1982 | pre 2000-2000 | 21 | 5 | 3 | 3 | 8 | 0 | 0 | 0 | 0 | 0 |  | 2 |
| Maternal GBS disease | Lu(123) | Eastern Asia | China | 2018 | 2013-2018 | 11 | 1 | 1 | 0 | 5 | 0 | 3 | 0 | 0 | 0 | 0 | 1 |
| Stillbirth GBS-associated | Davies(124) | Developed | Canada | 2001 | pre 2000-2000 | 13 | 5 | 2 | 1 | 3 | 0 | 2 | 0 | 0 | 0 |  | 0 |
| Stillbirth GBS-associated | Bassat(125) | Sub-Saharan Africa | Mozambique | unpublished | 2013-2018 | 4 | 0 | 0 | 0 | 2 | 0 | 1 | 1 | 0 | 0 | 0 | 0 |
| Stillbirth GBS-associated | Seale(93) | Sub-Saharan Africa | Kenya | 2016 | 2013-2018 | 3 | 0 | 0 | 0 | 1 | 0 | 2 | 0 | 0 | 0 | 0 | 0 |
| Stillbirth GBS-associated | Kwatra | Sub-Saharan Africa | South Africa | unpublished | 2014-2015 | 14 | 3 | 1 | 0 | 6 | 0 | 4 | 0 | 0 | 0 | 0 | 0 |
| Infant GBS disease | O´Sullivan(126) | Developed | United Kingdom & Ireland | unpublished | 2013-2018 | 402 | 69 | 23 | 18 | 241 | 9 | 26 | 3 | 1 | 1 | 0 | 11 |
| Infant GBS disease | Nanduri(127) | Developed | United States of America | 2019 | 2007-2012 | 1734 | 413 | 130 | 163 | 723 | 108 | 197 |  |  |  |  |  |
| Infant GBS disease | Romain(128) | Developed | France | 2018 | 2007-2012 | 339 | 31 | 10 | 4 | 286 | 2 | 6 | 0 | 0 | 0 | 0 | 0 |
| Infant GBS disease | Creti(129) | Developed | Italy | 2017 | 2007-2012 | 91 | 8 | 2 | 4 | 63 | 1 | 9 | 0 | 0 | 0 | 3 | 1 |
| Infant GBS disease | Hayes(130) | Developed | Ireland | 2017 | 2013-2018 | 7 | 1 | 0 | 1 | 3 | 0 | 1 | 1 | 0 | 0 | 0 | 0 |
| Infant GBS disease | Martins(131) | Developed | Portugal | 2017 | 2007-2012 | 214 | 48 | 11 | 9 | 126 | 3 | 9 | 1 | 0 | 1 | 1 | 5 |
| Infant GBS disease | Six(132) | Developed | France | 2016 | 2007-2012 | 16 | 1 | 1 | 0 | 12 | 1 | 0 | 1 | 0 | 0 |  | 0 |
| Infant GBS disease | Alhhazmi(133) | Developed | Canada | 2016 | 2007-2012 | 264 | 49 | 24 | 22 | 123 | 11 | 26 | 2 | 0 | 0 | 0 | 7 |
| Infant GBS disease | Ko Danny(8) | Developed | Australia | 2015 | 2007-2012 | 108 | 26 | 6 | 5 | 46 | 2 | 18 | 4 | 1 | 0 | 0 | 0 |
| Infant GBS disease | Almeida(134) | Developed | France | 2015 | 2007-2012 | 18 | 5 | 0 | 0 | 12 | 0 | 1 | 0 | 0 | 0 | 0 | 0 |
| Infant GBS disease | Joubrel(135) | Developed | France | 2015 | 2007-2012 | 430 | 62 | 18 | 12 | 308 | 4 | 26 | 0 | 0 | 0 |  | 0 |
| Infant GBS disease | Chang(136) | Developed | Japan | 2014 | 2007-2012 | 58 | 18 | 5 | 2 | 28 | 2 | 1 | 1 | 0 | 1 | 0 | 0 |
| Infant GBS disease | Brzychczy-Wloch(137) | Developed | Poland | 2014 | 2007-2012 | 22 | 2 | 0 | 6 | 11 | 0 | 3 | 0 | 0 | 0 |  | 0 |
| Infant GBS disease | Teatero(138) | Developed | Canada | 2014 | 2007-2012 | 93 | 13 | 6 | 4 | 59 | 1 | 8 | 2 | 0 | 0 | 0 | 0 |
| Infant GBS disease | Bekker(139) | Developed | Netherlands | 2014 | pre 2000-2000 | 816 | 171 | 0 | 0 | 645 | 0 | 0 | 0 | 0 | 0 |  | 0 |
| Infant GBS disease | Morozumi(140) | Developed | Japan | 2014 | 2007-2012 | 138 | 30 | 16 | 5 | 81 | 1 | 0 | 4 | 0 | 1 |  | 0 |
| Infant GBS disease | Matsubara(141) | Developed | Japan | 2013 | 2007-2012 | 64 | 20 | 7 | 0 | 32 | 1 | 2 | 1 | 0 | 1 |  | 0 |
| Infant GBS disease | Oladottir(142) | Developed | Iceland | 2011 | pre 2000-2000 | 70 | 15 | 9 | 5 | 31 | 2 | 8 | 0 | 0 | 0 |  | 0 |
| Infant GBS disease | Imperi(143) | Developed | Italy | 2011 | 2007-2012 | 75 | 7 | 4 | 5 | 54 | 1 | 4 | 0 | 0 | 0 | 0 | 0 |
| Infant GBS disease | Martins(144) | Developed | Spain | 2011 | 2001-2006 | 212 | 47 | 13 | 11 | 118 | 3 | 14 |  |  |  |  | 6 |
| Infant GBS disease | Matsubara(145) | Developed | Japan | 2009 | 2001-2006 | 6 | 2 | 1 | 0 | 1 | 0 | 0 | 0 | 0 | 1 |  | 1 |
| Infant GBS disease | Zhao(146) | Developed | Australia & New Zealand | 2008 | pre 2000-2000 | 257 | 71 | 33 | 17 | 104 | 3 | 26 | 2 | 0 | 1 |  | 0 |
| Infant GBS disease | Martins(33) | Developed | Portugal | 2007 | 2001-2006 | 58 | 18 | 2 | 0 | 26 | 2 | 7 | 0 | 0 | 2 |  | 1 |
| Infant GBS disease | Trijbels-Smeulders(147) | Developed | Netherlands | 2006 | pre 2000-2000 | 198 | 37 | 6 | 16 | 109 | 1 | 14 | 1 | 0 | 0 |  | 14 |
| Infant GBS disease | Fluegge(148) | Developed | Germany | 2005 | 2001-2006 | 291 | 43 | 13 | 15 | 193 | 2 | 22 |  |  |  |  | 3 |
| Infant GBS disease | Davies(149) | Developed | Canada | 2004 | pre 2000-2000 | 28 | 0 | 0 | 0 | 28 | 0 | 0 | 0 | 0 | 0 |  | 0 |
| Infant GBS disease | Strakova(150) | Developed | Czech Republic | 2004 | 2001-2006 | 170 | 35 | 8 | 31 | 62 | 3 | 25 | 4 | 2 | 0 |  | 0 |
| Infant GBS disease | Figueira-Coelho(151) | Developed | Portugal | 2004 | pre 2000-2000 | 17 | 5 | 0 | 1 | 6 | 2 | 3 | 0 | 0 | 0 |  | 0 |
| Infant GBS disease | Persson(152) | Developed | Sweden | 2004 | pre 2000-2000 | 46 | 5 | 2 | 1 | 30 | 0 | 7 | 1 | 0 | 0 |  | 0 |
| Infant GBS disease | Ekelund(153) | Developed | Denmark | 2004 | pre 2000-2000 | 512 | 95 | 37 | 29 | 294 | 5 | 21 | 0 | 0 | 0 |  | 31 |
| Infant GBS disease | Bidet(154) | Developed | France | 2003 | pre 2000-2000 | 104 | 0 | 0 | 0 | 104 | 0 | 0 | 0 | 0 | 0 |  | 0 |
| Infant GBS disease | Hoshina(155) | Developed | Japan | 2002 | pre 2000-2000 | 282 | 42 | 32 | 15 | 131 | 9 | 7 | 19 | 1 | 12 |  | 14 |
| Infant GBS disease | Davies(124) | Developed | Canada | 2001 | pre 2000-2000 | 118 | 21 | 6 | 6 | 63 | 0 | 17 | 0 | 0 | 0 |  | 5 |
| Infant GBS disease | Guan(156) | Eastern Asia | China | 2018 | 2013-2018 | 68 | 2 | 10 |  | 53 |  | 3 |  |  |  |  |  |
| Infant GBS disease | Liu(157) | Eastern Asia | China | 2018 | 2013-2018 | 86 | 4 | 15 | 0 | 60 | 0 | 7 | 0 | 0 | 0 | 0 | 0 |
| Infant GBS disease | Lu(123) | Eastern Asia | China | 2018 | 2013-2018 | 44 | 3 | 6 | 1 | 26 | 0 | 6 | 0 | 0 | 0 | 0 | 2 |
| Infant GBS disease | Cho(158) | Eastern Asia | South Korea | 2017 | 2007-2012 | 21 | 3 | 3 | 3 | 7 | 0 | 4 |  |  |  |  | 1 |
| Infant GBS disease | Lo(159) | Eastern Asia | Taiwan | 2017 | 2001-2006 | 158 | 28 | 16 | 6 | 86 | 0 | 6 | 6 | 1 | 0 | 0 | 9 |
| Infant GBS disease | Ip(160) | Eastern Asia | Hong Kong | 2016 | 2001-2006 | 113 | 19 | 14 | 4 | 72 | 0 | 3 | 1 | 0 | 0 | 0 | 0 |
| Infant GBS disease | Wang(161) | Eastern Asia | China | 2015 | 2007-2012 | 40 | 3 | 2 | 0 | 34 | 0 | 1 | 0 | 0 | 0 |  | 0 |
| Infant GBS disease | Yoon(162) | Eastern Asia | South Korea | 2015 | pre 2000-2000 | 56 | 8 | 6 | 0 | 25 | 0 | 16 | 1 | 0 | 0 |  | 0 |
| Infant GBS disease | Rivera(163) | Eastern Asia | Hong Kong | 2015 |  | 15 | 1 | 6 | 0 | 7 | 0 | 1 | 0 | 0 | 0 |  | 0 |
| Infant GBS disease | Rivera(163) | Latin America and the Caribbean | Panama | 2015 |  | 21 | 5 | 0 | 1 | 15 | 0 | 0 | 0 | 0 | 0 |  | 0 |
| Infant GBS disease | Rivera(163) | Latin America and the Caribbean | Dominican Republic | 2015 |  | 57 | 13 | 10 | 5 | 27 | 0 | 2 | 0 | 0 | 0 |  | 0 |
| Infant GBS disease | Souza(164) | Latin America and the Caribbean | Brazil | 2013 | 2007-2012 | 17 | 10 | 1 | 1 | 2 | 0 | 3 | 0 | 0 | 0 |  | 0 |
| Infant GBS disease | Fiolo(165) | Latin America and the Caribbean | Brazil | 2012 | 2007-2012 | 6 | 2 | 0 | 0 | 2 | 0 | 2 | 0 | 0 | 0 | 0 | 0 |
| Infant GBS disease | Martinez(166) | Latin America and the Caribbean | Chile | 2004 | pre 2000-2000 | 10 | 3 | 0 | 0 | 7 |  |  |  |  |  |  | 0 |
| Infant GBS disease | Lopardo(167) | Latin America and the Caribbean | Argentina | 2003 | pre 2000-2000 | 12 | 4 | 0 | 1 | 7 | 0 | 0 | 0 | 0 | 0 |  | 0 |
| Infant GBS disease | Moraleda(77) | Northern Africa | Morocco | 2018 | 2013-2018 | 5 | 0 | 0 | 2 | 1 | 1 | 1 | 0 | 0 | 0 | 0 | 0 |
| Infant GBS disease | Sigauque(168) | Sub-Saharan Africa | Mozambique | 2018 | 2013-2018 | 35 | 1 | 0 | 0 | 33 | 0 | 1 | 0 | 0 | 0 | 0 | 0 |
| Infant GBS disease | Seale(93) | Sub-Saharan Africa | Kenya | 2016 | 2001-2006 | 70 | 10 | 4 | 1 | 54 | 1 | 0 | 0 | 0 | 0 | 0 | 0 |
| Infant GBS disease | Dangor(169) | Sub-Saharan Africa | South Africa | 2016 | 2007-2012 | 635 | 179 | 22 | 23 | 353 | 7 | 50 | 1 | 0 | 0 | 0 | 0 |
| Infant GBS disease | Cutland(170) | Sub-Saharan Africa | South Africa | 2015 | 2001-2006 | 213 | 41 | 8 | 8 | 138 | 5 | 13 | 0 | 0 | 0 |  | 0 |
| GBS disease in elderly | Lopes(171) | Developed | Portugal | 2017 | 2007-2012 | 360 | 113 | 84 | 22 | 39 | 6 | 70 | 1 | 0 | 1 | 6 | 18 |
| GBS disease in elderly | Bjornsdottir(172) | Developed | Iceland | 2016 | pre 2000-2000 | 76 | 15 | 13 | 9 | 9 | 7 | 17 | 1 | 0 | 0 | 0 | 5 |
| GBS disease in elderly | Teatero(138) | Developed | Canada | 2014 | 2007-2012 | 308 | 53 | 42 | 34 | 55 | 21 | 90 | 4 | 1 | 0 | 2 | 6 |
| GBS disease in elderly | Martins(173) | Developed | Portugal | 2012 | 2001-2006 | 130 | 36 | 12 | 15 | 20 | 1 | 30 | 1 | 1 | 0 | 0 | 14 |
| GBS disease in elderly | Kothari(174) | Developed | United States of America | 2009 | 2001-2006 | 654 | 138 | 72 | 77 | 74 |  | 229 |  |  |  |  |  |
| GBS disease in elderly | Skoff(175) | Developed | United States of America | 2009 | 2001-2006 | 956 | 226 | 84 | 134 | 112 | 37 | 301 | 0 | 1 | 0 |  | 61 |
| GBS disease in elderly | Wessels(176) | Developed | United States of America | 1998 | pre 2000-2000 | 4 | 0 | 0 | 0 | 3 | 0 | 1 | 0 | 0 | 0 |  | 0 |
| GBS disease in elderly | Harrison(177) | Developed | United States of America | 1995 | pre 2000-2000 | 12 | 3 | 2 | 1 | 1 |  | 5 |  |  |  |  | 0 |
| GBS disease in elderly | Lopardo(167) | Latin America and the Caribbean | Argentina | 2003 | pre 2000-2000 | 9 | 5 | 0 | 1 | 2 | 1 | 0 | 0 | 0 | 0 |  | 0 |
| GBS disease in elderly | Suhaimi(80) | South-eastern Asia | Malaysia | 2017 | 2013-2018 | 8 | 2 | 0 | 0 | 2 | 0 | 2 | 1 | 1 | 0 | 0 | 0 |
| GBS disease in elderly | Eskandarian(178) | South-eastern Asia | Malaysia | 2013 | 2007-2012 | 8 | 3 | 0 | 0 | 0 | 0 | 2 | 3 | 0 | 0 | 0 | 0 |

## Supplementary Table S5: Study characteristics and data abstracted for MLST and virulence proteins papers

| **Outcome** | **Author** | **Region** | **Country** | **Total isolates sequenced/PCR** | **ST17 isolates** | **CC1** | **CC 23** | **CC19** | **CC10** | **CC17** | **CC12** | **alp1/epsilon** | **alp2** | **alp3** | **rib** | | **bca/ alpha C** | | **none** | **PI-2a** | **PI-2b** | **PI-1+PI-2a** | **PI-1+PI-2b** | |
| --- | --- | --- | --- | --- | --- | --- | --- | --- | --- | --- | --- | --- | --- | --- | --- | --- | --- | --- | --- | --- | --- | --- | --- | --- |
| maternal colonisation | Beauruelle(179) | Developed region | France | 18 | 0 |  |  |  |  |  |  |  |  |  |  | |  | |  |  |  |  |  | |
| maternal colonisation | Brzychczy-Włoch(18) | Developed region | Poland | 169 |  |  |  |  |  |  |  | 44 | 36 | 23 | 37 | | 29 | | 0 |  |  |  |  | |
| maternal colonisation | Dmitriev(180) | Eastern Asia | China and Russia | 45 |  |  |  |  |  |  |  |  |  |  |  | | 27 | |  |  |  |  |  | |
| maternal colonisation | Elikwu(181) | Sub-Saharan Africa | Nigeria | 46 | 12 |  |  |  |  |  |  |  |  |  |  | |  | |  |  |  |  |  | |
| maternal colonisation | Lee(52) | Eastern Asia | South Korea | 19 | 0 | 5 |  | 5 | 4 |  |  |  |  |  |  | |  | |  |  |  |  |  | |
| maternal colonisation | Li(182) | Eastern Asia | China | 92 | 2 |  | 1 | 1 | 2 | 2 | 2 |  |  |  |  | |  | |  |  |  |  |  | |
| maternal colonisation | Liakupolos(183) | Developed region | Greece | 171 | 7 | 47 | 41 | 36 | 35 | 7 | 0 |  |  |  |  | |  | |  |  |  |  |  | |
| maternal colonisation | Medugu(90) | Sub-Saharn Africa | Nigeria | 35 | 3 | 6 | 5 | 19 |  | 3 | 1 |  |  |  |  | |  | |  | 5 | 3 | 27 |  | |
| maternal colonisation | Oviedo(68) | Latin America and the Caribbean | Argentina | 88 |  |  |  |  |  |  |  |  |  |  | 67 | | 78 | |  |  |  |  |  | |
| maternal colonisation | Sadaka(184) | Northern Africa | Egypt | 53 |  |  |  |  |  |  |  |  |  |  | 42 | | 0 | |  |  |  |  |  | |
| maternal colonisation | Springman(185) | Developed region | Canada | 99 |  |  |  |  |  |  |  |  |  |  |  | |  | |  | 13 | 0 | 66 | 20 | |
| maternal colonisation | Wang(53) | Eastern Asia | China | 104 | 15 | 3 | 3 | 26 | 12 | 15 | 15 | 28 | 3 | | | 40 | | 28 | 5 |  |  |  |  |  |
| maternal colonisation | Creti(129) | Developed region | Italy | 126 | 24 |  |  |  |  |  |  |  |  |  |  | |  | |  |  |  |  |  | |
| maternal colonisation | Ko(8) | Australia and New Zealand | Australia | 282 |  | 50 | 56 | 83 |  | 20 |  | 105 |  | 68 | 154 | |  | |  |  |  |  |  | |
| maternal colonisation | Madzivhandila(186) | Sub-Saharn Africa | South Africa | 541 |  |  |  |  |  |  |  |  |  |  |  | |  | |  | 183 | 2 | 155 | 201 | |
| maternal colonisation | Manning(187) | Developed region | Canada | 232 | 14 | 65 | 57 | 39 |  | 14 | 42 |  |  |  |  | |  | |  |  |  |  |  | |
| maternal colonisation | Seale(93) | Sub-Saharn Africa | Kenya | 934 | 183 | 114 | 208 | 173 | 148 | 267 |  |  |  |  |  | |  | |  |  |  |  |  | |
| maternal colonisation | Tsai(188) | Eastern Asia | Taiwan | 100 | 14 | 57 | 6 | 8 |  | 14 | 9 |  |  |  |  | |  | |  |  |  |  |  | |
| maternal colonisation | Bergal(78) | Northern Africa | Algeria and France | 93 | 3 |  |  |  |  |  |  |  |  |  |  | |  | |  |  |  |  |  | |
| maternal colonisation | Brochet(102) | Sub-Saharn Africa | Senegal and Central African Republic | 163 | 14 | 32 | 37 | 47 |  | 19 |  |  |  |  |  | |  | |  |  |  |  |  | |
| maternal colonisation | Lu(57) | Eastern Asia | China | 160 |  | 24 | 27 | 56 |  | 14 | 28 | 40 | 35 | | | 60 | | 13 | 4 | 70 | 21 | 67 | 2 |  |
| maternal colonisation | Rojo-Bezares(7) | Developed region | Spain | 65 | 4 | 14 | 7 | 25 |  | 4 |  | 15 | 10 | | | 24 | | 5 |  |  |  |  |  |  |
| maternal colonisation | Wang(56) | Eastern Asia | China | 56 | 4 |  | 8 | 20 |  |  | 14 |  |  |  |  | |  | |  |  |  |  |  | |
| maternal colonisation | Bisharat(38) | Western Asia | Israel | 104 | 7 |  |  |  |  |  |  |  |  |  |  | |  | |  |  |  |  |  | |
| maternal colonisation | Gajic(189) | Developed region | Serbia | 34 | 7 |  |  |  |  |  |  |  |  |  |  | |  | |  |  |  |  |  | |
| maternal colonisation | Jones(190) | Developed region | United Kingdom | 190 | 19 | 38 | 44 | 38 | 33 | 23 |  |  |  |  |  | |  | |  |  |  |  |  | |
| infant colonisation | Emaneini(191) | Western Asia | Iran | 19 | 0 | 1 | 0 | 13 | 4 | 0 | 0 |  |  |  | 14 | | 19 | |  |  |  |  |  | |
| infant colonisation | Gajic(189) | Developed region | Serbia | 5 | 1 |  |  |  |  |  |  |  |  |  |  | |  | |  |  |  |  |  | |
| infant colonisation | Guo(192) | Eastern Asia | China | 24 | 2 | 1 | 2 | 6 | 3 | 2 | 2 | 7 | 1 | | | 8 | | 6 | 0 |  |  |  |  |  |
| infant colonisation | Hsu(193) | Eastern Asia | Taiwan | 12 | 0 | 7 | 2 | 2 | 0 | 0 | 0 |  |  |  |  | |  | |  |  |  |  |  | |
| infant colonisation | Toyofuku(194) | Eastern Asia | Japan | 45 | 1 |  |  |  |  |  |  |  |  |  |  | |  | |  |  |  |  |  | |
| infant colonisation | Brzychczy-Wloch(137) | Developed region | Poland | 19 | 0 |  | 14 | 2 | 1 | 0 | 0 | 5 | 10 | 1 | 2 | | 2 | | 0 |  |  |  |  | |
| infant colonisation | Elikwu(181) | Sub-Saharn Africa | Nigeria |  | 4 |  |  |  |  |  |  |  |  |  |  | |  | |  |  |  |  |  | |
| infant colonisation | Medugu(90) | Sub-Saharn Africa | Nigeria | 35 | 3 | 6 | 5 | 19 |  | 3 | 1 |  |  |  |  | |  | |  | 5 | 3 | 27 |  | |
| maternal invasive | Meehan(13) | Developed region | Ireland | 29 |  | 3 | 12 | 4 | 0 | 7 | 3 |  |  |  |  | |  | |  |  |  |  |  | |
| infant invasive | Al Safadi(195) | Developed region | France | 50 |  |  | 5 | 16 | 1 | 28 |  |  |  |  |  | |  | |  |  |  |  |  | |
| infant invasive | Almeida(134) | Developed region | France | 21 | 13 |  |  |  |  |  |  |  |  |  |  | |  | |  |  |  |  |  | |
| infant invasive | Bekker(139) | Developed region | Netherlands | 167 | 44 | 9 | 35 | 51 |  | 49 | 20 |  |  |  |  | |  | |  |  |  |  |  | |
| infant invasive | Bellais(196) | Developed region | France | 5 | 4 |  |  |  |  | 5 |  |  |  |  |  | |  | |  |  |  |  |  | |
| infant invasive | Bergseng(197) | Developed region | Norway | 96 | 23 | 26 | 8 | 17 | 0 | 30 | 0 | 16 | 18 | | | 48 | | 14 |  |  |  |  |  |  |
| children invasive | Bergseng(198) | Developed region | Norway | 55 |  |  |  |  |  |  |  | 6 | 13 | | | 29 | | 3 | 2 |  |  |  |  |  |
| infant invasive | Bisharat(38) | Western Asia | Israel | 50 | 11 |  |  |  |  |  |  |  |  |  |  | |  | |  |  |  |  |  | |
| infant invasive | Bjornsdottir(199) | Developed region | Iceland | 98 | 28 | 11 | 17 | 19 | 13 | 31 | 0 | 19 |  | 7 | 50 | | 22 | |  | 18 | 3 | 44 | 33 | |
| infant invasive | Brzychczy-Wloch(137) | Developed region | Poland | 22 | 4 | 1 | 6 | 5 | 3 | 4 | 0 | 1 | 2 | 1 | 13 | | 5 | |  |  |  |  |  | |
| infant invasive | Campisi(200) | Eastern Asia | China | 26 | 14 |  |  |  |  |  |  |  |  |  |  | |  | |  | 4 | 13 | 8 | 1 | |
| infant invasive | Creti(129) | Developed region | Italy | 48 | 26 |  |  |  |  |  |  |  |  |  |  | |  | |  |  |  |  |  | |
| infant invasive | Fluegge(201) | Developed region | Germany | 188 | 128 |  | 8 | 34 |  | 135 |  | 17 | 22 | | | 87 | | 20 | 25 |  |  |  |  |  |
| infant invasive | Gherardi(202) | Developed region | Italy | 11 | 1 | 1 | 2 | 1 |  | 3 |  | 3 | 0 | 2 | 4 | | 2 | |  |  |  |  |  | |
| infant invasive | Guan(156) | Eastern Asia | China | 68 | 43 |  |  |  |  |  |  |  |  |  |  | |  | |  |  |  |  |  | |
| infant invasive | Hayes(130) | Developed region | Ireland | 7 | 3 |  |  |  |  |  |  |  |  |  |  | |  | |  |  |  |  |  | |
| infant invasive | Imperi(143) | Developed region | Italy | 75 | 10 of 32 | 5 | 6 | 6 | 0 | 49 | 5 | 8 | 1 | 3 | 55 | | 8 | |  |  |  |  |  | |
| infant invasive | Jauneikaite(203) | Developed region | United Kingdom | 12 | 3 |  |  |  |  |  |  |  |  |  |  | |  | |  |  |  |  |  | |
| infant invasive | Jones(190) | Developed region | United Kingdom | 109 | 33 |  |  |  |  |  |  |  |  |  |  | |  | |  |  |  |  |  | |
| infant invasive | Kang(204) | Eastern Asia | South Korea | 98 | 19 |  |  | 29 | 13 |  |  |  |  |  |  | |  | |  |  |  |  |  | |
| infant invasive | Ko(8) | Australia and New Zealand | Australia | 108 |  | 26 | 24 | 17 |  |  |  | 28 |  | 18 | 46 | |  | |  |  |  |  |  | |
| infant invasive | Lo(159) | Eastern Asia | Taiwan | 83 | 7 |  |  |  |  |  |  |  |  |  |  | |  | |  |  |  |  |  | |
| infant invasive | Lu(123) | Eastern Asia | China | 50 | 17 | 3 | 2 | 9 | 14 | 19 | 0 |  |  |  |  | |  | |  | 5 | 3 | 24 | 18 | |
| infant invasive | Luan(205) | Developed region | Sweden | 102 | 28 | 12 | 15 | 29 | 0 | 34 | 0 |  |  |  |  | |  | |  |  |  |  |  | |
| infant invasive | MacFarquhar(206) | Developed region | United States of America | 5 | 0 |  |  |  |  |  |  |  |  |  |  | |  | |  |  |  |  |  | |
| infant invasive | Madzivhandila(186) | Sub-Saharn Africa | South Africa | 284 |  |  |  |  |  |  |  |  |  |  |  | |  | |  | 63 | 0 | 50 | 171 | |
| infant invasive | Manning(187) | Developed region | Canada | 183 | 48 | 32 | 33 | 43 | 0 | 56 | 19 |  |  |  |  | |  | |  |  |  |  |  | |
| infant invasive | Manning(207) | Developed region | United States of America | 100 |  |  |  |  |  |  |  |  |  |  | 28 | | 29 | |  |  |  |  |  | |
| infant invasive | Margarit(208) | Developed region | United States of America and Italy | 204 |  |  |  |  |  |  |  |  |  |  |  | |  | |  | 54 | 1 | 81 | 68 | |
| infant invasive | Martins(33) | Developed region | Portugal | 33 | 5 |  |  |  |  |  |  |  |  |  |  | |  | |  |  |  |  |  | |
| infant invasive | Martins(144) | Developed region | Spain | 212 | 85 |  |  |  |  |  |  | 41 | 0 | 13 | 123 | | 34 | | 1 |  |  |  |  | |
| infant invasive | Martins(131) | Developed region | Portugal | 218 | 101 | 21 | 50 | 25 | 9 | 109 | 0 | 40 | 1 | 14 | 135 | | 28 | |  | 49 | 9 | 60 | 100 | |
| infant invasive | Meehan(13) | Developed region | Ireland | 72 |  | 11 | 9 | 5 | 0 | 33 | 6 |  |  |  |  | |  | |  |  |  |  |  | |
| infant invasive | Morozumi(140) | Eastern Asia | Japan | 150 | 44 | 14 | 30 | 45 | 17 | 44 | 0 |  |  |  |  | |  | |  |  |  |  |  | |
| infant invasive | Nanduri(127) | Developed region | United States of America | 173 | 58 |  |  |  |  |  |  |  |  |  |  | |  | |  |  |  |  |  | |
| infant invasive | Persson(209) | Developed region | Sweden | 123 |  |  |  |  |  |  |  | 22 | 1 | 19 | 74 | | 0 | | 1 |  |  |  |  | |
| infant invasive | Poyart(210) | Developed region | France | 109 | 75 |  |  |  |  |  |  |  |  |  |  | |  | |  |  |  |  |  | |
| infant invasive | Puopulo(211) | Developed region | United States of America | 21 |  |  |  |  |  |  |  | 8 |  | 5 | 6 | | 2 | |  |  |  |  |  | |
| infant invasive | Salloum(212) | Developed region | France | 67 | 44 | 3 | 9 | 4 | 0 | 45 | 0 |  |  |  |  | |  | |  |  |  |  |  | |
| infant invasive | Seale(93) | Sub-Saharn Africa | Kenya | 73 |  | 2 | 11 | 4 | 5 | 51 | 0 |  |  |  |  | |  | |  |  |  |  |  | |
| infant invasive | Sigauque(213) | Sub-Saharn Africa | Mozambique | 35 | 24 | 1 | 1 | 0 | 0 | 33 | 0 | 1 | 1 | | | 33 | | 0 | 0 | 1 |  | 1 | 33 |  |
| infant invasive | Six(132) | Developed region | France | 16 | 8 |  |  |  |  |  |  |  |  |  |  | |  | |  |  |  |  |  | |
| infant invasive | Tien(214) | Eastern Asia | Taiwan | 17 | 6 | 1 | 4 | 3 | 0 | 7 | 2 |  |  |  |  | |  | |  |  |  |  |  | |
| infant invasive | van der Mee-Marquet(215) | Developed region | France | 120 |  | 11 | 31 | 13 | 6 | 56 |  |  |  |  |  | |  | |  |  |  |  |  | |
| infant invasive | Veeraraghavan(216) | Southern Asia | India | 4 | 0 |  |  |  |  |  |  |  |  |  |  | |  | |  |  |  |  |  | |
| infant invasive | Wang(161) | Eastern Asia | China | 40 | 32 |  |  |  |  |  |  |  |  |  |  | |  | |  |  |  |  |  | |
| infant invasive | Wu(217) | Eastern Asia | China | 29 | 18 |  | 1 | 5 | 4 | 19 |  |  |  |  |  | |  | |  |  |  |  |  | |
| infant invasive | Gajic(189) | Developed region | Serbia | 24 | 15 |  |  |  |  |  |  |  |  |  |  | |  | |  |  |  |  |  | |
| infant invasive | Liakupolos(183) | Developed region | Greece | 46 | 31 | 0 | 3 | 6 | 3 | 34 | 0 |  |  |  |  | |  | |  |  |  |  |  | |
| infant invasive | Tsai(188) | Eastern Asia | Taiwan | 127 | 62 | 12 | 13 | 14 |  | 63 | 15 |  |  |  |  | |  | |  |  |  |  |  | |
| infantinvasive | Sadowy(218) | Developed region | Poland | 23 |  | 7 | 8 | 1 |  | 7 |  |  |  |  |  | |  | |  |  |  |  |  | |
| infant invasive | Springman(185) | Developed region | Canada | 120 |  |  |  |  |  |  |  |  |  |  |  | |  | |  | 7 | 0 | 59 | 54 | |
| children invasive | Florindo(219) | Sub-Saharn Africa | Angola | 21 | 4 |  |  |  |  |  |  |  |  |  |  | |  | |  |  |  |  |  | |
| adult invasive | Björnsdottir(172) | Developed region | Iceland | 145 | 7 | 44 | 27 | 20 | 34 | 7 | 0 | 32 | 3 | 28 | 29 | | 52 | | 1 |  |  |  |  | |
| adult invasive | Gabrielsen(220) | Developed region | Norway | 10 |  | 1 |  | 1 |  |  |  |  |  |  |  | |  | |  |  |  |  |  | |
| adult invasive | Lopes(171) | Developed region | Portugal | 555 | 22 | 224 | 157 | 79 | 37 | 32 | 0 | 113 | 7 | 203 | 117 | | 115 | | 7 |  |  |  |  | |
| adult invasive | Lu(221) | Eastern Asia | China | 51 | 8 |  |  |  |  |  |  |  |  |  |  | |  | |  |  |  |  |  | |
| adult invasive | Martins(173) | Developed region | Portugal | 225 | 5 |  |  |  |  |  |  | 70 | 7 | 25 | 55 | | 66 | | 2 |  |  |  |  | |
| adult invasive | Morozumi(222) | Eastern Asia | Japan | 443 | 7 | 157 | 41 | 48 | 175 | 7 |  |  |  |  |  | |  | |  |  |  |  |  | |
| adult invasive | Ryu(223) | Eastern Asia | South Korea | 41 |  | 18 | 4 | 6 | 13 |  |  |  |  |  |  | |  | |  |  |  |  |  | |
| adult invasive | Tsai(188) | Eastern Asia | Taiwan | 98 | 2 | 54 | 4 | 7 | 0 | 2 | 20 |  |  |  |  | |  | |  |  |  |  |  | |
| adult invasive | Jones(190) | Developed region | United Kingdom | 70 | 4 | 17 | 11 | 14 | 15 | 4 |  |  |  |  |  | |  | |  |  |  |  |  | |
| adult invasive | Luan(224) | Developed region | Sweden | 53 | 4 | 12 | 6 | 16 | 0 | 4 | 0 |  |  |  |  | |  | |  |  |  |  |  | |
| adult invasive | Margarit(208) | Developed region | United States of America and Italy | 76 |  |  |  |  |  |  |  |  |  |  |  | |  | |  |  |  |  |  | |
| adult invasive | Meehan(13) | Developed region | Ireland | 31 |  | 12 | 5 | 3 | 0 | 1 | 8 |  |  |  |  | |  | |  |  |  |  |  | |
| adult invasive | Persson(209) | Developed region | Sweden | 174 |  |  |  |  |  |  |  | 20 | 2 | 58 | 52 | | 10 | | 2 |  |  |  |  | |
| adult invasive | Sadowy(218) | Developed region | Poland | 13 |  | 3 | 8 | 0 |  | 1 |  |  |  |  |  | |  | |  |  |  |  |  | |
| adult invasive | Salloum(212) | Developed region | France | 75 | 5 | 25 | 15 | 7 | 0 | 6 | 0 |  |  |  |  | |  | |  |  |  |  |  | |
| adult invasive | Six(132) | Developed region | France | 31 | 2 |  |  |  |  |  |  |  |  |  |  | |  | |  |  |  |  |  | |
| adult invasive | Tien(225) | Eastern Asia | Taiwan | 17 | 0 | 10 | 1 | 1 | 0 | 0 | 5 |  |  |  |  | |  | |  |  |  |  |  | |

## Supplementary Table S6: Studies with number of strains ST17 differentiated by EOGBS and LOGBS.

| **Author** | **Country** | **Total isolates sequenced/PCR** | | | **Number isolates ST17** | | **ST17 from EOGBS** | | **ST17 from LOGBS** | |
| --- | --- | --- | --- | --- | --- | --- | --- | --- | --- | --- |
| Poyart(210) | France | 109 | | | 75 | | 22 | | 53 | |
| Bjornsdottir(199) | Iceland | 98 | | | 28 | | 9 | | 19 | |
| Lu(123) | China | 50 | | | 17 | | 9 | | 8 | |
| Almeida(134) | France | 21 | | | 13 | | 8 | | 5 | |
| Sigauque(168) | Mozambique | 35 | | | 24* | | 8 | | 14 | |
| Kang(204) | South Korea | 98 | | | 19 | | 1 | | 18 | |
| Bergseng(197) | Norway | 96 | | | 23 | | 10 | | 13 | |
| Nanduri(127) | United States of America | 173 | | | 58 | | 9 | | 49 | |
| Bellais(196) | France | 5 | | | 4 | | 0 | | 4 | |
| Jones(190) | United Kingdom | 109 | | | 33 | | 15 | | 18 | |
| Wu(217) | China | 29 | | | 18 | | 6 | | 12 | |
| Gajic(189) | Serbia | 24 | | | 15 | | 1 | | 14 | |
| Imperi | Italy | 32 | | | 10 | | 3 | | 7 | |
| **Total** | | | 879 | 337 | | 101 | | 234 | |  |

*2 isolates with unknown date of onset

## Supplementary Table S7: number of non-typeable samples (NT) and proportion meta-analysis for each serotyping method

| **Serotyping method** | **NT samples** | **Total samples** | **Meta-analysis** | **95% CI** | **n studies** |
| --- | --- | --- | --- | --- | --- |
| Sequencing | 0 | 1023 | 0.0 | 0.0-0.0 | 4 |
| Latex agglutination and PCR | 47 | 4675 | 0.1 | 0.0-0.5 | 22 |
| Not specified | 46 | 1660 | 0.3 | 0.0-2.8 | 9 |
| PCR or multiplex PCR | 113 | 5496 | 0.5 | 0.0-1.5 | 46 |
| Serological methods | 735 | 16247 | 3.6 | 2.6-4.7 | 45 |
| Other methods | 21 | 146 | 5.0 | 1.5-10.0 | 6 |
| **Total** | 962 | 29247 |  |  | 192 |
|  |  |  |  |  |  |

## Supplementary Figure S1: Geographic distribution of available data for group B *Streptococcus* (GBS) serotypes for maternal colonisation.

##

Borders of countries/territories in map do not imply any political statement.

## Supplementary Figure S2: Geographic distribution of available data for group B *Streptococcus* (GBS) serotypes for infant GBS disease.

Borders of countries/territories in map do not imply any political statement.

## Supplementary Figure S3: Geographic distribution of available data for group B *Streptococcus* (GBS) serotypes for maternal invasive GBS disease.

Borders of countries/territories in map do not imply any political statement.

## Supplementary Figure S4: Geographic distribution of available data for group B *Streptococcus* (GBS) serotypes for GBS-associated stillbirths.

Borders of countries/territories in map do not imply any political statement.

## Supplementary Figure S5: Geographic distribution of available data for group B *Streptococcus* (GBS) serotypes for GBS invasive disease in elderly population

Borders of countries/territories in map do not imply any political statement.

## Supplementary Figure S6: Geographic distribution of available data for group B *Streptococcus* (GBS) from MLST and virulence genes search

Borders of countries/territories in map do not imply any political statement.

## Supplementary Figure S7: Distribution of group B Streptococcus (GBS) serotypes for maternal colonisation isolates in African subregions (adjusted proportions).


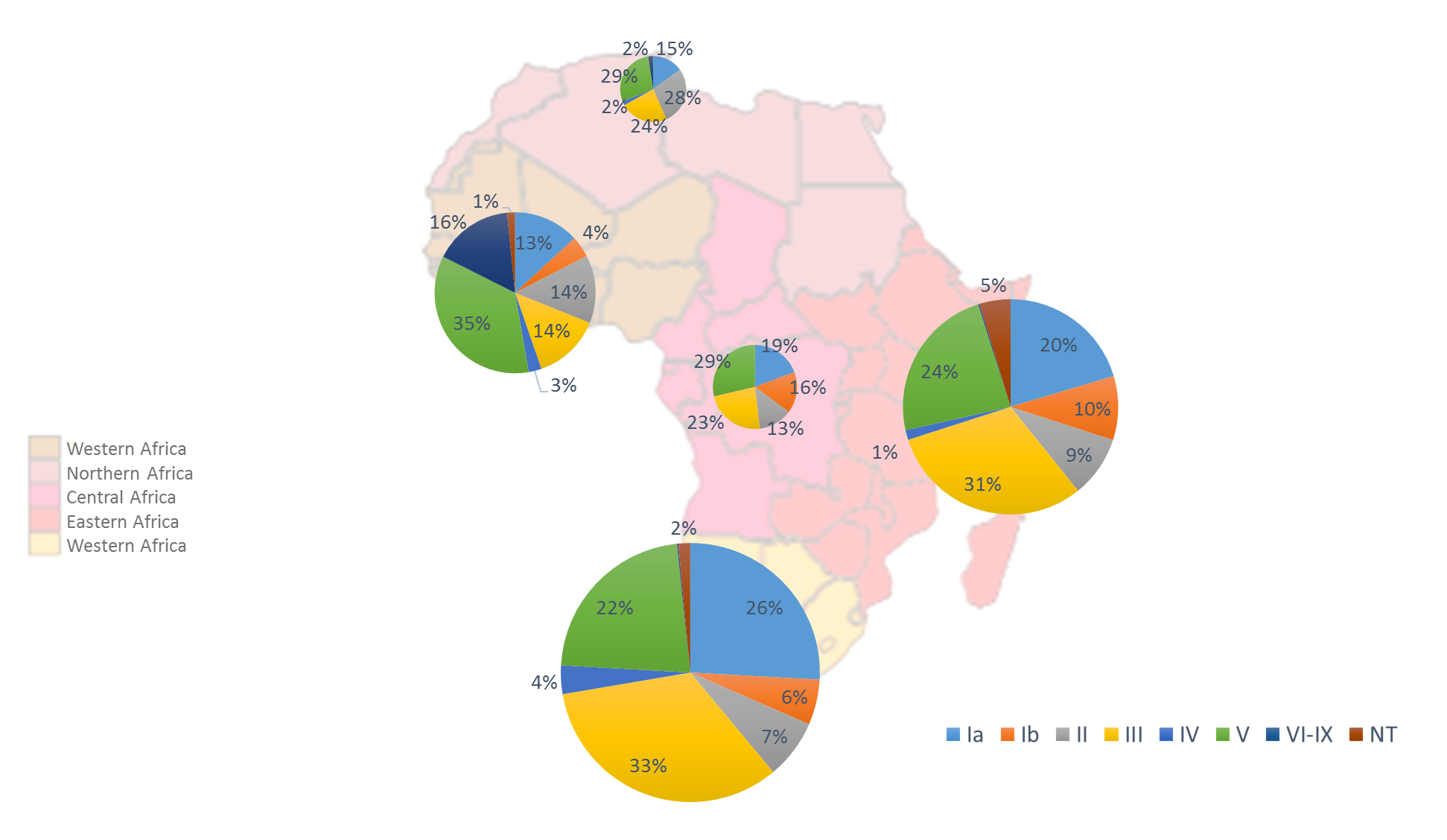


## Supplementary Figure S8: Distribution of group B Streptococcus (GBS) serotypes for maternal colonisation isolates in North America (United States of America and Canada) and Europe (adjusted proportions).


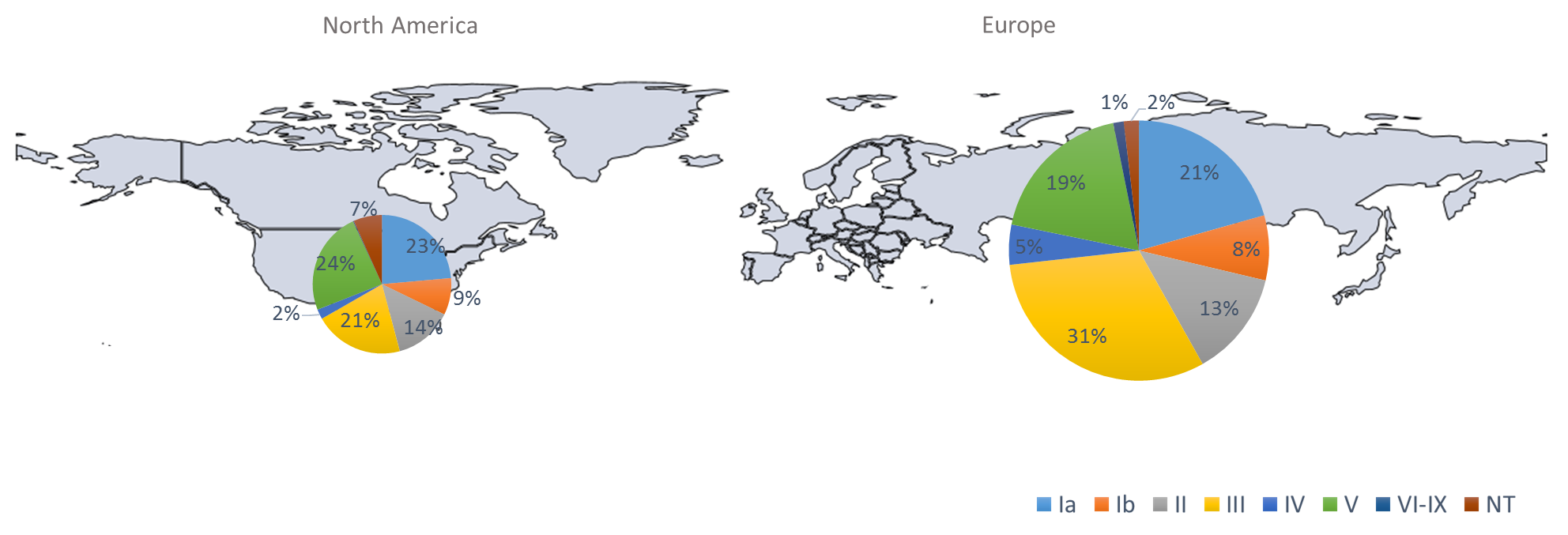


## Supplementary Figure S9: Distribution of group B Streptococcus (GBS) serotypes within isolates from clonal complexes 1, 23, 19, 10, 17 and 12 for maternal colonisation and infant colonisation.

| Maternal colonisation CC | Total samples tested | Total CC | Proportion CC |
| --- | --- | --- | --- |
| CC1 | 2603 | 455 | 17% |
| CC23 | 2603 | 500 | 19% |
| CC19 | 2603 | 576 | 22% |
| CC10 | 2603 | 234 | 9% |
| CC17 | 2603 | 402 | 15% |
| CC12 | 2603 | 111 | 4% |


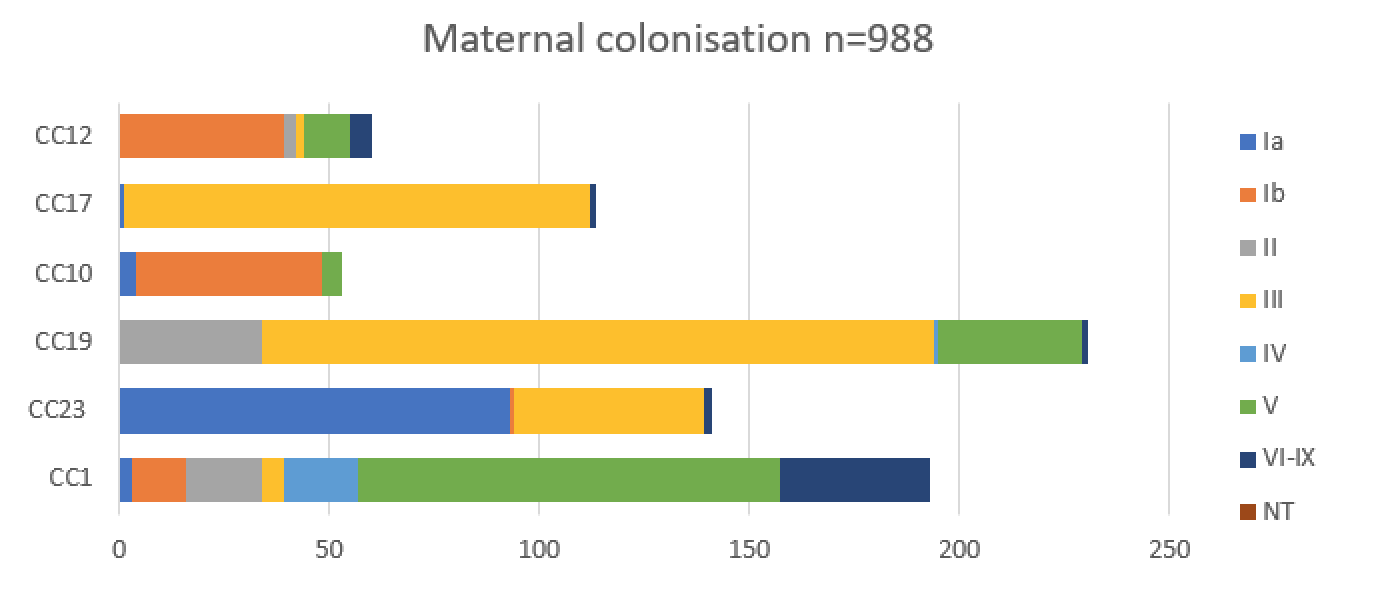


| Infant colonisation CC | Total samples tested | Total CC | Proportion CC |
| --- | --- | --- | --- |
| CC1 | 109 | 15 | 14% |
| CC23 | 109 | 23 | 21% |
| CC19 | 109 | 42 | 39% |
| CC10 | 109 | 8 | 7% |
| CC17 | 109 | 5 | 5% |
| CC12 | 109 | 3 | 3% |

## Supplementary Figure S10: Distribution of group B Streptococcus (GBS) serotypes within isolates with alp1/epsilon, alp2/3, rib, alpha C or none surface protein genes for maternal colonisation and infant colonisation.

| Maternal colonisation surface protein gene | Total samples | Total protein | Proportion |
| --- | --- | --- | --- |
| alp1/epsilon | 906 | 233 | 26% |
| alp2/3 | 906 | 175 | 19% |
| rib | 1047 | 424 | 40% |
| bca/alphaC | 684 | 190 | 28% |
| none | 498 | 9 | 2% |

| Infant colonisation surface protein gene | Total samples | Total protein | Proportion |
| --- | --- | --- | --- |
| alp1/epsilon | 44 | 12 | 27% |
| alp2/3 | 44 | 12 | 27% |
| rib | 44 | 10 | 23% |
| bca/alphaC | 44 | 8 | 18% |
| none | 44 | 0 | 0% |

## Supplementary Figure S11: Distribution of group B Streptococcus (GBS) serotypes within isolates with pilus island 2a, PI-2b and combinations with PI-1 for maternal colonisation.

| Maternal colonisation pilus island | Total samples | Total PI | Proportion PI |
| --- | --- | --- | --- |
| PI-1 | 835 | 0 | 0% |
| PI-2a | 835 | 271 | 32% |
| PI-2b | 835 | 26 | 3% |
| PI-1+PI-2a | 835 | 315 | 38% |
| PI-1+PI-2b | 835 | 223 | 27% |

| Infant colonisation pilus island | Total samples | Total PI | Proportion PI |
| --- | --- | --- | --- |
| PI-1 | 35 | 0 | 0% |
| PI-2a | 35 | 5 | 14% |
| PI-2b | 35 | 3 | 9% |
| PI-1+PI-2a | 35 | 27 | 77% |
| PI-1+PI-2b | 35 | 0 | 0% |

## Supplementary Figure S12: Distribution of group B Streptococcus (GBS) serotypes for maternal invasive GBS disease isolates by regions (adjusted proportions).


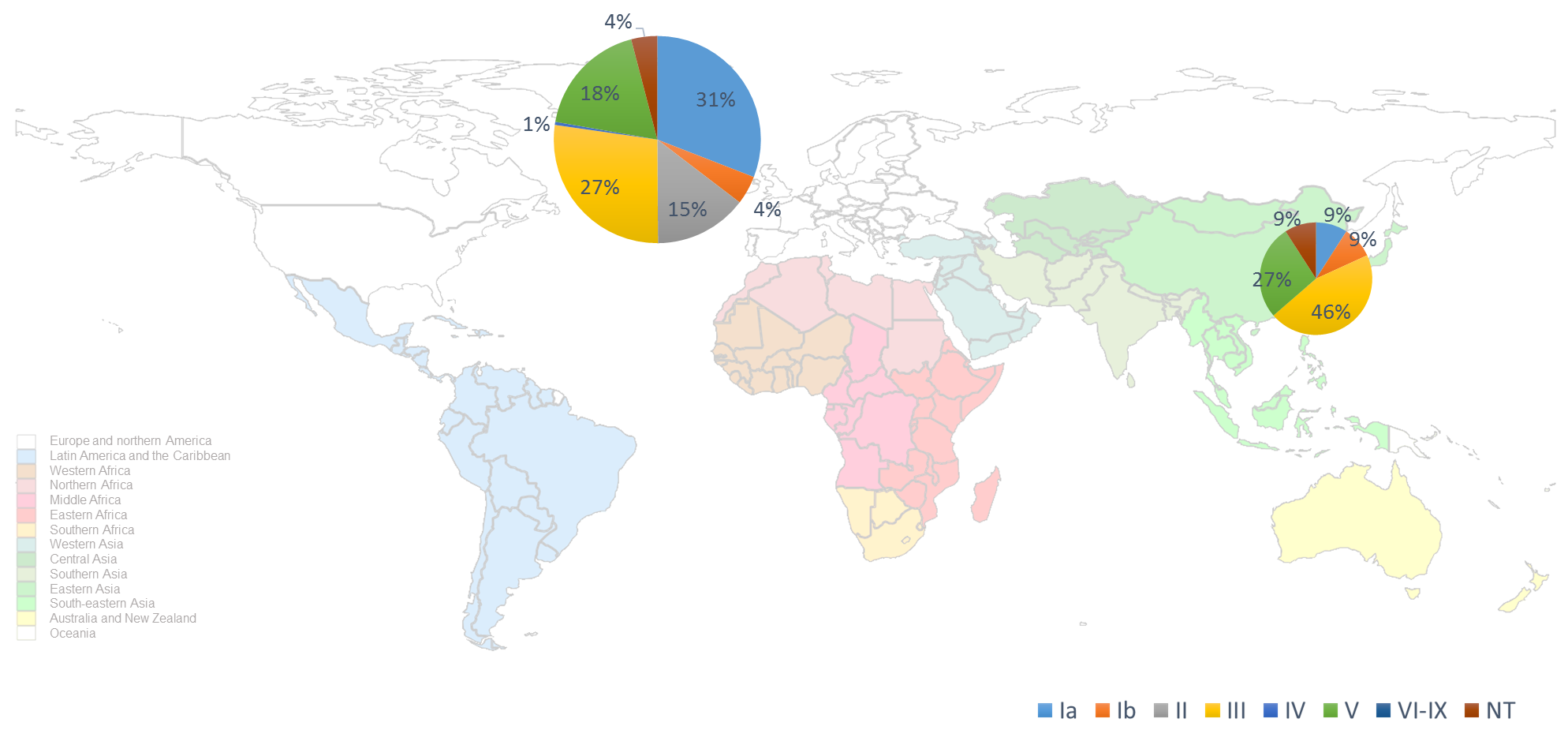


## Supplementary Figure S13: Distribution of group B Streptococcus (GBS) serotypes within isolates from clonal complexes 1, 23, 19, 10, 17 and 12 for maternal invasive, infant invasive and adult invasive.

| Maternal invasive disease CC | Total samples tested | Total CC | Proportion CC |
| --- | --- | --- | --- |
| CC1 | 29 | 3 | 10% |
| CC23 | 29 | 12 | 41% |
| CC19 | 29 | 4 | 14% |
| CC10 | 29 | 0 | 0% |
| CC17 | 29 | 7 | 24% |
| CC12 | 29 | 3 | 10% |

| Infant invasive disease CC | Total samples tested | Total CC | Proportion CC |
| --- | --- | --- | --- |
| CC1 | 2230 | 209 | 9% |
| CC23 | 2230 | 331 | 15% |
| CC19 | 2230 | 401 | 18% |
| CC10 | 2230 | 88 | 4% |
| CC17 | 2230 | 944 | 42% |
| CC12 | 2230 | 67 | 3% |

| Adult invasive disease CC | Total samples tested | Total CC | Proportion CC |
| --- | --- | --- | --- |
| CC1 | 1551 | 577 | 37% |
| CC23 | 1551 | 279 | 18% |
| CC19 | 1551 | 202 | 13% |
| CC10 | 1551 | 274 | 18% |
| CC17 | 1551 | 64 | 4% |
| CC12 | 1551 | 33 | 2% |

## Supplementary Figure S14: Distribution of group B Streptococcus (GBS) serotypes for infant invasive GBS disease and stillbirth isolates in African subregions (adjusted proportions).


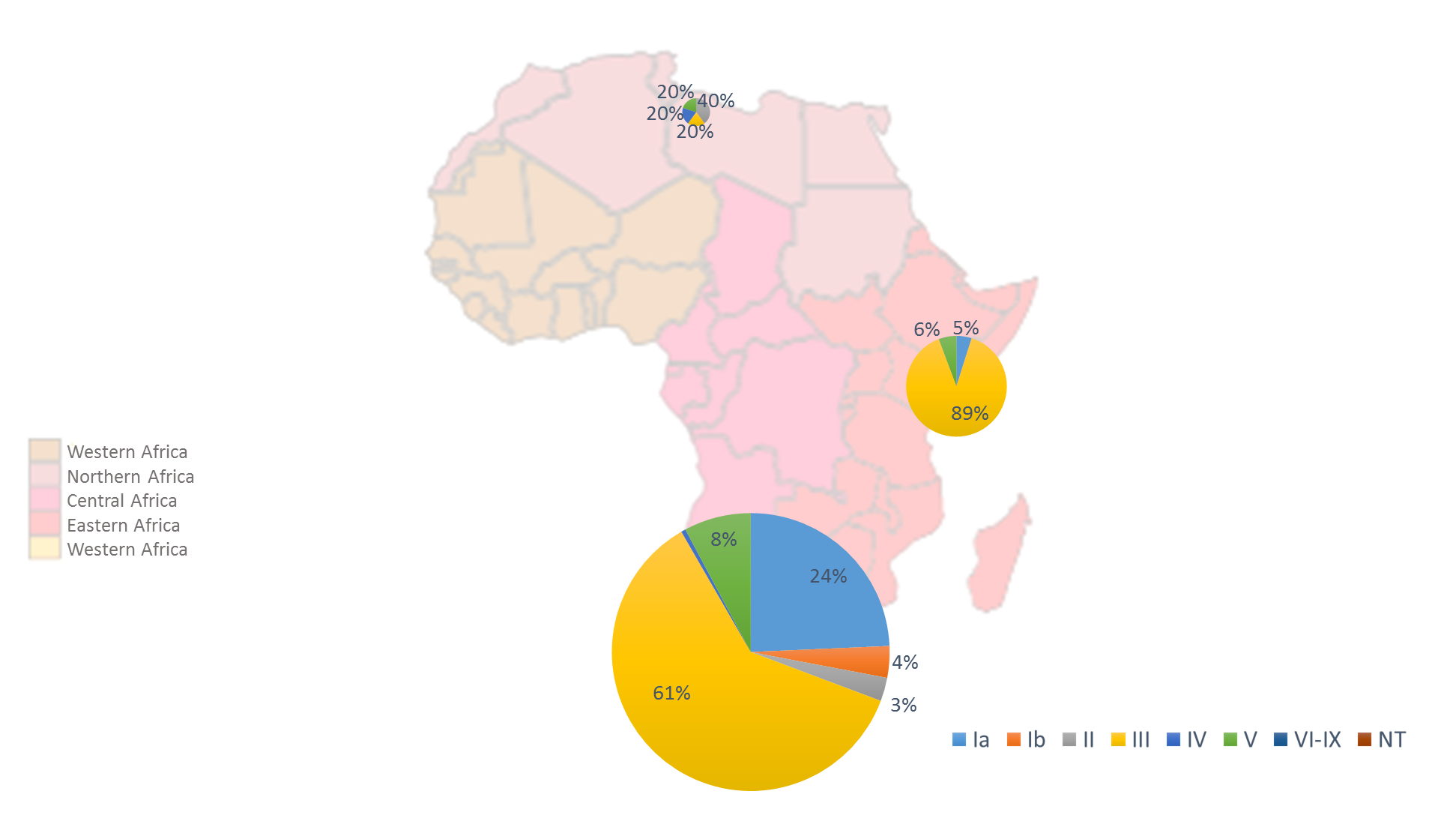


## Supplementary Figure S15: Distribution of group B Streptococcus (GBS) serotypes for infant invasive GBS disease and stillbirths isolates in North America (United States of America and Canada) and Europe (adjusted proportions).


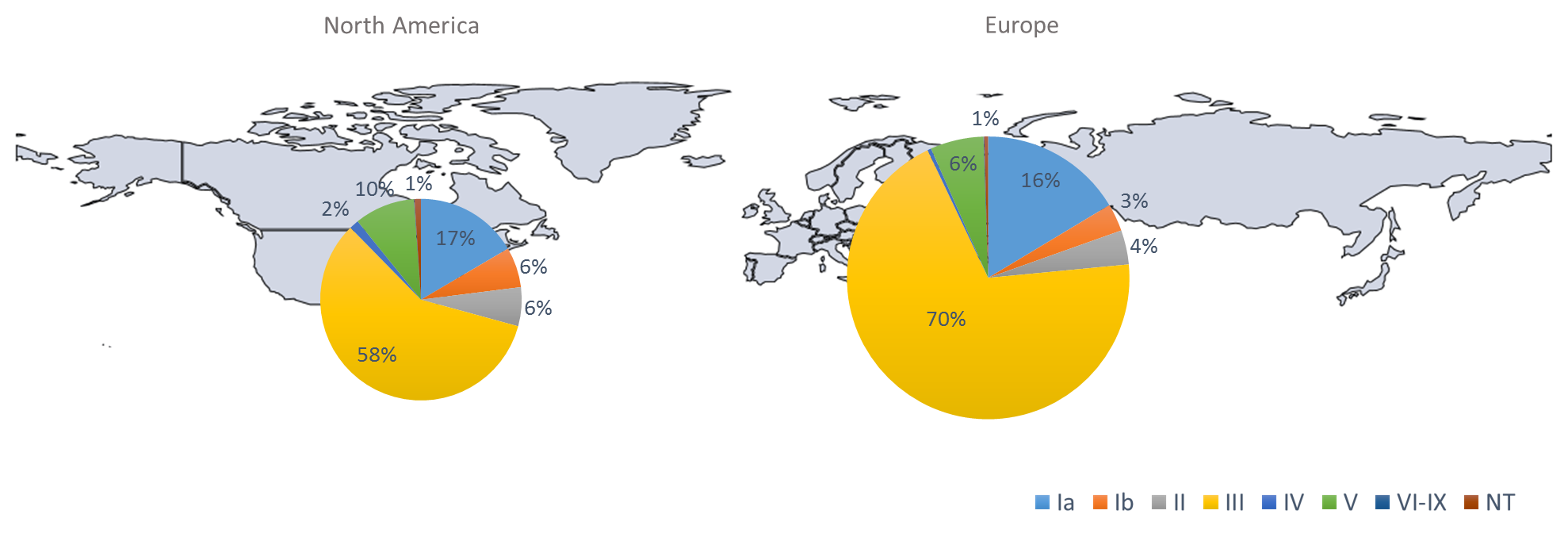


## Supplementary Figure S16: Distribution of group B Streptococcus (GBS) serotypes within isolates with alp1/epsilon, alp2/3, rib, alpha C or none surface protein genes for infant invasive disease and adult invasive disease.

| Infant invasive disease surface protein gene | Total samples | Total protein | Proportion |
| --- | --- | --- | --- |
| alp1/epsilon | 1246 | 210 | 17% |
| alp2 | 888 | 5 | 1% |
| alp3 | 888 | 82 | 9% |
| Rib | 1346 | 731 | 54% |
| bca/alphaC | 1238 | 203 | 16% |
| none | 1138 | 29 | 3% |

| Adult invasive disease surface protein gene | Total samples | Total protein | Proportion |
| --- | --- | --- | --- |
| alp1/epsilon | 1099 | 235 | 21% |
| alp2 | 1099 | 20 | 2% |
| alp3 | 1099 | 315 | 29% |
| Rib | 1099 | 254 | 23% |
| bca/alphaC | 1099 | 270 | 25% |
| none | 1099 | 12 | 1% |

## Supplementary Figure S17: Distribution of group B Streptococcus (GBS) serotypes within isolates with pilus island 2a, PI-2b and combinations with PI-1 for infant invasive disease and adult invasive disease.

| Infant invasive disease pilus island | Total samples | Total PI | Proportion PI |
| --- | --- | --- | --- |
| PI-1 | 1035 | 0 | 0% |
| PI-2a | 1035 | 201 | 19% |
| PI-2b | 1035 | 29 | 3% |
| PI-1+PI-2a | 1035 | 327 | 32% |
| PI-1+PI-2b | 1035 | 478 | 46% |

| Adult invasive disease pilus island | Total samples | Total PI | Proportion PI |
| --- | --- | --- | --- |
| PI-1 | 776 | 0 | 0% |
| PI-2a | 776 | 232 | 30% |
| PI-2b | 776 | 15 | 2% |
| PI-1+PI-2a | 776 | 473 | 61% |
| PI-1+PI-2b | 776 | 60 | 8% |

## Supplementary Figure S18: Plot comparing meta-analysis results for main analysis with all studies (n1=studies/samples) and sensitivity analysis (n2=studies/samples) for maternal colonisation (A), infant invasive disease (B), and invasive disease in elderly (C)

A.

n1=124/17427, n2=94/14825

B.

n1=53/8940, n2=47/6604

C.

n1=11/2525, n2=9/1859

CI: confidence interval

## References

1. Russell NJ, Seale AC, O'Driscoll M, O'Sullivan C, Bianchi-Jassir F, Gonzalez-Guarin J, et al. Maternal Colonization With Group B Streptococcus and Serotype Distribution Worldwide: Systematic Review and Meta-analyses. Clin Infect Dis. 2017;65(suppl_2):S100-s11.

2. Pinto AM, Pereira TA, Alves V, Araujo A, Lage OM. Incidence and serotype characterisation of Streptococcus agalactiae in a Portuguese hospital. Journal of Clinical Pathology. 2018;71(6):508-13.

3. Creti R, Imperi M, Berardi A, Pataracchia M, Recchia S, Alfarone G, et al. Neonatal group B streptococcus infections: Prevention strategies, clinical and microbiologic characteristics in 7 years of surveillance. Pediatric Infectious Disease Journal. 2017;36(3):256-62.

4. Kardos S, Szabo J, Major T, Krasznai Z, Laub K, Tothpal A, et al. Streptococcus agalactiae isolates from pregnancy screening at the university of Debrecen. Acta Microbiologica et Immunologica Hungarica. 2017;64 (Supplement 1):39-40.

5. Teatero S, Ferrieri P, Martin I, Demczuk W, McGeer A, Fittipaldi N. Serotype Distribution, Population Structure, and Antimicrobial Resistance of Group B Streptococcus Strains Recovered from Colonized Pregnant Women. Journal of Clinical Microbiology. 2017;55(2):412-22.

6. Lopez Y, Parra E, Cepas V, Sanfeliu I, Juncosa T, Andreu A, et al. Serotype, virulence profile, antimicrobial resistance and macrolide-resistance determinants in Streptococcus agalactiae isolates in pregnant women and neonates in Catalonia, Spain. Enfermedades Infecciosas y Microbiologia Clinica. 2017.

7. Rojo-Bezares B, Azcona-Gutierrez JM, Martin C, Jareno MS, Torres C, Saenz Y. Streptococcus agalactiae from pregnant women: antibiotic and heavy-metal resistance mechanisms and molecular typing. Epidemiol Infect. 2016;144(15):3205-14.

8. Ko DWH, Zurynski Y, Gilbert GL. Group B streptococcal disease and genotypes in Australian infants Australia: Blackwell Publishing; 2015 [cited 51 (Ko, Gilbert) Centre for Infectious Diseases and Microbiology, Westmead Hospital, Sydney, NSW, Australia]. 8:[808-14]. Available from: <www.blackwell-science.com/jpc>

<http://ovidsp.ovid.com/ovidweb.cgi?T=JS&PAGE=reference&D=emed13&NEWS=N&AN=2015724381>.

9. Kunze M, Zumstein K, Markfeld-Erol F, Elling R, Lander F, Prömpeler H, et al. Comparison of pre- and intrapartum screening of group B streptococci and adherence to screening guidelines: a cohort study. European Journal of Pediatrics. 2015;174(6):827-35.

10. Morozumi M, Chiba N, Igarashi Y, Mitsuhashi N, Wajima T, Iwata S, et al. Direct identification of Streptococcus agalactiae and capsular type by real-time PCR in vaginal swabs from pregnant women. Journal of infection and chemotherapy : official journal of the Japan Society of Chemotherapy. 2015;21(1):34-8.

11. Brigtsen AK, Dedi L, Melby KK, Holberg-Petersen M, Radtke A, Lyng RV, et al. Comparison of PCR and serotyping of Group B Streptococcus in pregnant women: the Oslo GBS-study. Journal of microbiological methods. 2015;108:31-5.

12. Liébana-Martos MDC, Cabrera-Alavargonzalez J, Rodríguez-Granger J, Miranda-Casas C, Sampedro-Martínez A, Gutiérrez-Fernández J, et al. Serotypes and antibiotic resistance patterns in beta-hemolytic Streptococcus agalactiae isolates in colonized mothers and newborns with invasive disease. Enfermedades Infecciosas y Microbiologia Clinica. 2015;33(2):84-8.

13. Meehan M, Cunney R, Cafferkey M. Molecular epidemiology of group B streptococci in Ireland reveals a diverse population with evidence of capsular switching. European Journal of Clinical Microbiology and Infectious Diseases. 2014;33(7):1155-62.

14. Romanik M, Nowosielski K, Porȩba R, Sioma-Markowska U, Martiroisian G, Groborz J. Streptococcus group B serotype distribution in anovaginal isolates of women in term pregnancy. Neuroendocrinology Letters. 2014;35(4):301-5.

15. Fröhlicher S, Reichen G, Müller M, Surbek D, Droz S, Spellerberg B, et al. Serotype distribution and antimicrobial susceptibility of group B streptococci in pregnant women: Results from a Swiss tertiary centre. Swiss Medical Weekly. 2014;144.

16. Kimura K, Matsubara K, Yamamoto G, Shibayama K, Arakawa Y. Active screening of group B Streptococci with reduced penicillin susceptibility and altered serotype distribution isolated from pregnant women in Kobe, Japan. Japanese Journal of Infectious Diseases. 2013;66(2):158-60.

17. Barcaite E, Bartusevicius A, Tameliene R, Maleckiene L, Vitkauskiene A, Nadisauskiene R. Group B streptococcus and Escherichia coli colonization in pregnant women and neonates in Lithuania. International Journal of Gynecology and Obstetrics. 2012;117(1):69-73.

18. Brzychczy-Włoch M, Gosiewski T, Bodaszewska-Lubas M, Adamski P, Heczko PB. Molecular characterization of capsular polysaccharides and surface protein genes in relation to genetic similarity of group B streptococci isolated from Polish pregnant women. Epidemiology and Infection. 2012;140(2):329-36.

19. Kunze M, Ziegler A, Fluegge K, Hentschel R, Proempeler H, Berner R. Colonization, serotypes and transmission rates of group B streptococci in pregnant women and their infants born at a single University Center in Germany. Journal of Perinatal Medicine. 2011;39(4):417-22.

20. Wakimoto H, Wakimoto Y, Yano H, Matsubara K, Miyakawa S, Yoshida A, et al. [Antimicrobial susceptibility and serotype distribution in perinatal group B Streptococcus isolates--a 1999-2009 multicenter study] Japan2011 [cited 85 (Wakimoto) Nagoya City University School of Nursing.]. 2:[155-60]. Available from: <http://ovidsp.ovid.com/ovidweb.cgi?T=JS&PAGE=reference&D=emed11&NEWS=N&AN=21560418>.

21. Cristea V-C, Duta M, Neacsu G. Screening for group B streptococcus: a private laboratory experience. Roumanian archives of microbiology and immunology. 2011;70(2):65-8.

22. Brzychczy-Wloch M, Gosiewski T, Bodaszewska M, Pabian W, Ochonska D, Heczko PB. Distribution of PFGE types of group B streptococci originating from Polish pregnant women in relation to capsular polysaccharides and surface protein genes: Blackwell Publishing Ltd; 2010 [cited 16 (Brzychczy-Wloch, Gosiewski, Bodaszewska, Pabian, Ochonska, Heczko) CracowPoland]. S499]. Available from: <http://ovidsp.ovid.com/ovidweb.cgi?T=JS&PAGE=reference&D=emed9&NEWS=N&AN=70196304>.

23. Gonzalez E, Cabrera J, Rodriguez-Granger J, Serrano M, Navarro J. Distribution of serotypes of clinical group B (GBS) streptococci newborn and pregnant women in ;Andalucia (Spain). EUROPEAN WORKSHOP: Current Insights into Group B Streptococcal Diseases; Larnaca, Cyprus2009.

24. Liebana C, Cabrera J, Moreno E, Rodriguez-Granger J, Navarro-Mari JM, Molina FS. Comparison of two capsular serotyping methods among isolates of streptococcus agalactiae from pregnant women: Informa Healthcare; 2010 [cited 23 (Liebana, Cabrera, Moreno, Rodriguez-Granger, Navarro-Mari, Molina) Microbiology Service, Virgen de las Nieves Hospital, Granada, Spain]. 555-6]. Available from: <http://ovidsp.ovid.com/ovidweb.cgi?T=JS&PAGE=reference&D=emed9&NEWS=N&AN=70201289>.

25. El Aila NA, Tency I, Claeys G, Saerens B, De Backer E, Temmerman M, et al. Genotyping of Streptococcus agalactiae (group B streptococci) isolated from vaginal and rectal swabs of women at 35-37 weeks of pregnancy. BMC Infectious Diseases. 2009;9:153.

26. Van Der Mee-Marquet N, Jouannet C, Domelier AS, Arnault L, Lartigue MF, Quentin R. Genetic diversity of Streptococcus agalactiae strains and density of vaginal carriage United Kingdom: Society for General Microbiology (Basingstoke Road, Spencers Wood, Reading, Berkshire RG7 1AE, United Kingdom); 2009 [cited 58 (Van Der Mee-Marquet, Domelier, Lartigue, Quentin) Universite Francois-Rabelais, IFR 136, Faculte de Medecine, EA 3854 Bacteries et Risque Materno-Foetal, Tours, France]. 2:[169-73]. Available from: <http://jmm.sgmjournals.org/cgi/reprint/58/2/169>

<http://ovidsp.ovid.com/ovidweb.cgi?T=JS&PAGE=reference&D=emed9&NEWS=N&AN=2009073172>.

27. Van Elzakker E, Yahiaoui R, Visser C, Oostvogel P, Muller A, Ho YR, et al. Epidemiology of and prenatal molecular distinction between invasive and colonizing group B streptococci in the Netherlands and Taiwan. European Journal of Clinical Microbiology and Infectious Diseases. 2009;28(8):921-8.

28. Wolski B, Zegarska J, Adamczak R, Szymanski W, Kaczmarek A, Dorota M. Serotype I, II, III distribution and antimicrobial susceptibility of Streptococcus agalactiae among Polish delivering women: Elsevier Ireland Ltd; 2009 [cited 107 (Wolski, Zegarska, Adamczak, Szymanski) Women's Diseases and Gynecological Oncology Department, Collegium Medicum in Bydgoszcz, Nicolaus Copernicus University, Torun, Poland]. S575]. Available from: <http://ovidsp.ovid.com/ovidweb.cgi?T=JS&PAGE=reference&D=emed9&NEWS=N&AN=70231032>.

29. Usein CR, Petrini A, Georgescu R, Grigore L, Strǎuţ M, Ungureanu V. Group B streptococcus colonization of Romanian women: phenotypic traits of isolates from vaginal swabs. Roumanian archives of microbiology and immunology. 2009;68(4):235-9.

30. Codruta-Romanita U, Mariana-Silvia C, Vasilica U, Maria G, Anca P, Monica S. Genetic heterogeneity of group b streptococci isolated from Romanian pregnant women. Roum Arch Microbiol Immunol. 2009;68(2):83-8.

31. Savoia D, Gottimer C, Crocilla C, Zucca M. Streptococcus agalactiae in pregnant women: Phenotypic and genotypic characters United Kingdom: W.B. Saunders Ltd; 2008 [cited 56 (Savoia, Gottimer, Zucca) Department of Clinical and Biological Sciences, University of Turin, at S. Luigi Gonzaga Hospital, Regione Gonzole 10, 10043 Orbassano, TO, Italy]. 2:[120-5]. Available from: <http://ovidsp.ovid.com/ovidweb.cgi?T=JS&PAGE=reference&D=emed8&NEWS=N&AN=2008057105>.

32. Håkansson S, Axemo P, Bremme K, Bryngelsson AL, Wallin MC, Ekström CM, et al. Group B streptococcal carriage in Sweden: A national study on risk factors for mother and infant colonisation. Acta Obstetricia et Gynecologica Scandinavica. 2008;87(1):50-8.

33. Martins ER, Pessanha MA, Ramirez M, Melo-Cristino J, Lopes P, Calheiros I, et al. Analysis of group B streptococcal isolates from infants and pregnant women in Portugal revealing two lineages with enhanced invasiveness United States: American Society for Microbiology; 2007 [cited 45 (Martins, Pessanha, Ramirez, Melo-Cristino) Instituto de Microbiologia, Faculdade de Medicina, Universidade de Lisboa, Lisbon, Portugal]. 10:[3224-9]. Available from: <http://ovidsp.ovid.com/ovidweb.cgi?T=JS&PAGE=reference&D=emed8&NEWS=N&AN=2007505096>.

34. Taylor KL. A study of group B streptococcus in Brisbane : the epidemiology, detection by PCR assay and serovar prevalence [Masters by Research]2006.

35. Lamy M-C, Dramsi S, Billoet A, Reglier-Poupet H, Tazi A, Raymond J, et al. Rapid detection of the "highly virulent" group B Streptococcus ST-17 clone. Microbes and infection / Institut Pasteur. 2006;8(7):1714-22.

36. Marchaim D, Efrati S, Melamed R, Gortzak-Uzan L, Riesenberg K, Zaidenstein R, et al. Clonal variability of group B Streptococcus among different groups of carriers in southern Israel. European Journal of Clinical Microbiology and Infectious Diseases. 2006;25(7):443-8.

37. Jones N, Oliver K, Jones Y, Haines A, Crook D. Carriage of group B streptococcus in pregnant women from Oxford, UK. Journal of Clinical Pathology. 2006;59(4):363-6.

38. Bisharat N, Jones N, Marchaim D, Block C, Harding RM, Yagupsky P, et al. Population structure of group B streptococcus from a low-incidence region for invasive neonatal disease. Microbiology (Reading, England). 2005;151(Pt 6):1875-81.

39. Motlová J, Straková L, Ur̂bášková P, Sak P, Sever T. Vaginal & rectal carriage of Streptococcus agalactiae in the Czech Republic: Incidence, serotypes distribution & susceptibililty to antibiotics. Indian Journal of Medical Research, Supplement. 2004;119(MAY):84-7.

40. Whitney CG, Daly S, Limpongsanurak S, Festin MR, Thinn KK, Chipato T, et al. The international infections in pregnancy study: Group B streptococcal colonization in pregnant women. Journal of Maternal-Fetal and Neonatal Medicine. 2004;15(4):267-74.

41. von Both U, Ruess M, Mueller U, Fluegge K, Sander A, Berner R. A serotype V clone is predominant among erythromycin-resistant Streptococcus agalactiae isolates in a southwestern region of Germany. Journal of clinical microbiology. 2003;41(5):2166-9.

42. Tsolia M, Psoma M, Gavrili S, Petrochilou V, Michalas S, Legakis N, et al. Group B streptococcus colonization of Greek pregnant women and neonates: Prevalence, risk factors and serotypes. Clinical Microbiology and Infection. 2003;9(8):832-8.

43. Dore N, Bennett D, Kaliszer M, Cafferkey M, Smyth CJ. Molecular epidemiology of group B streptococci in Ireland: Associations between serotype, invasive status and presence of genes encoding putative virulence factors United Kingdom: Cambridge University Press; 2003 [cited 131 (Dore, Bennett, Cafferkey) Irish Meningoc./Meningitis Ref. Lab., The Children's University Hospital, Temple Street, Dublin 1, Ireland]. 2:[823-33]. Available from: <http://ovidsp.ovid.com/ovidweb.cgi?T=JS&PAGE=reference&D=emed6&NEWS=N&AN=2003434072>.

44. Perez-Ruiz M, Rodriguez-Granger JM, Bautista-Marin MF, Romero-Noguera J, Rosa-Fraile M. Genetic diversity of Streptococcus agalactiae strains colonizing the same pregnant woman. Epidemiology and infection. 2004;132(2):375-8.

45. Croak A, Abate G, Goodrum K, Modrzakowski M. Predominance of serotype V and frequency of erythromycin resistance in Streptococcus agalactiae in Ohio. American Journal of Obstetrics and Gynecology. 2003;188(5):1148-50.

46. Terakubo S, Ichiman Y, Takemura H, Yamamoto H, Shimada J, Nakashima H. Serotypes and antibody levels of group B streptococci in pregnant women. Kansenshogaku zasshi The Journal of the Japanese Association for Infectious Diseases. 2003;77(3):121-6.

47. Matsubara K, Katayama K, Baba K, Nigami H, Harigaya H, Sugiyama M. Seroepidemiologic studies of serotype VIII group B Streptococcus in Japan. Journal of Infectious Diseases. 2002;186(6):855-8.

48. Grimwood K, Stone PR, Gosling IA, Green R, Darlow BA, Lennon DR, et al. Late antenatal carriage of group B Streptococcus by New Zealand women. Australian and New Zealand Journal of Obstetrics and Gynaecology. 2002;42(2):182-6.

49. Dele Davies H, Adair C, McGeer A, Ma D, Robertson S, Mucenski M, et al. Antibodies to capsular polysaccharides of group B Streptococcus in pregnant Canadian women: Relationship to colonization status and infection in the neonate. Journal of Infectious Diseases. 2001;184(3):285-91.

50. Berg S, Trollfors B, Lagergard T, Zackrisson G, Claesson BA. Serotypes and clinical manifestations of group B streptococcal infections in western Sweden. Clinical Microbiology and Infection. 2000;6(1):9-13.

51. Campbell JR, Hillier SL, Krohn MA, Ferrieri P, Zaleznik DF, Baker CJ. Group B streptococcal colonization and serotype-specific immunity in pregnant women at delivery. Obstetrics and Gynecology. 2000;96(4):498-503.

52. Lee HT, Kim SY, Park PW, Ahn JY, Kim KH, Seo JY, et al. Detection and genomic analysis of genital group B streptococcus in pregnant Korean women. J Obstet Gynaecol Res. 2019;45(1):69-77.

53. Wang X, Cao X, Li S, Ou Q, Lin D, Yao Z, et al. Phenotypic and molecular characterization of Streptococcus agalactiae colonized in Chinese pregnant women: predominance of ST19/III and ST17/III. Research in Microbiology. 2018;169(2):101-7.

54. Ji W, Zhang L, Guo Z, Xie S, Yang W, Chen J, et al. Colonization prevalence and antibiotic susceptibility of Group B Streptococcus in pregnant women over a 6-year period in Dongguan, China. PLoS ONE. 2017;12 (8) (no pagination)(e0183083).

55. Yan Y, Hu H, Lu T, Fan H, Hu Y, Li G, et al. Investigation of serotype distribution and resistance genes profile in group B Streptococcus isolated from pregnant women: a Chinese multicenter cohort study. Apmis. 2016;124(9):794-9.

56. Wang P, Tong JJ, Ma XH, Song FL, Fan L, Guo CM, et al. Serotypes, antibiotic susceptibilities, and multi-locus sequence type profiles of Streptococcus agalactiae isolates circulating in Beijing, China United States: Public Library of Science; 2015 [cited 10 (Wang, Tong, Shi, Yu, Yao, Yang) Key Laboratory of Major Diseases in Children and National Key Discipline of Pediatrics, Beijing Children's Hospital, Capital Medical University, Beijing 100045, China]. 3:[Available from: <http://www.plosone.org/article/fetchObject.action?uri=info:doi/10.1371/journal.pone.0120035&representation=PDF>

<http://ovidsp.ovid.com/ovidweb.cgi?T=JS&PAGE=reference&D=emed13&NEWS=N&AN=2015838651>.

57. Lu B, Wang D, Zhou H, Zhu F, Li D, Zhang S, et al. Distribution of pilus islands and alpha-like protein genes of group B Streptococcus colonized in pregnant women in Beijing, China. Eur J Clin Microbiol Infect Dis. 2015;34(6):1173-9.

58. Lu B, Li D, Cui Y, Sui W, Huang L, Lu X. Epidemiology of Group B streptococcus isolated from pregnant women in Beijing, China. Clinical Microbiology and Infection. 2014;20(6):O370-O3.

59. Lee HR, Song SH, Kim HB, Park KU, Song J. A rapid genotyping test for the simultaneous detection and subtyping of group B streptococci: The frequency of molecular subtypes of group B streptococci in Korea. European Journal of Clinical Microbiology and Infectious Diseases. 2010;29(10):1287-90.

60. Seo YS, Srinivasan U, Oh KY, Shin JH, Chae JD, Kim MY, et al. Changing molecular epidemiology of group B streptococcus in Korea. Journal of Korean medical science. 2010;25(6):817-23.

61. Lee BK, Song YR, Kim MY, Yang JH, Shin JH, Seo YS, et al. Epidemiology of group B streptococcus in Korean pregnant women. Epidemiology and Infection. 2010;138(2):292-8.

62. Hong JS, Choi CW, Park KU, Kim SN, Lee HJ, Lee HR, et al. Genital group B streptococcus carrier rate and serotype distribution in Korean pregnant women: Implications for group B streptococcal disease in Korean neonates. Journal of Perinatal Medicine. 2010;38(4):373-7.

63. Oh CE, Jang HO, Kim NH, Lee J, Choi EH, Lee HJ. Molecular serotyping of Group B streptococcus isolated from the pregnant women by polymerase chain reaction and sequence analysis. Korean Journal of Pediatric Infectious Diseases. 2009;16(1):47-53.

64. Shen AD, Yang YY, Schollin J. Serotype distribution and antimicrobial susceptibility profiles of group B streptococcus strains from pregnant women in Beijing, 1994-99. Prenatal and Neonatal Medicine. 2000;5(4):230-5.

65. Shen A, Zhu Y, Zhang G, Yang Y, Jiang Z. Experimental study on distribution of serotypes and antimicrobial patterns of group B streptococcus strains. Chinese medical journal. 1998;111(7):615-8.

66. Uh Y, Jang IH, Yoon KJ, Lee CH, Kwon JY, Kim MC. Colonization rates and serotypes of group B streptococci isolated from pregnant women in a Korean tertiary hospital. European Journal of Clinical Microbiology and Infectious Diseases. 1997;16(10):753-6.

67. Botelho ACN, Oliveira JG, Damasco AP, Santos KTB, Ferreira AFM, Rocha GT, et al. Streptococcus agalactiae carriage among pregnant women living in Rio de Janeiro, Brazil, over a period of eight years. PLoS ONE. 2018;13 (5) (no pagination)(e0196925).

68. Oviedo P, Pegels E, Laczeski M, Quiroga M, Vergara M. Phenotypic and genotypic characterization of Streptococcus agalactiae in pregnant women. First study in a province of Argentina. Brazilian Journal of Microbiology. 2013;44(1):253-8.

69. Soares GCT, Alviano DS, Santos GS, Alviano CS, Mattos-Guaraldi AL, Nagao PE. Prevalence of group B Streptococcus serotypes III and V in pregnant women of Rio de Janeiro, Brazil. Brazilian Journal of Microbiology. 2013;44(3):869-72.

70. Palmeiro JK, Dalla-Costa LM, Fracalanzza SE, Botelho AC, da Silva Nogueira K, Scheffer MC, et al. Phenotypic and genotypic characterization of group B streptococcal isolates in southern Brazil. Journal of Clinical Microbiology. 2010;48(12):4397-403.

71. Simoes JA, Alves VMN, Fracalanzza SEL, de Camargo RPS, Mathias L, Milanez HMBP, et al. Phenotypical characteristics of group B streptococcus in parturients. Brazilian Journal of Infectious Diseases. 2007;11(2):261-6.

72. Gonzalez PA OZ, Madrigal de Leon HG, Corzo CMT, Flores HP. . Colonizacion por streptococcus grupo b en mujeres embarazadas de un centro de atencion primaria de la Ciudad de Mexico Mrch Med Fam. 2004;6:44-7.

73. Gonzalez Pedraza Aviles A, Ortiz Zaragoza MC, Mota Vazquez R. Serotypes and antimicrobial susceptibility of group B Streptococcus from pregnant women in Mexico. Revista Latinoamericana de Microbiologia. 2002;44(3-4):133-6.

74. Ocampo-Torres M, Sánchez-Pérez HJ, Nazar-Beutelspacher A, Castro-Ramírez AE, Cordero-Ocampo B. Factors associated with group B Streptococcus colonization in pregnant women of Los Altos, Chiapas, Mexico. Salud Publica de Mexico. 2000;42(5):413-21.

75. Solorzano-Santos F E-AG, Conde-Gonzalez CJ, Calderon-Jaimes E, Arredondo-Garcia JL, Beltran Zuniga M. CervicoVaginal Infection with Group B streptococci among Pregnant Mexican Women The Journal of Infectious Diseases 1989;159(5):1003-4.

76. Benchetrit LC, Francalanzza S.L., Peregrino H., Camelo A., Sanches A. Carriage of Streptococcus agalactiae in women and neonates and distribution of serological types: A Study in brazil Journal of Clinical Microbiology. 1982;15(5):787.

77. Moraleda C, Benmessaoud R, Esteban J, Lopez Y, Alami H, Barkat A, et al. Prevalence, antimicrobial resistance and serotype distribution of group B streptococcus isolated among pregnant women and newborns in Rabat, Morocco. Journal of Medical Microbiology. 2018;67(5):652-61.

78. Bergal A, Loucif L, Benouareth DE, Bentorki AA, Abat C, Rolain JM. Molecular epidemiology and distribution of serotypes, genotypes, and antibiotic resistance genes of Streptococcus agalactiae clinical isolates from Guelma, Algeria and Marseille, France. Eur J Clin Microbiol Infect Dis. 2015;34(12):2339-48.

79. Benbachir M, El Mdaghri N, Lahlou D, Mesbahi M. Etude du portage de Streptococcus agalactiae et de Listeria monocyto genes chez la femme marocaine. Medecine et Maladies Infectieuses. 1983;13(12):793-7.

80. Suhaimi MES, Desa MNM, Eskandarian N, Pillay SG, Ismail Z, Neela VK, et al. Characterization of a Group B Streptococcus infection based on the demographics, serotypes, antimicrobial susceptibility and genotypes of selected isolates from sterile and non-sterile isolation sites in three major hospitals in Malaysia. Journal of Infection and Public Health. 2017;10(1):14-21.

81. Eskandarian N, Ismail Z, Neela V, van Belkum A, Desa MN, Amin Nordin S. Antimicrobial susceptibility profiles, serotype distribution and virulence determinants among invasive, non-invasive and colonizing Streptococcus agalactiae (group B streptococcus) from Malaysian patients. European Journal of Clinical Microbiology & Infectious Diseases. 2015;34(3):579-84.

82. Turner C, Turner P, Po L, Maner N, De Zoysa A, Afshar B, et al. Group B streptococcal carriage, serotype distribution and antibiotic susceptibilities in pregnant women at the time of delivery in a refugee population on the Thai-Myanmar border. BMC Infectious Diseases. 2012;12.

83. Dhanoa A, Karunakaran R, Puthucheary SD. Serotype distribution and antibiotic susceptibility of group B streptococci in pregnant women. Epidemiology and Infection. 2010;138(7):979-81.

84. Kumar P. PGIMER india GBS 2016.

85. Saha SK, Ahmed ZB, Modak JK, Naziat H, Saha S, Uddin MA, et al. Group B streptococcus among pregnant women and newborns in Mirzapur, Bangladesh: Colonization, vertical transmission, and serotype distribution. Journal of Clinical Microbiology. 2017;55(8):2406-12.

86. Chaudhary M, Rench MA, Baker CJ, Singh P, Hans C, Edwards MS. Group B Streptococcal Colonization among Pregnant Women in Delhi, India. Pediatric Infectious Disease Journal. 2017;36(7):665-9.

87. Chan GJ, Modak JK, Mahmud AA, Baqui AH, Black RE, Saha SK. Maternal and neonatal colonization in Bangladesh: Prevalences, etiologies and risk factors. Journal of Perinatology. 2013;33(12):971-6.

88. Mani V, Jadhav M, Sivadasan K, Thangavelu CP, Rachel M, Prabha J. Maternal and neonatal colonization with group B Streptococcus and neonatal outcome. Indian Pediatrics. 1984;21(5):357-63.

89. Africa CWJ, Kaambo E. Group B Streptococcus Serotypes in Pregnant Women From the Western Cape Region of South Africa. Frontiers in public health. 2018;6:356-.

90. Medugu N, Iregbu KC, Parker RE, Plemmons J, Singh P, Audu LI, et al. Group B streptococcal colonization and transmission dynamics in pregnant women and their newborns in Nigeria: implications for prevention strategies. Clinical Microbiology and Infection. 2017;23(9):673.e9-.e16.

91. Slotved HC, Dayie N, Banini JAN, Frimodt-Moller N. Carriage and serotype distribution of Streptococcus agalactiae in third trimester pregnancy in southern Ghana. BMC Pregnancy & Childbirth. 2017;17(1):238.

92. Le Doare K. JS, Darboe S., Warburton F., Gorringe A., Heath P.T., Kampmann, B. . Risk Factors for Group B Streptococcus colonisation and disease in Gambian Women and their infants Journal of infection 2016(S0163-4453(15)00406-5).

93. Seale AC, Koech AC, Sheppard AE, Barsosio HC, Langat J, Anyango E, et al. Maternal colonization with Streptococcus agalactiae and associated stillbirth and neonatal disease in coastal Kenya. Nature Microbiology. 2016;1(7):16067.

94. Madrid L, Maculuve SA, Vilajeliu A, Saez E, Massora S, Cossa A, et al. Maternal Carriage of Group B Streptococcus and Escherichia coli in a District Hospital in Mozambique. Pediatr Infect Dis J. 2018;37(11):1145-53.

95. Dangor Y, Said M, Kwatra G, Madhi S, Mbelle N, Ismail F. Prevalence and characterization of group B streptococcus among pregnant women at a tertiary hospital in South Africa. International Journal of Infectious Diseases. 2016;45:221-2.

96. Belard S, Toepfner N, Capan-Melser M, Mombo-Ngoma G, Zoleko-Manego R, Groger M, et al. Streptococcus agalactiae Serotype Distribution and Antimicrobial Susceptibility in Pregnant Women in Gabon, Central Africa. Scientific Reports. 2015;5:17281.

97. Vinnemeier C.D.; Brust P. O-DE, Sarpong N., Sarfo E.Y., Bio Y., Rolling T., Dekker D., Adu-Sarkodie Y., Eberhardt K.A., May J., Cramer, J.P. Group B Streptococci Serotype distribution in pregnant women in Ghana: Assessement of potential coverage through future vaccines. Tropical Medicine and International Health. 2015;20(11):1516-24.

98. Chukwu MO, Mavenyengwa RT, Monyama CM, Bolukaoto JY, Lebelo SL, Maloba MR, et al. Antigenic distribution of Streptococcus agalactiae isolates from pregnant women at Garankuwa hospital - South Africa. Germs. 2015;5(4):125-33.

99. Kwatra G, Adrian PV, Shiri T, Buchmann EJ, Cutland CL, Madhi SA. Serotype-specific acquisition and loss of group B Streptococcus recto-vaginal colonization in late pregnancy. PLoS ONE. 2014;9(6).

100. Gray KJ, Kafulafula G, Matemba M, Kamdolozi M, Membe G, French N. Group B streptococcus and HIV infection in pregnant women, Malawi, 2008-2010 United States: Centers for Disease Control and Prevention (CDC); 2011 [cited 17 (Gray, Kafulafula, Kamdolozi, Membe) College of Medicine, Blantyre, Malawi]. 10:[1932-5]. Available from: <http://wwwnc.cdc.gov/eid/article/17/10/pdfs/10-2008.pdf>

<http://ovidsp.ovid.com/ovidweb.cgi?T=JS&PAGE=reference&D=emed13&NEWS=N&AN=2011540366>.

101. Madzivhandila M, Adrian PV, Cutland CL, Kuwanda L, Schrag SJ, Madhi SA. Serotype distribution and invasive potential of group B streptococcus isolates causing disease in infants and colonizing maternal-newborn dyads. PLoS ONE. 2011;6(3).

102. Brochet M, Couvé E, Bercion R, Sire J-M, Glaser P. Population Structure of Human Isolates of <em>Streptococcus agalactiae</em> from Dakar and Bangui. Journal of Clinical Microbiology. 2009;47(3):800-3.

103. Moyo SR, Maeland JA, Bergh K. Typing of human isolates of Streptococcus agalactiae (group B streptococcus, GBS) strains from Zimbabwe. Journal of Medical Microbiology. 2002;51(7):595-600.

104. Moyo SR, Mudzori J, Tswana SA, Maeland JA. Prevalence, capsular type distribution, anthropometric and obstetric factors of group B Streptococcus (Streptococcus agalactiae) colonization in pregnancy. The Central African journal of medicine. 2000;46(5):115-20.

105. Suara RO, Adegbola RA, Baker CJ, Secka O, Mulholland EK, Greenwood BM. Carriage of group B streptococci in pregnant Gambian mothers and their infants. Journal of Infectious Diseases. 1994;170(5):1316-9.

106. Cutland CL. V98_28OBTP. 2016.

107. Khodaei F, Najafi M, Hasani A, Kalantar E, Sharifi E, Amini A, et al. Pilus-encoding islets in S. agalactiae and its association with antibacterial resistance and serotype distribution. Microbial Pathogenesis. 2018;116:189-94.

108. bornasi h, ghaznavi-Rad e, fard-mousavi n, zand s, abtahi h. Antibiotic resistance profile and capsular serotyping of Streptococcus agalactiae isolated from pregnant women between 35 to 37 weeks of pregnancy. koomesh. 2016;17(2):352-7.

109. Sadeh M, Firouzi R, Derakhshandeh A, Khalili MB, Kong F, Kudinha T. Molecular characterization of streptococcus agalactiae isolates from pregnant and non-pregnant women at yazd university hospital, Iran. Jundishapur Journal of Microbiology. 2016;9(2).

110. Beigverdi R, Jabalameli F, Mirsalehian A, Hantoushzadeh S, Boroumandi S, Taherikalani M, et al. Virulence factors, antimicrobial susceptibility and molecular characterization of streptococcus agalactiae isolated from pregnant women Hungary: Akademiai Kiado Rt.; 2014 [cited 61 (Beigverdi, Jabalameli, Mirsalehian, Emaneini) Department of Microbiology, School of Medicine, Tehran University of Medical Sciences, Tehran, Iran, Islamic Republic of]. 4:[425-34]. Available from: <http://www.akademiai.com> <http://ovidsp.ovid.com/ovidweb.cgi?T=JS&PAGE=reference&D=emed13&NEWS=N&AN=2015986252>.

111. Udo EE, Boswihi SS, Al-Sweih N. Genotypes and virulence genes in group B streptococcus isolated in the maternity hospital, Kuwait. Medical Principles and Practice. 2013;22(5):453-7.

112. Jannati E, Roshani M, Shahram H, Arzanlou M. Antibiotic resistance pattern and serotype distribution of Streptococcus agalactiae isolated from pregnant women, Ardabil, Iran: Elsevier; 2011 [cited 15 (Jannati) Young Researcher Club, Islamic Azad University, Ardabil Branch, Ardabil, Iran, Islamic Republic of]. S53]. Available from: <http://ovidsp.ovid.com/ovidweb.cgi?T=JS&PAGE=reference&D=emed10&NEWS=N&AN=70496609>.

113. Seoud MN, A.H., Zalloua P., Boghossian N., Ezeddine J., Fakhoury H., Abboud J., Melki I., Araj G., Nacouzi G., Sanyoura M., Yunis K. Prenatal and neonatal group b streptococcus screening and serotyping in Lebanon: incidence and implications. Acta Obstetrica et Gynecologica 2010;89:399-403.

114. Yenişehirli G, Bulut Y, Demirtürk F, Çalişkan AC. Antimicrobial susceptibilities and serotype distribution of Streptococcus agalactiae strains isolated from pregnant women. Mikrobiyoloji Bulteni. 2006;40(3):155-60.

115. Al-Sweih N, Hammoud M, Al-Shimmiri M, Jamal M, Neil L, Rotimi V. Serotype distribution and mother-to-baby transmission rate of Streptococcus agalactiae among expectant mothers in Kuwait. Archives of Gynecology and Obstetrics. 2005;272(2):131-5.

116. Eren A, Küçükercan M, Oǧuzoǧlu N, Ünal N, Karateke A. The carriage of group B streptococci in Turkish pregnant women and its transmission rate in newborns and serotype distribution. Turkish Journal of Pediatrics. 2005;47(1):28-33.

117. Amin A, Abdulrazzaq YM, Uduman S. Group B streptococcal serotype distribution of isolates from colonized pregnant women at the time of delivery in United Arab Emirates. Journal of Infection. 2002;45(1):42-6.

118. Deutscher M, Lewis M, Zell ER, Taylor TH, Jr., Van Beneden C, Schrag S. Incidence and severity of invasive Streptococcus pneumoniae, group A Streptococcus, and group B Streptococcus infections among pregnant and postpartum women. Clin Infect Dis. 2011;53(2):114-23.

119. Phares CR, Lynfield R, Farley MM, Mohle-Boetani J, Harrison LH, Petit S, et al. Epidemiology of invasive group B streptococcal disease in the United States, 1999-2005. JAMA - Journal of the American Medical Association. 2008;299(17):2056-65.

120. Zaleznik DF, Rench MA, Hillier S, Krohn MA, Platt R, Lee MLT, et al. Invasive disease due to group B streptococcus in pregnant women and neonates from diverse population groups. Clinical Infectious Diseases. 2000;30(2):276-81.

121. Tyrrell GJ, Senzilet LD, Spika JS, Kertesz DA, Alagaratnam M, Lovgren M, et al. Invasive disease due to group B streptococcal infection in adults: Results from a Canadian, population-based, active laboratory surveillance study - 1996. Journal of Infectious Diseases. 2000;182(1):168-73.

122. Pass MA, Gray BM, Dillon HC, Jr. Puerperal and perinatal infections with group B streptococci. American Journal of Obstetrics & Gynecology. 1982;143(2):147-52.

123. Lu B, Wu J, Chen X, Gao C, Yang J, Li Y, et al. Microbiological and clinical characteristics of Group B Streptococcus isolates causing materno-neonatal infections: high prevalence of CC17/PI-1 and PI-2b sublineage in neonatal infections. J Med Microbiol. 2018;67(11):1551-9.

124. Davies HD, Raj S, Adair C, Robinson J, McGeer A. Population-based active surveillance for neonatal group B streptococcal infections in Alberta, Canada: Implications for vaccine formulation. Pediatric Infectious Disease Journal. 2001;20(9):879-84.

125. Menendez C, Castillo P, Martinez MJ, Jordao D, Lovane L, Ismail MR, et al. Validity of a minimally invasive autopsy for cause of death determination in stillborn babies and neonates in Mozambique: An observational study. PLoS Med. 2017;14(6):e1002318.

126. O'Sullivan CP. Group B streptococcal (GBS) disease in uk and irish infants younger than 90 days, 2014-2015. Archives of Disease in Childhood. 2018;103 (Supplement 1):A197.

127. Nanduri SA, Petit S, Smelser C, Apostol M, Alden NB, Harrison LH, et al. Epidemiology of Invasive Early-Onset and Late-Onset Group B Streptococcal Disease in the United States, 2006 to 2015: Multistate Laboratory and Population-Based Surveillance. JAMA Pediatr. 2019;173(3):224-33.

128. Romain AS, Cohen R, Plainvert C, Joubrel C, Bechet S, Perret A, et al. Clinical and Laboratory Features of Group B Streptococcus Meningitis in Infants and Newborns: Study of 848 Cases in France, 2001-2014. Clinical Infectious Diseases. 2018;66(6):857-64.

129. Creti R, Imperi M, Berardi A, Pataracchia M, Recchia S, Alfarone G, et al. Neonatal Group B Streptococcus Infections: Prevention Strategies, Clinical and Microbiologic Characteristics in 7 Years of Surveillance. Pediatr Infect Dis J. 2017;36(3):256-62.

130. Hayes K, Cotter L, Barry L, O'Halloran F. Emergence of the L phenotype in Group B Streptococci in the South of Ireland. Epidemiology & Infection. 2017;145(16):3535-42.

131. Martins ER, Pedroso-Roussado C, Melo-Cristino J, Ramirez M, Oliveira H, Vaz T, et al. Streptococcus agalactiae causing neonatal infections in Portugal (2005-2015): Diversification and emergence of a CC17/PI-2b multidrug resistant sublineage. Frontiers in Microbiology. 2017;8 (MAR) (no pagination)(499).

132. Six A, Firon A, Plainvert C, Caplain C, Touak G, Dmytruk N, et al. Molecular Characterization of Nonhemolytic and Nonpigmented Group B Streptococci Responsible for Human Invasive Infections. Journal of Clinical Microbiology. 2016;54(1):75-82.

133. Alhhazmi A, Hurteau D, Tyrrell GJ. Epidemiology of Invasive Group B Streptococcal Disease in Alberta, Canada, from 2003 to 2013.[Erratum appears in J Clin Microbiol. 2016 Dec 28;55(1):342-343; PMID: 28031447]. Journal of Clinical Microbiology. 2016;54(7):1774-81.

134. Almeida A, Villain A, Joubrel C, Touak G, Sauvage E, Rosinski-Chupin I, et al. Whole-Genome Comparison Uncovers Genomic Mutations between Group B Streptococci Sampled from Infected Newborns and Their Mothers. J Bacteriol. 2015;197(20):3354-66.

135. Joubrel C, Tazi A, Six A, Dmytruk N, Touak G, Bidet P, et al. Group B streptococcus neonatal invasive infections, France 2007-2012. Clin Microbiol Infect. 2015;21(10):910-6.

136. Chang B, Wada A, Hosoya M, Oishi T, Ishiwada N, Oda M, et al. Characteristics of group B Streptococcus isolated from infants with invasive infections: a population-based study in Japan. Jpn J Infect Dis. 2014;67(5):356-60.

137. Brzychczy-Wloch M, Gosiewski T, Bulanda M. Multilocus sequence types of invasive and colonizing neonatal group B streptococci in Poland. Med Princ Pract. 2014;23(4):323-30.

138. Teatero S, McGeer A, Low DE, Li A, Demczuk W, Martin I, et al. Characterization of invasive group B streptococcus strains from the greater Toronto area, Canada. J Clin Microbiol. 2014;52(5):1441-7.

139. Bekker V, Bijlsma MW, van de Beek D, Kuijpers TW, van der Ende A. Incidence of invasive group B streptococcal disease and pathogen genotype distribution in newborn babies in the Netherlands over 25 years: a nationwide surveillance study. Lancet Infect Dis. 2014;14(11):1083-9.

140. Morozumi M, Wajima T, Kuwata Y, Chiba N, Sunaoshi K, Sugita K, et al. Associations between capsular serotype, multilocus sequence type, and macrolide resistance in Streptococcus agalactiae isolates from Japanese infants with invasive infections. Epidemiol Infect. 2014;142(4):812-9.

141. Matsubara K, Hoshina K, Suzuki Y. Early-onset and late-onset group B streptococcal disease in Japan: a nationwide surveillance study, 2004-2010. Int J Infect Dis. 2013;17(6):e379-84.

142. Oladottir GL, Erlendsdottir H, Palsson G, Bjornsdottir ES, Kristinsson KG, Haraldsson A. Increasing incidence of late-onset neonatal invasive group B streptococcal infections in Iceland. Pediatr Infect Dis J. 2011;30(8):661-3.

143. Imperi M, Gherardi G, Berardi A, Baldassarri L, Pataracchia M, Dicuonzo G, et al. Invasive neonatal GBS infections from an area-based surveillance study in Italy. Clin Microbiol Infect. 2011;17(12):1834-9.

144. Martins ER, Andreu A, Correia P, Juncosa T, Bosch J, Ramirez M, et al. Group B streptococci causing neonatal infections in barcelona are a stable clonal population: 18-year surveillance. J Clin Microbiol. 2011;49(8):2911-8.

145. Matsubara K, Yamamoto G. Invasive group B streptococcal infections in a tertiary care hospital between 1998 and 2007 in Japan. International Journal of Infectious Diseases. 2009;13(6):679-84.

146. Zhao Z, Kong F, Zeng X, Gidding HF, Morgan J, Gilbert GL. Distribution of genotypes and antibiotic resistance genes among invasive Streptococcus agalactiae (group B streptococcus) isolates from Australasian patients belonging to different age groups. Clin Microbiol Infect. 2008;14(3):260-7.

147. Trijbels-Smeulders MA, Kimpen JL, Kollee LA, Bakkers J, Melchers W, Spanjaard L, et al. Serotypes, genotypes, and antibiotic susceptibility profiles of group B streptococci causing neonatal sepsis and meningitis before and after introduction of antibiotic prophylaxis. Pediatr Infect Dis J. 2006;25(10):945-8.

148. Fluegge K, Supper S, Siedler A, Berner R. Serotype distribution of invasive group B streptococcal isolates in infants: results from a nationwide active laboratory surveillance study over 2 years in Germany. Clin Infect Dis. 2005;40(5):760-3.

149. Davies HD, Jones N, Whittam TS, Elsayed S, Bisharat N, Baker CJ. Multilocus sequence typing of serotype III group B streptococcus and correlation with pathogenic potential. J Infect Dis. 2004;189(6):1097-102.

150. Strakova L, Motlova J. Active surveillance of early onset disease due to group B streptococci in newborns. Indian J Med Res. 2004;119 Suppl:205-7.

151. Figueira-Coelho J, Ramirez M, Salgado MJ, Melo-Cristino J. Streptococcus agalactiae in a large Portuguese teaching hospital: antimicrobial susceptibility, serotype distribution, and clonal analysis of macrolide-resistant isolates. Microbial Drug Resistance-Mechanisms Epidemiology & Disease. 2004;10(1):31-6.

152. Persson E, Berg S, Trollfors B, Larsson P, Ek E, Backhaus E, et al. Serotypes and clinical manifestations of invasive group B streptococcal infections in western Sweden 1998-2001. Clinical Microbiology & Infection. 2004;10(9):791-6.

153. Ekelund K, Konradsen HB. Invasive group B streptococcal disease in infants: a 19-year nationwide study. Serotype distribution, incidence and recurrent infection. Epidemiol Infect. 2004;132(6):1083-90.

154. Bidet P, Brahimi N, Chalas C, Aujard Y, Bingen E. Molecular characterization of serotype III group B-streptococcus isolates causing neonatal meningitis. J Infect Dis. 2003;188(8):1132-7.

155. Hoshina K, Suzuki Y, Nishida H, Kaneko K, Matsuda S, Kobayashi M, et al. Trend of neonatal group B streptococcal infection during the last 15 years. Pediatr Int. 2002;44(6):641-6.

156. Guan X, Mu X, Ji W, Yuan C, He P, Zhang L, et al. Epidemiology of invasive group B streptococcal disease in infants from urban area of South China, 2011-2014. BMC Infectious Diseases. 2018;18 (1) (no pagination)(14).

157. Liu J, Xu R, Zhong H, Zhong Y, Xie Y, Li L, et al. Prevalence of GBS serotype III and identification of a ST 17-like genotype from neonates with invasive diseases in Guangzhou, China. Microbial Pathogenesis. 2018;120:213-8.

158. Cho HK, Nam HN, Cho HJ, Son DW, Cho YK, Seo YH, et al. Serotype distribution of invasive group B streptococcal diseases in infants at two university hospitals in Korea. [Korean]. Pediatric Infection and Vaccine. 2017;24(2):79-86.

159. Lo CW, Liu HC, Lee CC, Lin CL, Chen CL, Jeng MJ, et al. Serotype distribution and clinical correlation of Streptococcus agalactiae causing invasive disease in infants and children in Taiwan. International Journal of Antimicrobial Agents. 2017;50 (Supplement 2):S203-S4.

160. Ip M, Ang I, Fung K, Liyanapathirana V, Luo MJ, Lai R. Hypervirulent Clone of Group B Streptococcus Serotype III Sequence Type 283, Hong Kong, 1993-2012. Emerg Infect Dis. 2016;22(10):1800-3.

161. Wang P, Ma Z, Tong J, Zhao R, Shi W, Yu S, et al. Serotype distribution, antimicrobial resistance, and molecular characterization of invasive group B Streptococcus isolates recovered from Chinese neonates. Int J Infect Dis. 2015;37:115-8.

162. Yoon IA, Jo DS, Cho EY, Choi EH, Lee HJ, Lee H. Clinical significance of serotype V among infants with invasive group B streptococcal infections in South Korea. International Journal of Infectious Diseases. 2015;38:136-40.

163. Rivera L, Saez-Llorens X, Feris-Iglesias J, Ip M, Saha S, Adrian PV, et al. Incidence and serotype distribution of invasive group B streptococcal disease in young infants: a multi-country observational study. BMC Pediatr. 2015;15:143.

164. Souza VC, Kegele FCO, Souza SR, Neves FPG, De Paula GR, Barros RR. Antimicrobial susceptibility and genetic diversity of Streptococcus agalactiae recovered from newborns and pregnant women in Brazil. Scandinavian Journal of Infectious Diseases. 2013;45(10):780-5.

165. Fiolo K, Zanardi CE, Salvadego M, Bertuzzo CS, Amaral E, Calil R, et al. [Infection rate and Streptococcus agalactiae serotypes in samples of infected neonates in the city of Campinas (Sao Paulo), Brazil]. Rev Bras Ginecol Obstet. 2012;34(12):544-9.

166. Martinez TMA, Ovalle SA, Duran TC, Reid SI, Urriola JG, Garay GB, et al. Serotypes and antimicrobial susceptibility of Streptococcus agalactiae. [Spanish]. Revista Medica de Chile. 2004;132(5):549-55.

167. Lopardo HA, Vidal P, Jeric P, Centron D, Paganini H, Facklam RR, et al. Six-month multicenter study on invasive infections due to group B streptococci in Argentina. Journal of Clinical Microbiology. 2003;41(10):4688-94.

168. Sigauque B, Kobayashi M, Vubil D, Nhacolo A, Chauque A, Moaine B, et al. Invasive bacterial disease trends and characterization of group B streptococcal isolates among young infants in southern Mozambique, 2001-2015. PLoS ONE [Electronic Resource]. 2018;13(1):e0191193.

169. Dangor Z, Cutland CL, Izu A, Kwatra G, Trenor S, Lala SG, et al. Temporal Changes in Invasive Group B Streptococcus Serotypes: Implications for Vaccine Development. PLOS ONE. 2016;11(12):e0169101.

170. Cutland CL, Schrag SJ, Thigpen MC, Velaphi SC, Wadula J, Adrian PV, et al. Increased risk for group b streptococcus sepsis in young infants exposed to hiv, soweto, south africa, 2004-20081 United States: Centers for Disease Control and Prevention (CDC); 2015 [cited 21 (Cutland, Velaphi, Wadula, Adrian, Kuwanda, Groome, Buchmann, Madhi) University of the Witwatersrand, Johannesburg, South Africa]. 4:[638-45]. Available from: http://wwwnc.cdc.gov/eid/article/21/4/pdfs/14-1562.pdf

<http://ovidsp.ovid.com/ovidweb.cgi?T=JS&PAGE=reference&D=emed13&NEWS=N&AN=2015839003>.

171. Lopes E, Fernandes T, Machado MP, Carrico J, Cristino JM, Ramirez M, et al. Increase of macrolide resistance among group B Streptococcus invasive disease in non-pregnant adults in Portugal (2009- 2015) was driven by a capsular variant of a single clone. International Journal of Antimicrobial Agents. 2017;50 (Supplement 2):S116.

172. Bjornsdottir ES, Martins ER, Erlendsdottir H, Haraldsson G, Melo-Cristino J, Kristinsson KG, et al. Changing epidemiology of group B streptococcal infections among adults in Iceland: 1975-2014. Clinical Microbiology and Infection. 2016;22(4):379.e9-.e16.

173. Martins ER, Melo-Cristino J, Ramirez M, Lito L, Monteiro L, Martins F, et al. Dominance of serotype Ia among group B streptococci causing invasive infections in nonpregnant adults in Portugal. Journal of Clinical Microbiology. 2012;50(4):1219-27.

174. Kothari NJ, Morin CA, Glennen A, Jackson D, Harper J, Schrag SJ, et al. Invasive group B streptococcal disease in the elderly, Minnesota, USA, 2003-2007. Emerging Infectious Diseases. 2009;15(8):1279-81.

175. Skoff TH, Farley MM, Petit S, Craig AS, Schaffner W, Gershman K, et al. Increasing Burden of Invasive Group B Streptococcal Disease in Nonpregnant Adults, 1990-2007. Clinical Infectious Diseases. 2009;49(1):85-92.

176. Wessels MR, Kasper DL, Johnson KD, Harrison LH. Antibody responses in invasive group B streptococcal infection in adults. Journal of Infectious Diseases. 1998;178(2):569-72.

177. Harrison LH, Ali A, Dwyer DM, Libonati JP, Reeves MW, Elliott JA, et al. Relapsing invasive group B streptococcal infection in adults. Annals of Internal Medicine. 1995;123(6):421-7.

178. Eskandarian N, Neela V, Ismail Z, Puzi SM, Hamat RA, Desa MN, et al. Group B streptococcal bacteremia in a major teaching hospital in Malaysia: a case series of eighteen patients. International Journal of Infectious Diseases. 2013;17(9):e777-80.

179. Beauruelle C, Pastuszka A, Mereghetti L, Lanotte P. Group B Streptococcus Vaginal Carriage in Pregnant Women as Deciphered by Clustered Regularly Interspaced Short Palindromic Repeat Analysis. J Clin Microbiol. 2018;56(6).

180. Dmitriev A, Hu YY, Shen AD, Suvorov A, Yang YH. Chromosomal analysis of group B streptococcal clinical strains; bac gene-positive strains are genetically homogenous. FEMS Microbiol Lett. 2002;208(1):93-8.

181. Elikwu CJ, Oduyebo O, Ogunsola FT, Anorlu RI, Okoromah CN, König B. High group B streptococcus carriage rates in pregnant women in a tertiary institution in Nigeria. Pan Afr Med J. 2016;25:249.

182. Li S, Wen G, Cao X, Guo D, Yao Z, Wu C, et al. Molecular characteristics of Streptococcus agalactiae in a mother-baby prospective cohort study: Implication for vaccine development and insights into vertical transmission. Vaccine. 2018;36(15):1941-8.

183. Liakopoulos A, Mavroidi A, Vourli S, Panopoulou M, Zachariadou L, Chatzipanagiotou S, et al. Molecular characterization of Streptococcus agalactiae from vaginal colonization and neonatal infections: A 4-year multicenter study in Greece. Diagnostic Microbiology and Infectious Disease. 2014;78(4):487-90.

184. Mohamed Sadaka S, Abdelsalam Aly H, Ahmed Meheissen M, Orief YI, Mohamed Arafa B. Group B streptococcal carriage, antimicrobial susceptibility, and virulence related genes among pregnant women in Alexandria, Egypt. Alexandria Journal of Medicine. 2018;54(1):69-76.

185. Springman AC, Lacher DW, Waymire EA, Wengert SL, Singh P, Zadoks RN, et al. Pilus distribution among lineages of group b streptococcus: an evolutionary and clinical perspective. BMC Microbiology. 2014;14(1):159.

186. Madzivhandila M, Adrian PV, Cutland CL, Kuwanda L, Madhi SA, Po PSTT. Distribution of pilus islands of group B streptococcus associated with maternal colonization and invasive disease in South Africa. Journal of medical microbiology. 2013;62(Pt 2):249-53.

187. Manning SD, Springman AC, Lehotzky E, Lewis MA, Whittam TS, Davies HD. Multilocus sequence types associated with neonatal group B streptococcal sepsis and meningitis in Canada. J Clin Microbiol. 2009;47(4):1143-8.

188. Tsai MH, Hsu JF, Lai MY, Lin LC, Chu SM, Huang HR, et al. Molecular Characteristics and Antimicrobial Resistance of Group B Streptococcus Strains Causing Invasive Disease in Neonates and Adults. Front Microbiol. 2019;10:264.

189. Gajic I, Plainvert C, Kekic D, Dmytruk N, Mijac V, Tazi A, et al. Molecular epidemiology of invasive and non-invasive group B Streptococcus circulating in Serbia. Int J Med Microbiol. 2019;309(1):19-25.

190. Jones N, Oliver KA, Barry J, Harding RM, Bisharat N, Spratt BG, et al. Enhanced invasiveness of bovine-derived neonatal sequence type 17 group B Streptococcus is independent of capsular serotype United States: University of Chicago Press; 2006 [cited 42 (Jones, Oliver, Barry, Bisharat, Crook) Infectious Disease and Microbiology, John Radcliffe Hospital, University of Oxford, Oxford, United Kingdom]. 7:[915-24]. Available from: <http://ovidsp.ovid.com/ovidweb.cgi?T=JS&PAGE=reference&D=emed7&NEWS=N&AN=2006138296>.

191. Emaneini M, Jabalameli F, Mirsalehian A, Ghasemi A, Beigverdi R. Characterization of virulence factors, antimicrobial resistance pattern and clonal complexes of group B streptococci isolated from neonates. Microb Pathog. 2016;99:119-22.

192. Guo D, Cao X, Li S, Ou Q, Lin D, Yao Z, et al. Neonatal colonization of group B Streptococcus in China: Prevalence, antimicrobial resistance, serotypes, and molecular characterization. American Journal of Infection Control. 2018.

193. Hsu JF, Chen CL, Lee CC, Lien R, Chu SM, Fu RH, et al. Characterization of group B Streptococcus colonization in full-term and Late-Preterm neonates in Taiwan. Pediatr Neonatol. 2019;60(3):311-7.

194. Toyofuku M, Morozumi M, Hida M, Satoh Y, Sakata H, Shiro H, et al. Effects of Intrapartum Antibiotic Prophylaxis on Neonatal Acquisition of Group B Streptococci. Journal of Pediatrics. 2017;190:169-73.e1.

195. Al Safadi R, Amor S, Hery-Arnaud G, Spellerberg B, Lanotte P, Mereghetti L, et al. Enhanced expression of lmb gene encoding laminin-binding protein in Streptococcus agalactiae strains harboring IS1548 in scpB-lmb intergenic region. PLoS One. 2010;5(5):e10794.

196. Bellais S, Six A, Fouet A, Longo M, Dmytruk N, Glaser P, et al. Capsular switching in group B Streptococcus CC17 hypervirulent clone: a future challenge for polysaccharide vaccine development. J Infect Dis. 2012;206(11):1745-52.

197. Bergseng H, Afset JE, Radtke A, Loeseth K, Lyng RV, Rygg M, et al. Molecular and phenotypic characterization of invasive group B streptococcus strains from infants in Norway 2006-2007. Clin Microbiol Infect. 2009;15(12):1182-5.

198. Bergseng H, Rygg M, Bevanger L, Bergh K. Invasive group B streptococcus (GBS) disease in Norway 1996-2006. European Journal of Clinical Microbiology and Infectious Diseases. 2008;27(12):1193-9.

199. Björnsdóttir ES, Martins ER, Erlendsdóttir H, Haraldsson G, Melo-Cristino J, Ramirez M, et al. Group B Streptococcal Neonatal and Early Infancy Infections in Iceland, 1976-2015. Pediatr Infect Dis J. 2019;38(6):620-4.

200. Campisi E, Rosini R, Ji W, Guidotti S, Rojas-López M, Geng G, et al. Genomic Analysis Reveals Multi-Drug Resistance Clusters in Group B Streptococcus CC17 Hypervirulent Isolates Causing Neonatal Invasive Disease in Southern Mainland China. Frontiers in microbiology. 2016;7:1265-.

201. Fluegge K, Wons J, Spellerberg B, Swoboda S, Siedler A, Hufnagel M, et al. Genetic differences between invasive and noninvasive neonatal group B streptococcal isolates. Pediatr Infect Dis J. 2011;30(12):1027-31.

202. Gherardi G, Imperi M, Baldassarri L, Pataracchia M, Alfarone G, Recchia S, et al. Molecular epidemiology and distribution of serotypes, surface proteins, and antibiotic resistance among group B streptococci in Italy. Journal of Clinical Microbiology. 2007;45(9):2909-16.

203. Jauneikaite E, Kapatai G, Davies F, Gozar I, Coelho J, Bamford KB, et al. Serial Clustering of Late-Onset Group B Streptococcal Infections in the Neonatal Unit: A Genomic Re-evaluation of Causality. Clin Infect Dis. 2018;67(6):854-60.

204. Kang HM, Lee HJ, Lee H, Jo DS, Lee HS, Kim TS, et al. Genotype Characterization of Group B Streptococcus Isolated from Infants with Invasive Diseases in South Korea. Pediatric Infectious Disease Journal. 2017;36(10):e242-e7.

205. Luan S-L, Granlund M, Sellin M, Lagergard T, Spratt BG, Norgren M. Multilocus sequence typing of Swedish invasive group B streptococcus isolates indicates a neonatally associated genetic lineage and capsule switching. Journal of clinical microbiology. 2005;43(8):3727-33.

206. MacFarquhar JK, Jones TF, Woron AM, Kainer MA, Whitney CG, Beall B, et al. Outbreak of late-onset group B Streptococcus in a neonatal intensive care unit. Am J Infect Control. 2010;38(4):283-8.

207. Manning SD, Ki M, Marrs CF, Kugeler KJ, Borchardt SM, Baker CJ, et al. The frequency of genes encoding three putative group B streptococcal virulence factors among invasive and colonizing isolates United Kingdom: BioMed Central Ltd.; 2006 [cited 6 (Manning) National Food Safety and Toxicology Center, Department of Pediatrics and Human Development, Michigan State University, East Lansing, MI, United States]. Available from: <http://www.biomedcentral.com/1471-2334/6/116>

<http://ovidsp.ovid.com/ovidweb.cgi?T=JS&PAGE=reference&D=emed7&NEWS=N&AN=2006426525>.

208. Margarit I, Rinaudo CD, Galeotti CL, Maione D, Ghezzo C, Buttazzoni E, et al. Preventing bacterial infections with pilus-based vaccines: the group B streptococcus paradigm. J Infect Dis. 2009;199(1):108-15.

209. Persson E, Berg S, Bevanger L, Bergh K, Valso-Lyng R, Trollfors B. Characterisation of invasive group B streptococci based on investigation of surface proteins and genes encoding surface proteins. Clinical Microbiology and Infection. 2008;14(1):66-73.

210. Poyart C, Réglier-Poupet H, Tazi A, Billoët A, Dmytruk N, Bidet P, et al. Invasive group B streptococcal infections in infants, France. Emerg Infect Dis. 2008;14(10):1647-9.

211. Puopolo KM, Madoff LC. Type IV Neonatal Early-Onset Group B Streptococcal Disease in a United States Hospital. Journal of Clinical Microbiology. 2007;45(4):1360-2.

212. Salloum M, van der Mee-Marquet N, Valentin-Domelier AS, Quentin R. Diversity of prophage DNA regions of streptococcus agalactiae clonal lineages from adults and neonates with invasive infectious disease. PLoS ONE. 2011;6 (5) (no pagination)(e20256).

213. Sigauque B, Kobayashi M, Vubil D, Nhacolo A, Chauque A, Moaine B, et al. Invasive bacterial disease trends and characterization of group B streptococcal isolates among young infants in southern Mozambique, 2001-2015. PLoS ONE. 2018;13 (1) (no pagination)(e0191193).

214. Tien N, Ho C-M, Lin H-J, Shih M-C, Ho M-W, Lin H-C, et al. Multilocus sequence typing of invasive group B Streptococcus in central area of Taiwan. Journal of microbiology, immunology, and infection = Wei mian yu gan ran za zhi. 2011;44(6):430-4.

215. van der Mee-Marquet N, Diene SM, Barbera L, Courtier-Martinez L, Lafont L, Ouachée A, et al. Analysis of the prophages carried by human infecting isolates provides new insight into the evolution of Group B Streptococcus species. Clin Microbiol Infect. 2018;24(5):514-21.

216. Veeraraghavan B, Devanga Ragupathi NK, Santhanam S, Verghese VP, Inbanathan FY, Livingston C. Whole genome shotgun sequencing of Indian strains of Streptococcus agalactiae. Genom Data. 2017;14:63-5.

217. Wu B, Su J, Li L, Wu W, Wu J, Lu Y, et al. Phenotypic and genetic differences among group B Streptococcus recovered from neonates and pregnant women in Shenzhen, China: 8-year study. BMC Microbiol. 2019;19(1):185.

218. Sadowy E, Matynia B, Hryniewicz W. Population structure, virulence factors and resistance determinants of invasive, non-invasive and colonizing Streptococcus agalactiae in Poland. Journal of Antimicrobial Chemotherapy. 2010;65(9):1907-14.

219. Florindo C, Gomes JP, Rato MG, Bernardino L, Spellerberg B, Santos-Sanches I, et al. Molecular epidemiology of group B streptococcal meningitis in children beyond the neonatal period from Angola. J Med Microbiol. 2011;60(Pt 9):1276-80.

220. Gabrielsen C, Mæland JA, Lyng RV, Radtke A, Afset JE. Molecular characteristics of Streptococcus agalactiae strains deficient in alpha-like protein encoding genes. J Med Microbiol. 2017;66(1):26-33.

221. Lu B, Chen X, Wang J, Wang D, Zeng J, Li Y, et al. Molecular characteristics and antimicrobial resistance in invasive and noninvasive Group B Streptococcus between 2008 and 2015 in China. Diagnostic Microbiology and Infectious Disease. 2016;86(4):351-7.

222. Morozumi M, Wajima T, Takata M, Iwata S, Ubukata K. Molecular characteristics of group B streptococci isolated from adults with invasive infections in Japan. Journal of Clinical Microbiology. 2016;54(11):2695-700.

223. Ryu H, Park YJ, Kim YK, Chang J, Yu JK. Dominance of clonal complex 10 among the levofloxacin-resistant Streptococcus agalactiae isolated from bacteremic patients in a Korean hospital. J Infect Chemother. 2014;20(8):509-11.

224. Luan SL, Granlund M, Sellin M, Lagergard T, Spratt BG, Norgren M. Multilocus sequence typing of Swedish invasive group B streptococcus isolates indicates a neonatally associated genetic lineage and capsule switching. Journal of Clinical Microbiology. 2005;43(8):3727-33.

225. Tien N, Ho CM, Lin HJ, Shih MC, Ho MW, Lin HC, et al. Multilocus sequence typing of invasive group B Streptococcus in central area of Taiwan. Journal of Microbiology, Immunology and Infection. 2011;44(6):430-4.
